# Physicochemical Properties, Antioxidant and Antibacterial Activities and Anti-Hepatocarcinogenic Effect and Potential Mechanism of *Schefflera oleifera* Honey Against HepG2 Cells

**DOI:** 10.3390/foods14132376

**Published:** 2025-07-04

**Authors:** Jingjing Li, Jie Wang, Yicong Wang, Wenchao Yang

**Affiliations:** College of Bee Science and Biomedicine, Fujian Agriculture and Forestry University, Fuzhou 350002, China; lijingjing000407@163.com (J.L.); wangjie01092023@163.com (J.W.); 18265526098@163.com (Y.W.)

**Keywords:** *Schefflera oleifera* honey, physicochemical characteristics, antioxidant activity, antibacterial activity, antitumor, network pharmacology

## Abstract

*Schefflera oleifera* honey (SH) is produced from the nectar of *S. Oleifera* by worker bees. Due to its unique properties and potential biological activities, this winter honey has attracted much attention. In this study, the physicochemical characteristics, antioxidant and antibacterial activities, antitumor effect against HepG2 cells, and its potential mechanisms of SH were systematically evaluated. The results showed that different SH samples differed significantly in their physicochemical characteristics. The 910 chemical components, including 52 kinds of phenols, phenolic acids, and flavonoids, were detected in the methanol extract of SH using UHPLC-MS/MS by non-targeted metabolomics. Based on our limited knowledge, solanine and soyasaponin I are the first determined components in honey, and they may be used as characteristic substances of SH for identification and adulteration. SH had a weaker inhibitory effect against *Salmonella typhimurium* and *Staphylococcus aureus* than MH (UMF 10+), analyzed by MBC and MIC assays. Network pharmacology analysis showed that 95 overlapping targets were found between the active ingredients of SH and liver cancer cells (HepG2), which were enriched in KEGG of the PI3K-Akt pathway, Lipid and atherosclerosis, Proteoglycans in cancer, etc. The IC_50_ of SH against HepG2 cells was 5.07% (dw/v), which is lower than the glucose, fructose, and sucrose contents in SH on HepG2 cells, of 16.24%, 9.60% dw/v, and 9.94% dw/v, respectively. SH significantly down-regulated the expression of EGFR, AKT1, and SRC in HepG2 cells (*p* < 0.05), determined by an enzyme-linked immunosorbent assay kit, and induced cell cycle arrest and apoptosis by multiple pathways. These results provide a theoretical basis for its potential application in developing functional foods and additives.

## 1. Introduction

Honey is produced from nectar or secretions of plants by worker bees, of which the process includes foraging, transformation, deposition, dehydration, and storage. It contains about 200 components, including sugars, water, proteins, amino acids, vitamins, minerals, enzymes, and phenolic compounds [1,2]. Honey is widely used as a food additive and as a drug to treat diseases and wounds [3,4,5]. Its biological activities include anti-cancer [6,7,8,9], anti-inflammatory [10,11,12], antibacterial [13,14,15], antioxidant [16,17,18], antiviral [4], and prebiotic activities [3]. The biological activities of honey are affected by many factors.

The antioxidant activity of different honey samples varies depending on their botanical origin [19], geographical region [20], processing and storage conditions [21], and the method used to measure the antioxidant activity [22]. Methods used to measure antioxidant activity include 2,2′-azino-bis-3-ethylbenzothiazolin-6-sulphonic acid (ABTS) [22], 2,2-diphenyl-1-picrylhydrazyl free radical (DPPH) [23], and Ferric Ion Reducing Antioxidant Power (FRAP) [24]. Phenolic compounds are the main source of the antioxidant activity of honey [19,25,26,27]. The total flavonoid content, total water-soluble vitamins and minerals, and protein content of honey also contribute to the antioxidant activity [19,20,24]. The botanical source of honey is closely related to its antioxidant properties, which is one of the mechanisms of antibacterial activity.

The antimicrobial effects of honey were attributed primarily to its high sugar concentration, antioxidant activity, acidic components, and production of hydrogen peroxide [13,15,28,29]. High osmotic pressure inhibits microbial growth because sugar molecules bind to water molecules, leaving bacteria without sufficient water to survive [30,31,32]. The acidic components of honey (partly due to its gluconic acid) neutralize the alkaline environment of chronic wounds, thereby reducing protease activity, increasing fibroblast activity, and increasing oxygen release [33,34], all of which are beneficial to the wound healing process. Hydrogen peroxide and non-hydrogen peroxide compounds are produced by glucose oxidase added by bees during the maturation process of honey [15,28,29]. The hydrogen peroxide gradient attracts macrophages to the wound and releases vascular endothelial growth factors and angiogenic factors, which are essential for wound healing [35,36,37,38]. Honey provides a steady supply of hydrogen peroxide to exhibit antimicrobial and physiologically nontoxic effects [39] when the honey is diluted. Also, the high sugar content in honey attracts liquid and forms a protective layer that prevents microbial invasion and keeps the wound dry [40,41,42,43,44]. *Schefflera oleifera* honey (SH), characterized by its light amber color and slightly bitter taste, is a typical winter honey produced primarily in the Fujian, Guangxi, and Guangdong provinces of southern China [45]. Although studies have investigated the antioxidant activity of SH [46], its antimicrobial and anti-hepatocarcinogenic properties have not been fully investigated.

Hepatocellular carcinoma (HCC) is one of the most common liver cancers. Numerous factors, including infection with hepatitis C or hepatitis B virus, obesity, diabetes, and genetic and social risk factors (such as excessive alcohol consumption) increase the incidence of HCC [47]. Despite significant advances in the treatment of HCC, the quality of life of patients remains poor. This suggests that more effective treatments should be achieved. Honey has strong and specific cytotoxicity to tumor cells and no cytotoxicity for normal cells. Egyptian clover honey significantly reduced the number of viable human hepatocellular carcinoma (HepG2) cells and nitric oxide (NO) levels [48]. Based on these findings, reactive oxygen species (ROS) may play a key role in the survival of HepG2 cell lines. Moderate levels of reactive oxygen species contribute to cell growth, division, and proliferation. The IC_50_ values of manuka honey were 6.92 ± 0.005% and 18.62 ± 0.07% for HepG2 and Hep3B cells, respectively [49]. A combination of selected drugs (Cisplatin, cyclophosphamide, and 5-Fluorouracil) and *Nigella sativa* honey may be an effective chemo-preventive and therapeutic strategy for treating diethylnitrosamine (DEN) and CCl4-induced HCC [50]. As an emerging research method, network pharmacology provides a powerful tool for understanding drug formulations and predicting potential new drugs or disease targets based on disciplines such as systems biology, genomics, and proteomics [51].

This study aims to explore the function of winter honey, SH, and its physicochemical properties, antioxidant activities, and antibacterial activities, as well as predict its targets and signaling pathways for the inhibition of HepG2 cells using network pharmacology methods and validation experiments. The results can provide a scientific basis and theoretical support for the development and utilization of SH in food additives and functional foods.

## 2. Materials and Methods

### 2.1. Honey Samples, Bacteria and Cells

SH samples were harvested from different bee farms in Zhangzhou, Fujian Province, China, in January 2024. Manuka honey (MH, UMF 10+) was purchased from Comvita New Zealand Ltd., (Paengaroa, New Zealand) in August 2019 and mailed to our laboratory. These honey samples were stored in a 4 °C refrigerator until the experiments.

*Escherichia coli* and *Staphylococcus aureus* were purchased from the Guangdong Microbiological Culture Collection Center. *Listeria monocytogenes* was isolated from the Microbiological Laboratory of Fujian Agriculture and Forestry University. *Salmonella typhimurium* ATCC 14028 was purchased from Guangdong Huankai Microbiology Co., Ltd., Zhaoqing, China. The HepG2 (CL-0103) cell line was purchased from Wuhan Procell Life Science and Technology (Wuhan, China).

Chemicals not otherwise specified were purchased from Sinopharm Chemical Reagent Co., Ltd., Shanghai, China.

### 2.2. Determination of Physicochemical Properties of SH Samples

The contents of water, pH, glucose, fructose, and sucrose in SH samples were determined according to the methods of our earlier report [52]. The total phenolic and total flavonoid contents were determined according to previous methods [53]. SH (1.0 g, dw) was diluted with ultrapure water to an amount of 10.0 mL. SH aqueous solution (1.0 mL) was mixed with 1.0 mL of Folin–Ciocalteu color reagent for 3–5 min. Sodium carbonate solution (20%, 3 mL) was diluted to 10 mL and mixed well. After being held at room temperature in the dark for 1 h, ultrapure water was selected as a blank control to measure the absorbance at 760 nm. Using gallic acid (10–100 mg/mL) as the standard, the results were expressed in gallic acid equivalents as mg GAE/100 g. SH aqueous solution (1.0 mL) was mixed with 0.5 mL of 5% sodium nitrite. The mixture was shaken well and stilled for 6 min. Aluminum nitrate (10%, 0.5 mL) was added and mixed for 6 min. Then, 4 mL of 4% sodium hydroxide was added and the mixture was shaken well and diluted to 10 mL with 80% methanol. It was mixed for 15 min. The absorbance at 510 nm was measured using enzyme-linked immunosorbent assay (ELISA) by a microplate reader (1510, Thermo Fisher Scientific, Waltham, MA, USA) using 80% methanol as a blank control. Rutin solution (10–100 mg/mL) was the standard solution; the results are expressed in rutin equivalents as mg RE/100 g. The data are shown based on the dry weight of each SH sample.

The methanol compositions of SH were determined using UHPLC-MS/MS by non-targeted metabolomics at Novogene Co., Ltd. (Beijing, China) [53]. Honey samples (100 μL) were placed in EP tubes and suspended in pre-cooled 80% methanol (Shanghai MacLean Biochemical Technology Co., Ltd., Shanghai, China) by vortexing. Then, the samples were incubated on ice for 5 min and centrifuged at 15,000× *g* and 4 °C for 20 min. The supernatant was diluted with LC-MS grade water (Merck, Darmstadt, Germany) to a final concentration of 53% methanol and then centrifuged at 15,000× *g* and 4 °C for 20 min. The supernatant was injected into the LC-MS/MS system for analysis. UHPLC-MS/MS analysis was performed using a Vanquish UHPLC system (Thermo Fisher Scientific Inc., Dreieich, Germany) coupled with a Q Exactive™ HF/Q Exactive™ HF-X mass spectrometer (Thermo Fisher, Germany). The sample was injected into a Hypersil Gold column (100 mm 2.1 mm, 1.9 μm) at a flow rate of 0.2 mL/min with a 17 min linear gradient. The eluents in the positive polarity mode were eluent A (0.1% formic acid in water) and eluent B (methanol). The eluents in the negative polarity mode were eluent A (5 M ammonium acetate, pH 9.0) and eluent B (methanol). The solvent gradient was set as follows: 2% mobile phase for 1.5 min, mobile phase A 2–85% for 3 min, mobile phase B 85–100% for 10 min, mobile phase B 2% for 10.1 min, and mobile phase B 2% for 12 min. The Q Exactive™ HF/Q Exactive™ HF-X mass spectrometer (Thermo Fisher Scientific Inc., Dreieich, Germany) was operated in positive/negative polarity mode with a spray voltage of 3.5 kV, a capillary temperature of 320 °C, a sheath gas flow rate of 35 psi, an auxiliary gas flow rate of 10 L/min, an iontophoresis RF level of 60, and an auxiliary gas heater temperature of 350 °C. The raw data files were acquired for further analysis.

### 2.3. Determination of Antioxidant Activity of SH

The DPPH free radical scavenging activity and FRAP total antioxidant capacity of the SH and MH samples were measured at 517 nm and 593 nm, respectively, using an ultraviolet spectrophotometer (T6, Beijing Puxi General Instrument Co., Ltd., Beijing, China) according to previous methods [54]. The ABTS cation radical scavenging activities of SH and MH samples were determined at 734 nm [55]. The results were expressed as equivalents to Trolox (mg TE/100 g, dw), which was employed as the standard.

All antioxidant activities were expressed as the dry weight (dw) of SH.

### 2.4. Determination of Antibacterial Activity of SH

SH and MH were mixed with LB broth medium (Sangon Biotech (Shanghai) Co., Ltd., Shanghai, China) and prepared in different concentrations of 0, 5%, 10%, 15%, 20%, 25%, 30%, 35%, 40%, 45%, and 50% (dry weight/volume of mixed liquid). These mediums were used to determine the antibacterial activity of SH and MH against *E. coli*, *S. aureus*, *L. monocytogenes*, and *S. typhimurium*.

#### 2.4.1. Determination of Minimum Inhibitory Concentration

Determination of the minimum inhibitory concentration (MIC) was performed according to a previous method [56]. The bacterial suspension was adjusted to 1 × 10^8^ CFU/mL with saline. The suspension (10 μL) was added into a 96-well plate. Then 190 μL of culture medium (broth medium containing SH or MH) was added and mixed thoroughly. It was incubated in a shaking incubator (HZQ-F100, Taicang Experimental Equipment Factory, Suzhou, China) at 37 °C and 80 rpm for 24 h. The mixed liquid was filtered with a 0.22 μm filter membrane (Biosharp Beijing Labgic Technology Co., Ltd., Beijing, China) for further determination of OD_600_ using an ELISA reader (1510, Thermo Fisher, Waltham, MA, USA).

#### 2.4.2. Determination of Minimum Bactericidal Concentration

The culture medium (200 μL) in the 96-well plate was transferred to LB solid medium (Sangon Biotech (Shanghai) Co., Ltd., China) and cultured at 37 °C for 24 h. The growth of colonies in the solid medium was observed. The lowest honey concentration at which no colonies was recorded as the MBC [56].

### 2.5. The Mechanism of SH Against HepG2 Cells Based on Network Pharmacology

#### 2.5.1. Retrieve the Targets of HepG2 and the Main Components of SH

The targets of the main components of SH were obtained from the EMBL-EBI (https://www.ebi.ac.uk/chembl/, accessed on 10 February 2025) and SEA Search Server (https://sea.bkslab.org/, accessed on 10 February 2025). The keyword-related targets were searched according to the keyword “Human hepatocellular carcinomas” in the “Genecard” database (https://www.genecards.org/, accessed on 10 February 2025). Effective targets with a score greater than 20 were screened. The target-related information was proofed in the UniProt database (https://www.uniprot.org/, accessed on 10 February 2025).

#### 2.5.2. Retrieve the Interaction Targets and Bioinformatics Analysis

The overlapping targets of the main SH components and HepG2 cells were screened. The GO (*p* < 0.05) enrichment and KEGG (*p* < 0.05) pathway enrichment analyses were performed using the clusterProfiler package (http://bioconductor.org/packages/release/bioc/html/clusterProfiler.html, downloaded on 10 February 2025) in R (v4.4, https://cloud.r-project.org/, accessed on 10 February 2025). The protein–protein interaction (PPI) network diagram with a confidence level ≥ 0.7 among the overlapping targets was obtained through the STRING database (string-db.org, accessed on 10 February 2025) and Cytoscape software (version 3.10.2; JAVA: 17.0.5, https://cytoscape.org/download.html, accessed on 15 February 2025).

#### 2.5.3. Experimental Verification

HepG2 cells were cultured with a complete culture medium (Wuhan Procell Life Science and Technology, China) in a constant temperature incubator (C150, Binder, Tuttlingen, Germany) at 37 °C and a CO_2_ concentration of 5%. The logarithmic growth phase HepG2 cells were processed with trypsin (Thermo Fisher Scientific Co., Ltd., Shanghai, China) and cultured in plates for subsequent experiments.

HepG2 cells (1 × 10^5^ cells/well) were transfered into wells of a six-well plate. After the cells adhered to the wall, the culture medium was removed. The cells were rinsed 3 times with PBS (pH 7.2–7.4, Sinopharm Chemical Reagent Co., Ltd., Shanghai, China). Then, 2%, 4%, 8%, and 16% SH (final concentration, dw/v) in the culture medium and complete culture medium (blank control) were added to each group. The cells were cultured for 48 h. These experiments were repeated in triplicate with 6 replicate wells for each group.

##### Determination of SRC, EGFR, and AKT1 Contents of HepG2 Cells

The contents of SRC, EGFR, and AKT1 in cell culture supernatant, which was obtained after centrifuging at 1000× *g* for 20 min, were determined using the enzyme-linked immunosorbent assay kit (Shanghai ELISA Biotechnology Co., Ltd., Shanghai, China) [57]. The different concentrations of standard solution and samples (50 μL) were mixed with 100 μL of a horseradish peroxidase (HRP)-labeled detection antibody. The reaction wells were sealed with a sealing film and incubated at 37 °C in a water bath for 60 min. The cells were patted dry using absorbent paper after the medium was removed. A washing solution (350 uL) was added and stilled for 1 min. The washing solution was removed and the cells were patted dry with absorbent paper again. This washing and drying procedure was repeated 5 times. Substrates A and B (each for 50 μL) were added to each well and incubated at 37 °C in the dark for 15 min. A stop solution (50 μL) was added to stop the reaction. The OD values of the wells at a wavelength of 450 nm were determined within 15 min using a microplate reader. Nothing was added to the blank control well. The blank well was set as 0 to draw a linear regression curve of the standard, in which the horizontal and vertical axes were the standard concentrations and the corresponding OD value, respectively. The concentrations of each sample were obtained according to the regression equation.

##### Antiproliferation Effect of SH5 and Saccharides on HepG2 Cells

The honey concentration was diluted to 2%, 4%, 8%, and 16% (dry weight/volume of mixed solution). Glucose, fructose, and sucrose stock solutions were prepared according to their contents of SH5, which has relatively excellent antioxidant and antibacterial activity and total phenolic and flavonoid contents among all samples. The glucose stock solution is equal to the glucose content plus the sucrose content, while the fructose stock solution is equal to the fructose content plus the sucrose content. The sucrose stock solution is equal to the glucose content plus half of the fructose content plus the sucrose content. The saccharide concentrations were set to 2%, 4%, 8%, 16%, and 32% (dw/v).

HepG2 cells at a concentration of 5 × 10^4^ cells/mL (100 μL) in a 96-well plate were incubated at 37 °C and 5% CO_2_ for 24 h. Cells were washed with PBS after the culture medium was removed. Complete culture mediums with different concentrations of SH or saccharide and complete culture medium (the control group) were added and incubated at 37 °C and 5% CO_2_ for 48 h. The wells with no cells were selected as the blank group. After 48 h, the cells were rinsed with PBS and 110 μL of complete culture medium containing 10 μL of CCK-8 solution was added (Beijing Solebao Technology Co., Ltd., Beijing, China), and the mixture was placed in the dark. The absorbance of each well at 450 nm was determined using the ELISA reader after 2 h of incubation [58]. There were 6 replicate wells in each group. The proliferation inhibition rate (%) was calculated as [(Ac − As)/(Ac − Ab)] × 100%, where As was the experimental group (cells, culture medium containing honey or saccharide), Ac was the control group (cells, culture medium containing CCK-8), and Ab was the blank group (no cells, culture medium containing CCK-8).

### 2.6. Data Analysis

All experiments were performed in triplicate, and the results were expressed as mean ± standard error (SEM). Analysis of significant differences in the results was performed using the one-way ANOVA analysis function in GraphPad Prism 10.4.0 (*p* < 0.05 means the difference is significant). The correlation analyses among TPC, TFC, DPPH, ABTS, and FRAP were performed using the Pearson correlation function in GraphPad Prism 10.4.0. The structures of solanine and soyasaponin I were drawn on the MolView website (v2.4, https://molview.org/, accessed on 25 June 2025).

## 3. Results

### 3.1. Physicochemical Properties of SH Samples

#### 3.1.1. Physicochemical Parameters of SH Samples

The physicochemical parameters of the SH samples are shown in Table 1. The water content, pH, glucose, fructose, sucrose, total phenols, and total flavonoid contents in the samples are significantly different.

#### 3.1.2. Chemical Composition of Methanol Extract of SH

**Figure 1:**
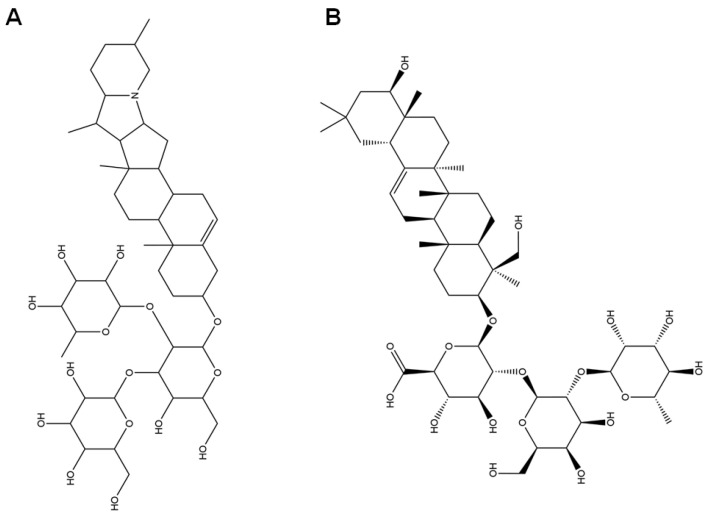
Structures of solanine (**A**) and soyasaponin I (**B**).

A total of 910 chemical components were detected in the methanol extract of SH by non-targeted metabolomics. The negative and positive ion spectra of the QC and methanol extract of the SH samples are presented in Figures S1–S8. There are two unique components of SH, solanine and soyasaponin I (Table 2 and Figure 1). There are 52 phenol, phenolic acid, and flavonoid components (Table 3). The other components are shown in Table S1.

### 3.2. Antioxidant Activity of SH Samples

**Figure 2:**
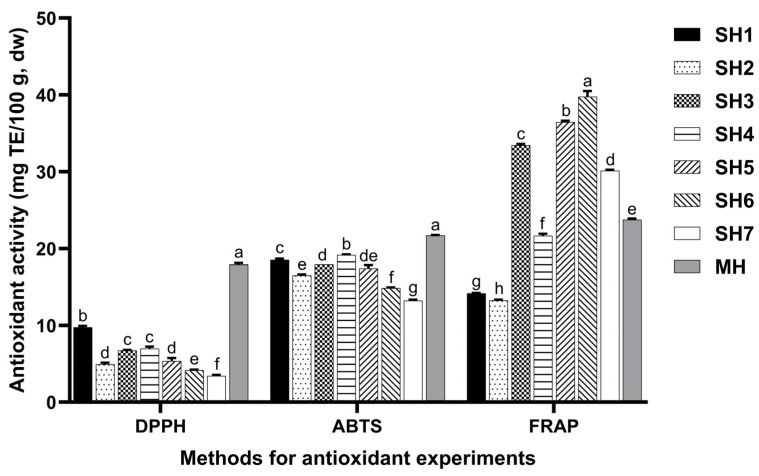
Antioxidant activity of SH samples. Different lowercase letters mean significant differences among antioxidant activity of SH samples determined by the same method (*p* < 0.05). MH, SH, DPPH, ABTS, and FRAP mean manuka honey (UMF 10+), *Schefflera oleifera* honey, DPPH free radical scavenging activity, ABTS cation radical scavenging activity, and FRAP total antioxidant capacity, respectively.

The antioxidant activity of the SH samples is shown in Figure 2. The correlations among TPC, TFC, DPPH, ABTS, and FRAP are presented in Table 4. The DPPH•, ABTS•+, and FRAP scavenging activities of these samples are significantly different.

### 3.3. Antibacterial Activity of SH Samples

As shown in Table 5, the MIC of SH samples against *E. coli* ranged from 30% to 40% and the MBC ranged from 30% to 40%; the MIC against *S. aureus* ranged from 30% to 40% and the MBC ranged from 35% to 45%; the MIC against *L. monocytogenes* ranged from 25% to 35% and the MBC ranged from 30% to 40%; and the MIC against *S. typhimurium* ranged from 15% to 20% and the MBC ranged from 20% to 30%. The antibacterial activity of SH is weaker than MH (UMF 10+).

### 3.4. Overlapping Targets of Main Components of SH and HepG2 Cells

**Figure 3:**
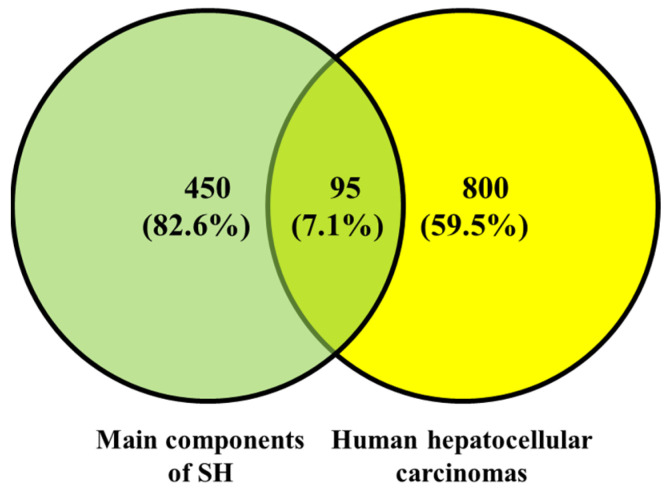
Overlapping targets between the main components of SH and targets of human hepatocellular carcinoma.

There were 545 targets of the main components of SH (phenols, flavonoids, saponins, triterpenes, and monoterpenes) collected through the SEA SearchEMBL-EBI. For HepG2, there were 895 targets. A Venn diagram of these targets was drawn (Figure 3). There were 95 overlapping targets between the main components of SH and HepG2: EGFR, AKT1, SRC, IL1B, ESR1, MMP9, TLR4, NFKB1, ALB, EP300, PIK3R1, RELA, PPARG, CXCL12, TLR2, PTGS2, PARP1, KDR, FGF2, PTPN11, MET, RHOA, PTPRC, IGF1R, CYP1A1, PTK2, MMP2, IL2, GSK3B, TNFRSF1A, SYK, IKBKG, CREB1, APP, PRKDC, CYP3A4, AR, RXRA, PPARA, LGALS3, IGFBP3, CYP1B1, CYP19A1, RARA, PLAU, PIK3CG, MMP3, ESR2, CYP1A2, INSR, IDO1, BRAF, NFE2L2, IGF2R, CYP17A1, COL18A1, ABL1, TOP2A, MPO, HSPD1, EPHX1, DPP4, CDK1, AHR, ABCG2, SLC2A1, PRNP, HNF4A, FASN, ERCC4, ERCC1, DNMT1, AXL, ARG1, ALK, TERT, RARB, NEK2, MMP13, CXCR1, COMT, ODC1, F2, CFTR, CA9, ABCC1, and ABCB1.

### 3.5. GO Functional Enrichment, KEGG Pathway Enrichment and PPI Analysis Results

**Figure 4:**
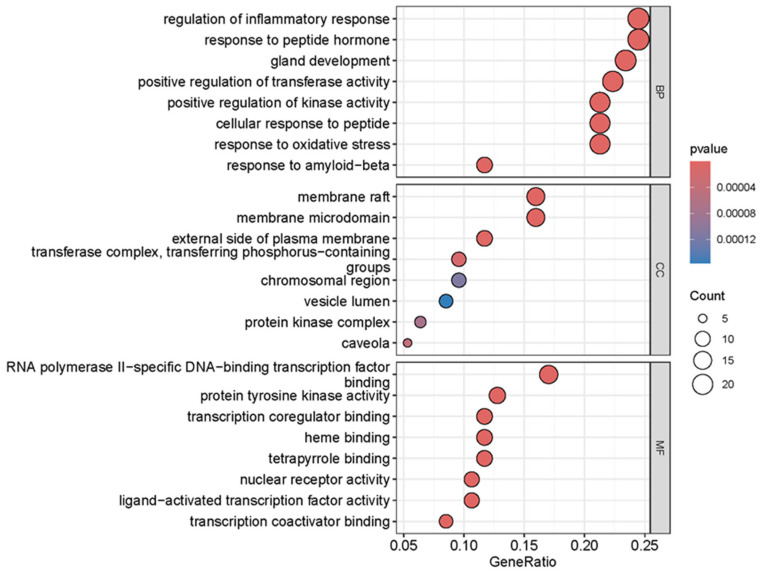
GO enrichment analysis of overlapping targets of main components of SH and HepG2 cells.

As shown in Figure 4, a total of 2304 relevant entries were screened out through GO enrichment analysis of the overlapping targets, of which 2035 entries were related to biological processes, mainly including regulation of inflammatory response, response to peptide hormone, positive regulation of kinase activity, gland development, positive regulation of transferase activity, response to amyloid-beta, cellular response to peptides, response to oxidative stress, negative regulation of cell adhesion and response to decreased oxygen levels. There were 71 entries related to cellular components, mainly including the membrane raft, membrane microdomain, external side of the plasma membrane, transferase complex, transferring phosphorus-containing groups, caveola, protein kinase complex, chromosomal region and le lumen. There were 198 items related to molecular functions, such as nuclear receptor activity, ligand-activated transcription factor activity, protein tyrosine kinase activity, transcription coregulator binding, RNA polymerase II-specific DNA-binding transcription factor binding, transcription coactivator binding, heme binding, tetrapyrrole binding, transmembrane receptor protein tyrosine kinase activity and DNA-binding transcription factor binding.

**Figure 5:**
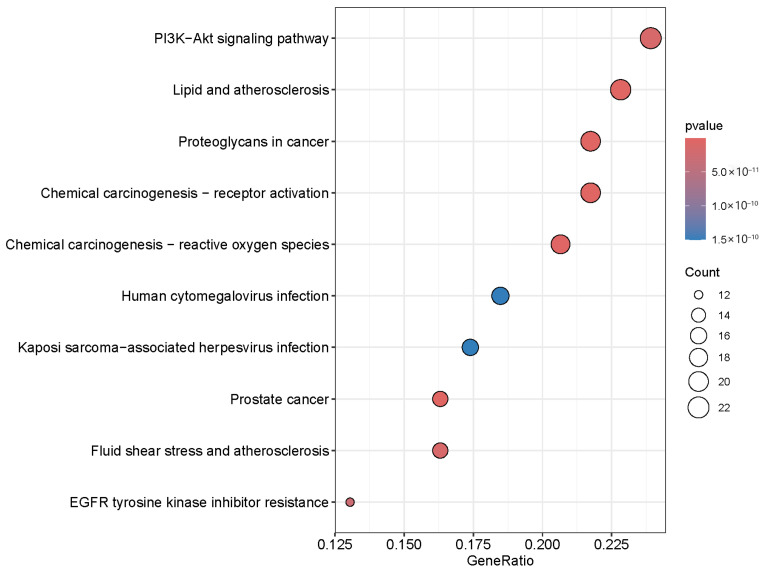
KEGG enrichment analysis of overlapping targets of the main components of SH and HepG2 cells.

The 95 overlapping targets between the main active ingredients of SH and HepG2 were enriched in the KEGG pathway (*p* < 0.05). The top 10 pathways were screened out (Figure 5), which were the PI3K-Akt pathway, Lipid and atherosclerosis, Proteoglycans in cancer, Chemical carcinogenesis—receptor activation, Chemical carcinogenesis—reactive oxygen species, Human cytomegalovirus infection, Kaposi sarcoma-associated herpesvirus infection, Prostate cancer, Fluid shear stress and atherosclerosis, and EGFR tyrosine kinase inhibitor resistance.

**Figure 6:**
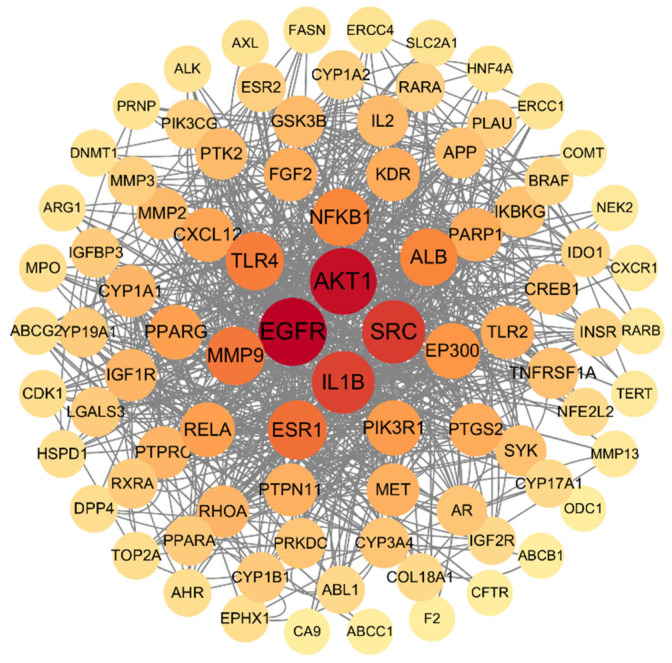
PPI analysis of common targets between main components of honey and human hepatocellular carcinoma. The circles represent proteins, straight lines represent the interaction relationship between proteins. The darker color means more interaction among proteins.

Among the overlapping targets between the main components of SH and HepG2, the protein–protein interactions with a confidence level of 0.7 are shown in Figure 6, among which the hub proteins are EGFR, AKT1, SRC, and IL1B. These traits represent the gene names of differentially expressed proteins, and the lines between genes represent the interactions of differentially expressed proteins. The darker the red and the larger the diameter mean the more related proteins among them.

### 3.6. Antiproliferative Effects of SH5 and Saccharides on HepG2 Cells

**Figure 7:**
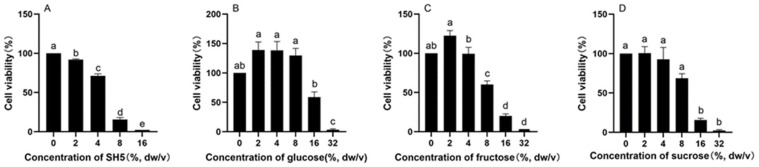
Inhibitory effects of SH5 (**A**), glucose (**B**), fructose (**C**) and sucrose (**D**) on the proliferation of HepG2 cells. The concentrations of glucose, fructose, and sucrose mean percent of stock solution prepared according to their contents of SH5. Different characters in the figure indicate significant differences among different treatments.

As shown in Figure 7, the inhibitory effects of SH5, glucose, fructose, and sucrose on the proliferation of HepG2 cells for 48 h were dose-dependent. The IC_50_ of SH, glucose, fructose, and sucrose on HepG2 cells was 5.07%, 16.24%, 9.60% dw/v, and 9.94% dw/v.

### 3.7. Contents of EGFR, SRC and AKT1 in Culture Medium of HepG2 Cells

**Figure 8:**
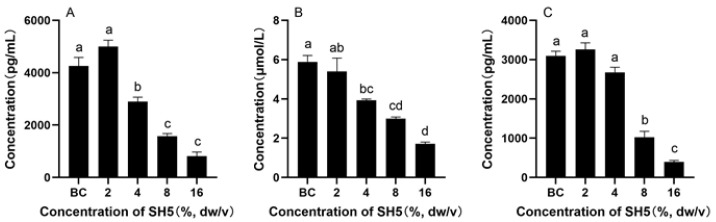
Protein content in HepG2 cell medium: (**A**) EGFR, (**B**) AKT1, and (**C**) SRC. Different lowercase characters in the figure indicate significant differences between different treatments.

As shown in Figure 8, the contents of EGFR, SRC, and AKT1 in the medium of HepG2 cells treated with SH were significantly lower than those of untreated cells.

## 4. Discussion

The compounds of SH determine its function. The moisture content, pH, glucose content, fructose content, sucrose content, total phenolic content, total flavonoid content, antioxidant activity, and antibacterial activity of the SH samples were significantly different. SH6 has the highest sucrose content (4.073 g/100 g) and also has the highest moisture content, which may indicate that the sample is not fully mature or there is a high humidity microenvironment around the beehive [59]. There were 910 ingredients in the methanol extract of SH identified using the UHPLC-MS/MS system, which includes phenols (such as phloroglucinol and pyrogallol), flavonoids (such as quercetin and kaempferol), triterpenes (such as betulin and limonin), saponins (such as soyasaponin I and solanine) and monoterpenoids. These components in SH contributed to its bioactivity.

The total phenolic and flavonoid compounds of SH are closely related to the antioxidant capacity. The antioxidant activity of SH7 is low, which is mostly related to its low total phenolic and flavonoid content. These results are consistent with reference [60,61], which states that the antioxidant capacity of honey is positively correlated with its polyphenol composition. Quercetin has antioxidant properties and can directly scavenge free radicals. It can also interact with ascorbic acid (vitamin C) to form a cycle: quercetin is oxidized after scavenging free radicals, and ascorbic acid can reduce it to restore its antioxidant capacity. This cycle is repeated, effectively maintaining the redox balance of the system and reducing the damage of free radicals to cells [62]. Quercetin can activate the Nrf2/Keap1 pathway, increase the level of antioxidant factors, reduce oxidative stress markers, inhibit the NF-κB pathway, and reduce the level of inflammatory mediators, thereby protecting against pulmonary ischemia–reperfusion injury [63]. 7-O-methylchrysin was identified as a potential marker of *Bauhinia championii* honey and was strongly positively correlated with free radical scavenging ability. In addition, the interaction between 7-O-methylchrysin and mineral elements such as K and Na may be one of the potential mechanisms for promoting the enhanced antioxidant capacity of *Bauhinia championii* honey [64].

Another important antioxidant component is soyasaponin I, a triterpenoid saponin, which can not only enhance immune regulation by mediating the p105-Tpl2-ERK signaling pathway but also reduce serum endotoxin, D-lactic acid, and oxidative stress levels, as well as alleviate intestinal pathological damage and inflammation [65]. Solanine, a component detected in the methanol extract of SH, opens up the permeability transition (PT) channels in the membrane by lowering the membrane potential, leading to Ca^2+^ being transported down its concentration gradient, which in turn leads to a rise in the concentration of Ca^2+^ in the cell, initiating the mechanism for apoptosis [66]. Saponins may play an antioxidant role depending on the synergistic enhancement with other phenols [67]. Saponins can synergize with phenolic compounds (such as flavonoids and phenolic acids) to enhance their antioxidant and antibacterial effects [68]. Based on our limited knowledge, solanine and soyasaponin I are the first determined components in honey.

The different antioxidant activities of different SH samples may be related to the different test methods and the antioxidant components in different honey samples. The DPPH method mainly detects hydrogen atom donors, while the FRAP method is more sensitive to electron donors [69]. SH3 and SH5 have high FRAP values, but their DPPH and ABTS activities are moderate, suggesting that the FRAP test is more sensitive to specific reducing components (such as ascorbic acid or certain flavonoid glycosides) [70]. In in vivo experiments, Ohia Lehua honey was shown to significantly enhance antioxidant capacity and reduce oxidative stress markers, with a significant increase in total antioxidant capacity (TAC) and a significant decrease in total oxidative state (TOS) and oxidative stress index (OSI). In contrast, the antioxidant activity of Manuka honey was limited and dose-dependent, with a significant increase in TAC and sulfhydryl contents and a moderate decrease in TOS and OSI [71]. Phenolic acids in multifloral honey can inhibit NADPH oxidase and reduce the generation of superoxide anions [72]. Antioxidant activity is one of the causes of antibacterial activity.

Antioxidant components in honey, such as polyphenols and flavonoids, also have an antibacterial effect by scavenging free radicals and reducing oxidative stress. Polyphenols inhibit the growth and reproduction of bacteria [73]. At the same time, they synergize with other antibacterial components in honey, such as hydroperoxides, to improve the overall antibacterial activity of honey [74]. In addition, the lowest pH value (2.907) of SH6 may be caused by the release of hydrogen peroxide, which inhibits bacterial enzyme activity [75]. Phloroglucinol and its derivatives exhibited anti-cancer effects in multiple pathways, including inducing cell apoptosis and inhibiting cell proliferation and migration [76,77]. In addition, phloroglucinol can also induce trained immunity by affecting metabolic and epigenetic pathways, thereby enhancing the body’s immune response to cancer cells [78]. In addition, saponins may regulate cell signaling pathways (such as PI3K-Akt and MAPK pathways) [79] to inhibit the proliferation and apoptosis of liver cancer cells.

SH, which has saponins, inhibits the proliferation of liver cancer cells. Cell proliferation of HepG2 cells was inhibited by SH in a dose-dependent manner (IC_50_ = 5.07% dw/v). Meanwhile, the IC_50_ value of SH’s saccharide components (glucose, fructose, sucrose) for HepG2 (9.60–16.24% dw/v) was significantly higher than that of SH, indicating that its anti-cancer effect mainly depends on the synergistic effect of sugars and non-sugar active ingredients (such as phenols and flavonoids) [80,81,82]. The mechanism of the antitumor effect against HepG2 cells depends on the complex components of SH.

The mechanism of the antitumor effect against HepG2 cells was analyzed by network pharmacology. There are 95 overlapping targets between the active ingredients of SH (total phenols, flavonoids, saponins, etc.) and HepG2 cells. The HepG2 cells were significantly inhibited after treatments of more than 4% (dw/v) SH. This result is in line with one study, which shows that honey can inhibit proliferation by scavenging free radicals and enhancing the total antioxidant state (TAS) [48]. SH has antioxidant activity and reduces the level of reactive oxygen in HepG2 cells; then, the proliferation is inhibited [83,84]. Long-term activation will disrupt the balance within the cell, leading to excessive cell stress response and inducing apoptosis [85,86]. The overlapping targets play an antitumor effect against HepG2 cells through pathways. KEGG enrichment analysis showed that the overlapping targets were mainly enriched in the following pathways: PI3K-Akt, Lipid and atherosclerosis, Proteoglycans in cancer, Chemical carcinogenesis—receptor activation, and Chemical carcinogenesis—reactive oxygen species. The PI3K/AKT/mTOR signaling pathway plays a key role in the occurrence and development of liver cancer. An increase in PI3K activity leads to phosphorylation of AKT, which in turn activates mTOR and promotes cell cycle progression and protein synthesis, thus promoting the development of liver cancer [87]. SH may induce HepG2 cell cycle arrest by inhibiting the phosphorylation of downstream effector molecules of the PI3K-Akt pathway (such as mTOR and BAD). TTFields treatment can activate the PI3K/AKT signaling pathway in cancer cells, and pharmacological inhibition of this pathway can enhance the therapeutic effect of TTFields. Combination therapy with PI3K inhibitors and TTFields can significantly reduce cell numbers, increase cell apoptosis, and reduce cloning ability. Inhibition of the PI3K-Akt pathway can significantly enhance the sensitivity of liver cancer cells to chemotherapy drugs [88]. SH provides a new strategy for combined therapy by targeting this pathway.

Another important pathway of the antitumor effect against HepG2 cells is Lipid and atherosclerosis. This pathway was the top pathway of the anti-atherosclerotic Tualang honey bioactive compound [89]. Proteoglycans in cancer is the top pathway when considering the different compounds and antiarrhythmic mechanisms of licorice before and after honey roasting [90]. It was also found in Jinfeng Pill, which is a Chinese medicine formula composed of nine kinds of herbs, which has been shown to alleviate damage to ovarian tissue induced by cyclophosphamide in a rat model of premature ovarian insufficiency [91]. Supplementation of honey for six months protects against DEN-induced inflammatory response and carcinogenesis in the liver of Sprague Dawley rats [92]. Honey and Nigella grains have a protective effect on methylnitrosourea-induced oxidative stress, inflammatory response, and carcinogenesis in Sprague Dawely rats [93]. SH inhibits the proliferation of HepG2 cells via multiple pathways, where DEPs play an inhibitory effect.

The PPI network analysis of DEPs in terms of SH’s antitumor effect against HepG2 cells confirmed that EGFR, AKT1, and SRC were the hub proteins. EGFR is one of the hub proteins that has an inhibition effect in terms of SH against HepG2 cells. EGF/EGFR can activate Akt/glycogen synthase kinase-3β (GSK-3 beta)/Snail pathway and mediate EMT to promote the proliferation and migration of HepG2 cells. Inhibiting the activation of the EGFR signaling pathway helps to partially reverse the EMT phenotype and inhibit the proliferation and migration of HepG2 cells [94]. Another hub protein is AKT1, of which down-regulation inhibited the proliferation of SMMC-7721 cells. In addition, the expression of AKT1 is closely related to cell apoptosis and cell cycle regulation. Knockdown of AKT1 significantly stimulated cell apoptosis and inhibited cell cycle progression [95]. Excessive phosphorylation of AKT1 leads to excessive activation of the mTOR pathway, thereby triggering autophagic cell death [66]. The third hub protein is SRC, which is highly expressed in a variety of cancers and activates the JAK/STAT pathway. It promotes the phosphorylation of STAT3, thereby enhancing the proliferation and survival of tumor cells [96]. The activation of SRC kinase may inhibit invasion and metastasis by destroying the stability of the cytoskeleton [97].

In conclusion, the down-regulation of key proteins such as EGFR, AKT1, and SRC was a possible mechanism of the antitumor effect of SH against HepG2. In the future, in-depth explorations of which bioactive components in SH have an anti-cancer effect and the specific effects of solanine and soybean saponin I can be conducted. In vivo experiments to verify the anti-cancer effect of SH in animals can be performed.

## 5. Conclusions

SH samples from different origins showed significant compositional diversity and weaker antibacterial activity than MH (UMF10+). The unique components solanine and soyasaponin I in SH may be used as characteristic substances for the identification of honey types and adulteration. The antitumor effect of SH on HepG2 cells was significantly stronger (IC_50_ = 5.07%) than that of carbohydrate components (IC_50_ = 9.60%~16.24%), of which the mechanism was seen in various pathways, PI3K-Akt, Lipid and atherosclerosis, Proteoglycans in cancer, Chemical carcinogenesis—receptor activation, and Chemical carcinogenesis—reactive oxygen species pathways, through network pharmacology analysis. The experimental results verified that the down-regulation of key proteins such as EGFR, AKT1, and SRC (*p* < 0.05) was a possible mechanism of the antitumor effect of SH against HepG2. This study expands the application boundaries of SH in the field of functional foods and food additives.

## Figures and Tables

**Table 1:** Physicochemical parameters of SH samples.

Samples	Moisture (%)	Glucose (g/100 g)	Fructose (g/100 g)	Sucrose (g/100 g)	pH	TPC (mgGAE/100 g)	TFC (mgRE/100 g)
SH1	22.781 ± 0.244 ^c^	30.855 ± 1.110 ^bc^	36.431 ± 1.013 ^c^	1.909 ± 0.061 ^c^	3.493 ± 0.015 ^b^	25.218 ± 0.143 ^d^	26.898 ± 0.281 ^d^
SH2	23.810 ± 0.172 ^b^	29.658 ± 0.427 ^c^	32.916 ± 0.509 ^d^	1.720 ± 0.051 ^c^	3.363 ± 0.007 ^c^	22.625 ± 0.174 ^e^	24.466 ± 0.562 ^e^
SH3	20.620 ± 0.306 ^d^	33.466 ± 0.372 ^ab^	42.351 ± 0.427 ^a^	2.901 ± 0.200 ^b^	3.503 ± 0.009 ^b^	50.519 ± 0.685 ^a^	32.087 ± 0.162 ^b^
SH4	25.034 ± 0.102 ^a^	34.997 ± 0.706 ^a^	41.111 ± 0.226 ^ab^	1.991 ± 0.097 ^c^	3.303 ± 0.009 ^d^	38.688 ± 1.531 ^bc^	32.087 ± 0.429 ^b^
SH5	22.313 ± 0.137 ^c^	27.189 ± 1.197 ^c^	39.777 ± 1.885 ^bc^	2.431 ± 0.059 ^b^	3.873 ± 0.009 ^a^	48.052 ± 0.793 ^a^	33.384 ± 0.162 ^a^
SH6	25.347 ± 0.250 ^a^	32.035 ± 0.388 ^b^	40.326 ± 0.441 ^b^	4.073 ± 0.230 ^a^	2.907 ± 0.012 ^e^	37.481 ± 0.813 ^c^	28.357 ± 0.281 ^c^
SH7	22.091 ± 0.136 ^c^	29.103 ± 1.463 ^c^	36.428 ± 1.989 ^cd^	1.350 ± 0.268 ^c^	2.917 ± 0.009 ^e^	42.848 ± 1.356 ^b^	28.033 ± 1.327 ^cd^

Different lowercase letters in the same column mean significantly different contents or values among different samples (*p* < 0.05). SH, TPC, and TFC mean *Schefflera oleifera* honey, the total phenolic content, and the total flavonoid content, respectively.

**Table 2:** The unique components determined in methanol extract of SH by non-targeted metabolomics.

No	Name	Formula	Molecular Weight (g/mol)	RT (min)	*m*/*z*	Relative Quantitative Value							**Polarity Mode**
						SH1	SH2	SH3	SH4	SH5	SH6	SH7	
1	Solanine	C_45_H_73_NO_15_	867.50084	11.029	868.50811	2.735 × 10^6^	5.854 × 10^6^	2.655 × 10^6^	1.144 × 10^6^	1.030 × 10^6^	1.047 × 10^6^	1.024 × 10^6^	Positive
2	Soyasaponin I	C_48_H_78_O_18_	942.52509	7.029	941.51781	4.818 × 10^5^	5.088 × 10^5^	4.686 × 10^6^	4.963 × 10^5^	3.209 × 10^6^	4.431 × 10^5^	4.895 × 10^5^	Negative

RT and *m*/*z* mean retention time AMD mass-to-charge ratio.

**Table 3:** Phenolics, phenolic acids and flavonoids determined in methanol extract of SH by non-targeted metabolomics (phenols: No 1–22, phenolic acids: No 23–27, flavonoids: No 28–52).

No	Name	Formula	Molecular Weight (g/mol)	RT (min)	*m*/*z*	Relative Quantitative Value							**Polarity Mode**
						SH1	SH2	SH3	SH4	SH5	SH6	SH7	
1	Phloroglucinol	C_6_H_6_O_3_	126.03196	0.674	127.03923	6.223 × 10^9^	6.923 × 10^9^	6.323 × 10^9^	7.156 × 10^9^	6.815 × 10^9^	6.504 × 10^9^	6.908 × 10^9^	Positive
2	Pyrogallol	C_6_H_6_O_3_	126.03198	4.963	127.03925	4.413 × 10^9^	4.927 × 10^9^	4.657 × 10^9^	5.204 × 10^9^	5.286 × 10^9^	4.346 × 10^9^	5.483 × 10^9^	Positive
3	2-Methoxyresorcinol	C_7_H_8_O_3_	140.04754	5.562	141.05477	3.667 × 10^8^	3.565 × 10^8^	2.644 × 10^8^	5.260 × 10^8^	5.261 × 10^8^	5.414 × 10^8^	3.133 × 10^8^	Positive
4	*o*-Cresol	C_7_H_8_O	108.05795	5.113	109.06522	2.422 × 10^8^	2.211 × 10^8^	2.544 × 10^8^	2.667 × 10^8^	3.388 × 10^8^	2.963 × 10^8^	2.947 × 10^8^	Positive
5	Epinephrine	C_9_H_13_NO_3_	183.08981	5.841	184.09709	1.019 × 10^8^	1.036 × 10^8^	5.186 × 10^8^	1.848 × 10^8^	4.545 × 10^8^	1.538 × 10^8^	2.471 × 10^8^	Positive
6	Homovanillic acid	C_9_H_10_O_4_	182.05809	1.399	183.06537	2.510 × 10^8^	2.674 × 10^8^	2.439 × 10^8^	2.526 × 10^8^	2.406 × 10^8^	2.166 × 10^8^	2.326 × 10^8^	Positive
7	Isoproterenol	C_11_H_17_NO_3_	211.12114	5.409	212.12842	1.054 × 10^8^	1.027 × 10^8^	4.461 × 10^8^	1.899 × 10^8^	3.098 × 10^8^	1.990 × 10^8^	3.028 × 10^8^	Positive
8	4-Butylresorcinol	C_10_H_14_O_2_	166.09949	5.886	165.09221	1.154 × 10^8^	1.242 × 10^8^	1.932 × 10^8^	1.319 × 10^8^	3.196 × 10^8^	1.366 × 10^8^	1.122 × 10^8^	Negative
9	Isohomovanillic acid	C_9_H_10_O_4_	182.05823	0.734	183.06554	1.354 × 10^8^	1.292 × 10^8^	1.000 × 10^8^	9.865 × 10^7^	8.626 × 10^7^	7.681 × 10^7^	7.497 × 10^7^	Positive
10	Vanillyl alcohol	C_8_H_10_O_3_	154.0631	5.127	153.05582	1.054 × 10^8^	9.165 × 10^7^	9.359 × 10^7^	1.150 × 10^8^	8.903 × 10^7^	6.270 × 10^7^	5.799 × 10^7^	Negative
11	Coniferyl alcohol	C_10_H_12_O_3_	180.08294	5.384	181.09022	8.871 × 10^7^	9.895 × 10^7^	8.129 × 10^7^	8.962 × 10^7^	8.275 × 10^7^	7.915 × 10^7^	8.248 × 10^7^	Positive
12	Norepinephrine	C_8_H_11_NO_3_	169.07415	5.715	168.0668	2.254 × 10^7^	2.365 × 10^7^	1.190 × 10^8^	4.120 × 10^7^	1.497 × 10^8^	1.153 × 10^8^	6.164 × 10^7^	Negative
13	Paracetamol	C_8_H_9_NO_2_	151.06354	5.469	152.07081	2.791 × 10^7^	3.312 × 10^7^	8.364 × 10^7^	3.013 × 10^7^	2.332 × 10^8^	3.930 × 10^7^	4.806 × 10^7^	Positive
14	Hydroquinone	C_6_H_6_O_2_	110.03692	5.43	109.02964	4.622 × 10^7^	3.999 × 10^7^	6.705 × 10^7^	4.566 × 10^7^	1.220 × 10^8^	3.691 × 10^7^	3.182 × 10^7^	Negative
15	3-Methoxytyramine	C_9_H_13_NO_2_	167.09475	5.887	166.08748	1.071 × 10^7^	1.011 × 10^7^	1.016 × 10^8^	2.205 × 10^7^	2.175 × 10^7^	6.433 × 10^7^	8.361 × 10^7^	Negative
16	Metanephrine	C_10_H_15_NO_3_	197.1055	5.566	196.09821	1.417 × 10^7^	1.911 × 10^7^	5.605 × 10^7^	3.287 × 10^7^	5.856 × 10^7^	4.625 × 10^7^	4.229 × 10^7^	Negative
17	Tyrosol	C_8_H_10_O_2_	138.06822	5.607	137.06092	2.602 × 10^7^	3.210 × 10^7^	3.734 × 10^7^	4.127 × 10^7^	3.726 × 10^7^	2.778 × 10^7^	3.089 × 10^7^	Negative
18	*L*-Adrenaline	C_9_H_13_NO_3_	183.08975	5.609	184.09699	1.080 × 10^7^	1.013 × 10^7^	5.898 × 10^7^	1.587 × 10^7^	3.052 × 10^7^	3.194 × 10^7^	5.137 × 10^7^	Positive
19	Terbutaline	C_12_H_19_NO_3_	225.13669	5.106	226.14393	7.840 × 10^6^	5.561 × 10^6^	2.662 × 10^7^	1.275 × 10^7^	1.882 × 10^7^	1.446 × 10^7^	1.729 × 10^7^	Positive
20	2,4-Dinitrophenol	C_6_H_4_N_2_O_5_	184.01209	5.956	183.00481	8.349 × 10^6^	8.576 × 10^6^	1.171 × 10^7^	7.434 × 10^6^	5.036 × 10^7^	5.230 × 10^6^	4.898 × 10^6^	Negative
21	Isorhapontigenin	C_15_H_14_O_4_	258.08946	6.285	257.08218	8.344 × 10^6^	9.100 × 10^6^	4.291 × 10^6^	3.304 × 10^6^	6.511 × 10^6^	4.028 × 10^6^	9.592 × 10^5^	Negative
22	Epinephrine bitartrate	C_13_H_19_NO_9_	333.10563	5.016	334.1129	4.232 × 10^6^	3.074 × 10^6^	1.264 × 10^6^	2.484 × 10^6^	2.104 × 10^6^	1.537 × 10^6^	9.312 × 10^5^	Positive
23	2,3,4-Trihydroxybenzoic acid	C_7_H_6_O_5_	170.02174	4.867	169.01446	3.033 × 10^7^	3.680 × 10^7^	2.684 × 10^7^	7.454 × 10^7^	1.400 × 10^7^	8.434 × 10^6^	8.492 × 10^6^	Negative
24	4-Methoxycinnamic Acid	C_10_H_10_O_3_	178.0631	5.586	177.05604	1.043 × 10^7^	2.076 × 10^7^	4.066 × 10^7^	2.778 × 10^7^	3.350 × 10^7^	1.823 × 10^7^	2.030 × 10^7^	Negative
25	*P*-Coumaroyl Agmatine	C_14_H_20_N_4_O_2_	276.15771	5.551	275.15022	1.520 × 10^7^	2.845 × 10^7^	2.085 × 10^7^	2.719 × 10^7^	2.105 × 10^7^	1.878 × 10^7^	2.266 × 10^7^	Negative
26	Feruloyl Putrescine	C_14_H_20_N_2_O_3_	264.14758	5.389	263.14041	3.641 × 10^6^	4.344 × 10^6^	2.128 × 10^6^	4.742 × 10^6^	1.090 × 10^6^	1.961 × 10^6^	3.326 × 10^6^	Negative
27	Methyl cinnamate	C_10_H_10_O_2_	162.06565	5.475	163.07256	6.174 × 10^5^	1.127 × 10^6^	6.217 × 10^5^	6.326 × 10^5^	6.490 × 10^5^	6.394 × 10^5^	5.806 × 10^5^	Positive
28	Quercetin	C_15_H_10_O_7_	302.04295	5.874	301.03567	6.200 × 10^8^	5.706 × 10^8^	8.050 × 10^8^	1.155 × 10^9^	2.115 × 10^8^	3.015 × 10^8^	3.613 × 10^8^	Negative
29	Luteolin	C_15_H_10_O_6_	286.048	6.079	285.04073	3.057 × 10^8^	3.012 × 10^8^	3.358 × 10^8^	5.713 × 10^8^	8.111 × 10^7^	1.087 × 10^8^	1.305 × 10^8^	Negative
30	Naringenin	C_15_H_12_O_5_	272.06864	6.042	271.06137	4.198 × 10^7^	4.899 × 10^7^	5.329 × 10^8^	7.603 × 10^7^	4.169 × 10^8^	3.740 × 10^7^	8.208 × 10^7^	Negative
31	Rutin	C_27_H_30_O_16_	610.1547	5.507	609.14742	1.144 × 10^8^	1.402 × 10^8^	2.518 × 10^8^	3.715 × 10^8^	3.352 × 10^7^	7.991 × 10^7^	3.244 × 10^7^	Negative
32	Apigenin	C_15_H_10_O_5_	270.0532	6.094	315.05145	2.279 × 10^8^	1.805 × 10^8^	1.571 × 10^8^	1.230 × 10^8^	1.072 × 10^8^	7.363 × 10^7^	9.890 × 10^7^	Negative
33	Quercetin-3β-D-glucoside	C_21_H_20_O_12_	464.09629	5.556	463.08902	7.742 × 10^7^	7.681 × 10^7^	7.941 × 10^7^	1.492 × 10^8^	2.627 × 10^7^	1.678 × 10^8^	1.455 × 10^8^	Negative
34	Chrysin	C_15_H_10_O_4_	254.05801	6.684	253.05073	7.896 × 10^6^	6.067 × 10^6^	2.637 × 10^8^	1.848 × 10^7^	2.410 × 10^8^	4.648 × 10^6^	1.261 × 10^7^	Negative
35	Myricetin	C_15_H_10_O_8_	318.03798	5.68	317.0307	5.480 × 10^7^	4.625 × 10^7^	2.184 × 10^8^	1.454 × 10^8^	1.251 × 10^7^	8.715 × 10^6^	9.458 × 10^6^	Negative
36	Genistein	C_15_H_10_O_5_	270.05296	6.759	269.04569	7.687 × 10^6^	6.540 × 10^6^	1.148 × 10^8^	1.689 × 10^7^	1.014 × 10^8^	7.427 × 10^6^	5.671 × 10^6^	Negative
37	Glycitein	C_16_H_12_O_5_	284.06854	6.852	283.06127	1.539 × 10^6^	1.068 × 10^6^	6.000 × 10^7^	3.257 × 10^6^	5.368 × 10^7^	8.556 × 10^5^	2.424 × 10^6^	Negative
38	Kaempferol	C_15_H_10_O_6_	286.04777	5.774	287.05504	7.797 × 10^6^	9.314 × 10^6^	1.303 × 10^7^	1.679 × 10^7^	9.587 × 10^6^	3.452 × 10^7^	2.510 × 10^7^	Positive
39	Equol	C_15_H_14_O_3_	242.09053	4.919	241.08325	1.268 × 10^7^	1.296 × 10^7^	1.512 × 10^7^	1.721 × 10^7^	2.031 × 10^7^	9.258 × 10^6^	1.560 × 10^7^	Negative
40	Epigallocatechin	C_15_H_14_O_7_	306.07613	2.263	307.08341	1.566 × 10^7^	1.856 × 10^7^	8.956 × 10^5^	2.430 × 10^7^	5.053 × 10^6^	1.497 × 10^6^	6.615 × 10^5^	Positive
41	Daidzein	C_15_H_10_O_4_	254.05797	5.854	253.0507	2.915 × 10^6^	2.347 × 10^6^	3.708 × 10^7^	1.210 × 10^6^	1.993 × 10^7^	9.042 × 10^5^	9.940 × 10^5^	Negative
42	Quercetin-3-O-beta-glucopyranosyl-6′-acetate	C_23_H_22_O_13_	506.10678	5.56	505.0995	1.084 × 10^6^	1.083 × 10^6^	6.628 × 10^6^	1.312 × 10^6^	4.737 × 10^7^	1.224 × 10^6^	1.129 × 10^6^	Negative
43	Isorhamnetin	C_16_H_12_O_7_	316.05803	6.101	317.0653	1.301 × 10^7^	1.151 × 10^7^	8.612 × 10^6^	9.060 × 10^6^	5.996 × 10^6^	4.275 × 10^6^	6.463 × 10^6^	Positive
44	Diosmetin	C_16_H_12_O_6_	300.06356	5.49	301.07084	1.236 × 10^7^	1.337 × 10^7^	1.501 × 10^6^	5.076 × 10^6^	1.275 × 10^6^	2.941 × 10^6^	1.089 × 10^6^	Positive
45	Flavanone	C_15_H_12_O_2_	224.08409	6.713	223.07681	4.440 × 10^5^	4.822 × 10^5^	2.098 × 10^7^	6.869 × 10^5^	1.201 × 10^7^	4.162 × 10^5^	9.074 × 10^5^	Negative
46	Taxifolin	C_15_H_12_O_7_	304.05605	5.038	305.06332	4.749 × 10^6^	6.574 × 10^6^	6.850 × 10^6^	7.480 × 10^6^	7.081 × 10^5^	4.857 × 10^5^	5.535 × 10^5^	Positive
47	Poncirin	C_28_H_34_O_14_	594.19385	5.064	595.20113	2.307 × 10^6^	2.433 × 10^6^	1.682 × 10^7^	2.873 × 10^6^	5.325 × 10^5^	5.556 × 10^5^	1.260 × 10^6^	Positive
48	Pinocembrin	C_15_H_12_O_4_	256.07366	6.528	257.08094	6.677 × 10^5^	6.933 × 10^5^	1.074 × 10^7^	8.733 × 10^5^	1.039 × 10^7^	5.976 × 10^5^	5.864 × 10^5^	Positive
49	Galangin	C_15_H_10_O_5_	116.05032	6.319	117.05763	1.031 × 10^6^	1.026 × 10^6^	5.397 × 10^6^	1.909 × 10^6^	9.752 × 10^6^	3.038 × 10^6^	9.401 × 10^5^	Positive
50	Kuromanin	C_21_H_20_O_11_	426.12033	9.635	449.10965	2.724 × 10^6^	2.242 × 10^6^	2.745 × 10^6^	2.464 × 10^6^	1.876 × 10^6^	2.363 × 10^6^	2.024 × 10^6^	Positive
51	Tangeritin	C_20_H_20_O_7_	372.12089	6.841	373.12823	7.277 × 10^5^	8.114 × 10^5^	5.987 × 10^6^	8.897 × 10^5^	6.720 × 10^5^	7.501 × 10^5^	6.686 × 10^5^	Positive
52	Nobiletin	C_21_H_22_O_8_	402.13185	6.532	403.13926	5.947 × 10^5^	6.175 × 10^5^	2.790 × 10^6^	8.281 × 10^5^	5.223 × 10^5^	6.614 × 10^5^	5.223 × 10^5^	Positive

RT and *m*/*z* mean retention time AMD mass-to-charge ratio.

**Table 4:** The correlations among TPC, TFC, DPPH, ABTS, and FRAP.

	TPC	TFC	DPPH	ABTS	FRAP
TPC	1				
TFC	0.849637	1			
DPPH	−0.29206	0.07948	1		
ABTS	−0.09067	0.381084	0.828684	1	
FRAP	0.813372	0.587983	−0.52454	−0.39716	1

TPC, TFC, DPPH, ABTS, and FRAP mean the total phenolic content, the total flavonoid content, DPPH free radical scavenging activity, ABTS cation radical scavenging activity, and FRAP total antioxidant capacity, respectively.

**Table 5:** Antibacterial activity of SH samples against four foodborne pathogens.

Samples	*E. coli* (%)		*S. aureus* (%)		*L. monocytogenes* (%)		*S. typhimurium* (%)	
	MIC	MBC	MIC	MBC	MIC	MBC	MIC	MBC
SH1	30	30	35	45	30	35	15	20
SH2	30	35	35	40	35	40	15	20
SH3	35	40	40	40	30	30	20	30
SH4	30	30	30	35	25	30	20	25
SH5	30	30	30	35	25	30	20	20
SH6	40	40	35	40	35	40	20	25
SH7	30	40	40	45	25	30	15	25
MH (UMF 10+)	20	30	15	15	20	25	10	15

## Data Availability

The original contributions presented in this study are included in the article/Supplementary Materials. Further inquiries can be directed to the corresponding author.

## Supplementary Materials

The following supporting information can be downloaded at: https://www.mdpi.com/article/10.3390/foods14132376/s1, Figures S1–S8: The negative and positive ion spectra of QC and methanol extract of SH samples; Table S1: Components except phenolics, phenolic acids, flavonoids, solanine and soyasaponin I determined in methanol extract of SH by non-targeted metabolomics.
