# Supplementary material for: Physicochemical Properties, Antioxidant and Antibacterial Activities and Anti-Hepatocarcinogenic Effect and Potential Mechanism of Schefflera oleifera Honey Against HepG2 Cells
Source: Foods. 2025 Jul 4;14(13):2376. doi: 10.3390/foods14132376 (PMC12249035; doi:10.3390/foods14132376)
Supplement: Supplementary file 1 [file foods-14-02376-s001.zip › foods-3704878-supplementary.pdf]

foods

MDPI

Article

The Physicochemical Properties, Antioxidant and Antibacterial Activities and Multi-Target Anti-Hepatocarcinogenic Mechanism of *Schefflera Oleifera* Honey

Jingjing Li [1], Jie Wang [1], Yicong Wang [1] and Wenchao Yang [1*]

[1] College of Bee Science and Biomedicine, Fujian Agriculture and Forestry University, Fuzhou 350002, China; lijingjing000407@163.com (J.L.); wangjie01092023@163.com (J.W.); 18265526098@163.com (Y.W.)

* Correspondence: beesyang@gmail.com

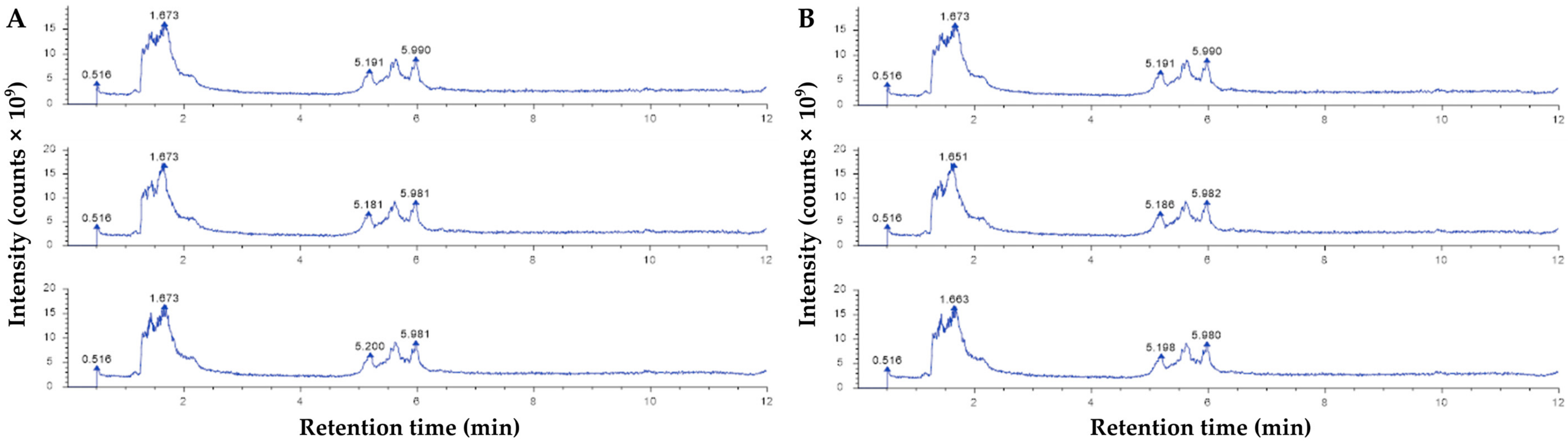

Figure S1. QC spectra of (A) negative and (B) positive ions.

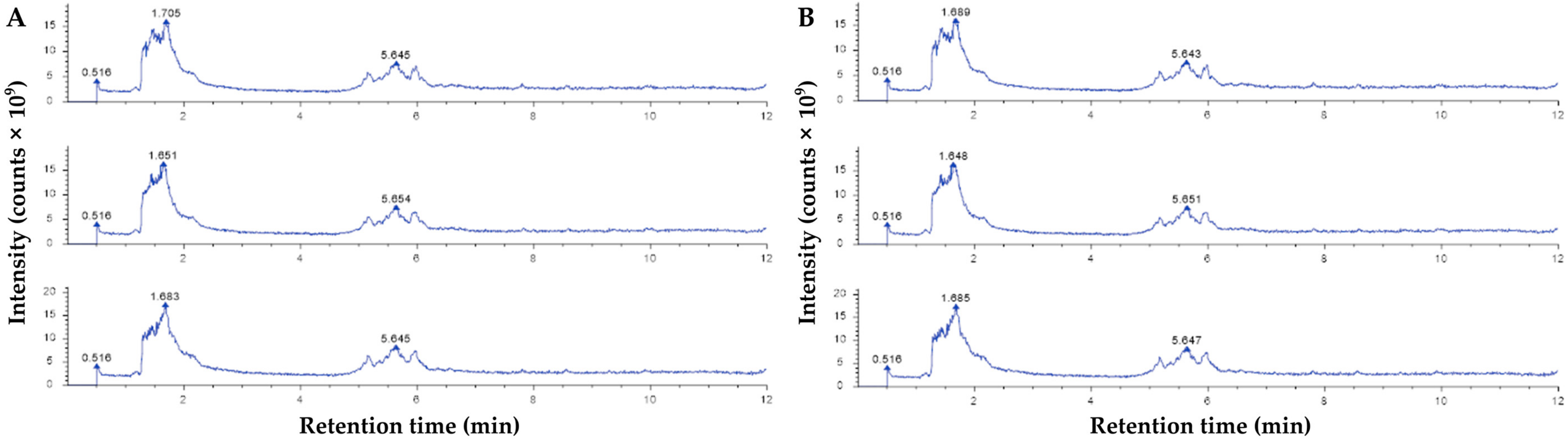

Figure S2. The (A) negative and (B) positive ion spectra of SH1 methanol extract.

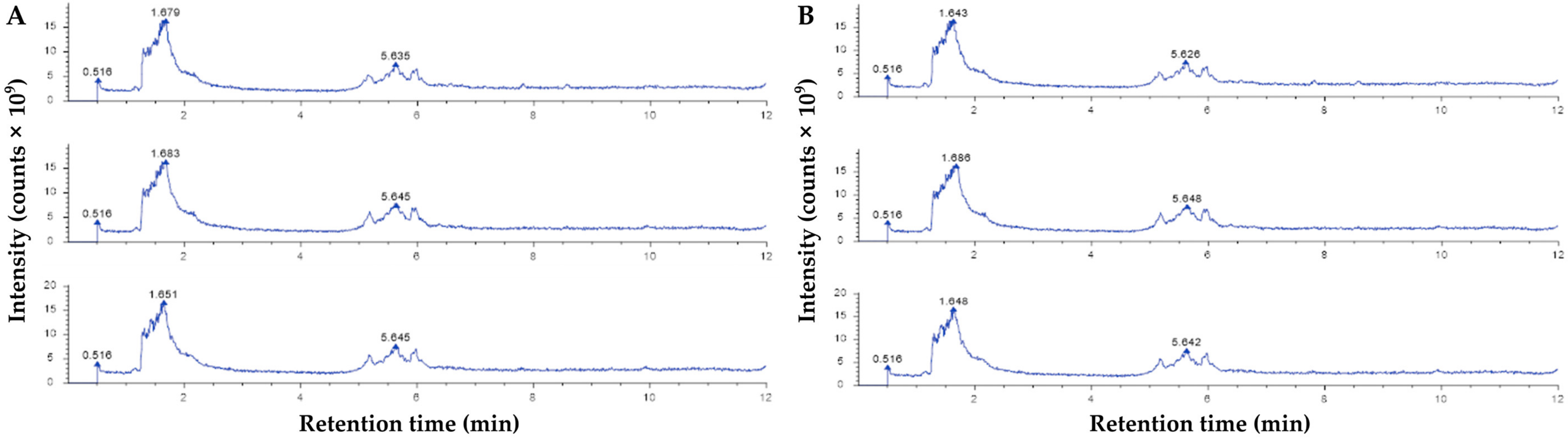

Figure S3. The (A) negative and (B) positive ion spectra of SH2 methanol extract.

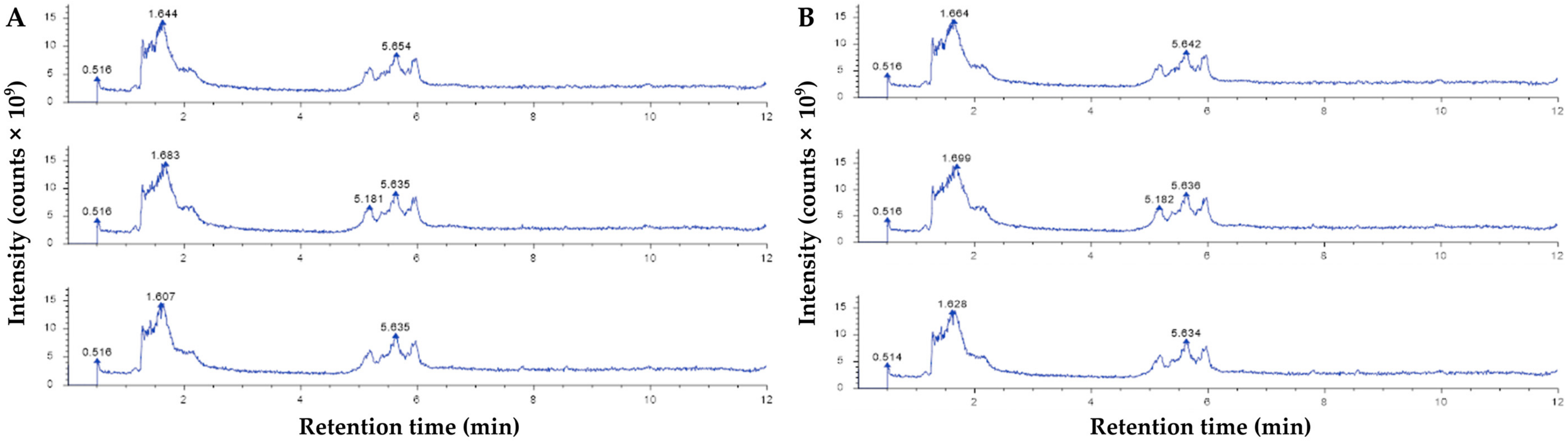

Figure S4. The (A) negative and (B) positive ion spectra of SH3 methanol extract.

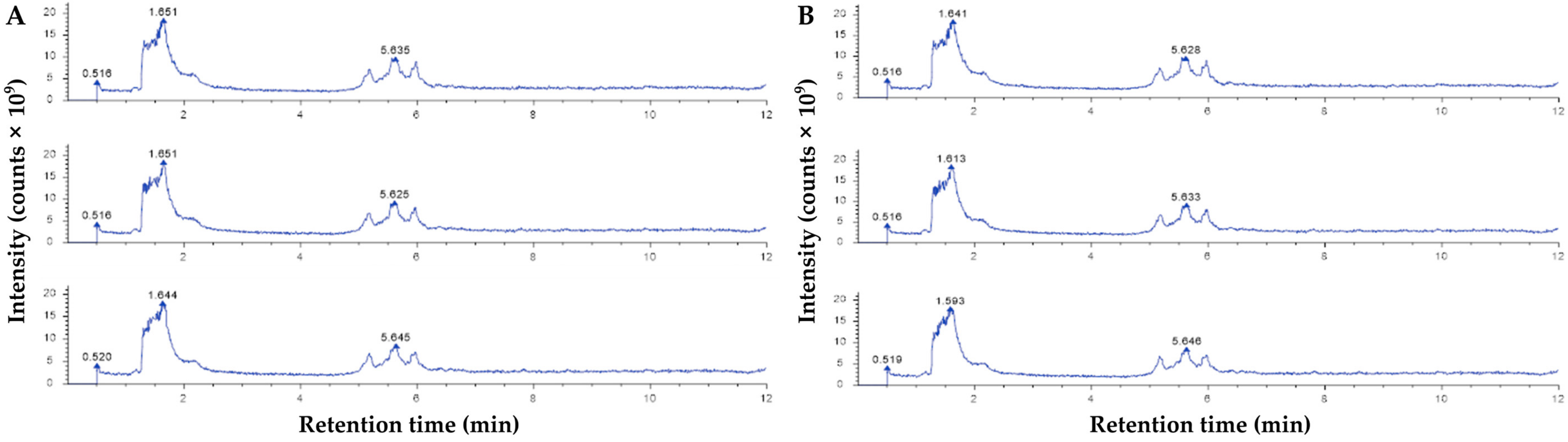

Figure S5. The (A) negative and (B) positive ion spectra of SH4 methanol extract.

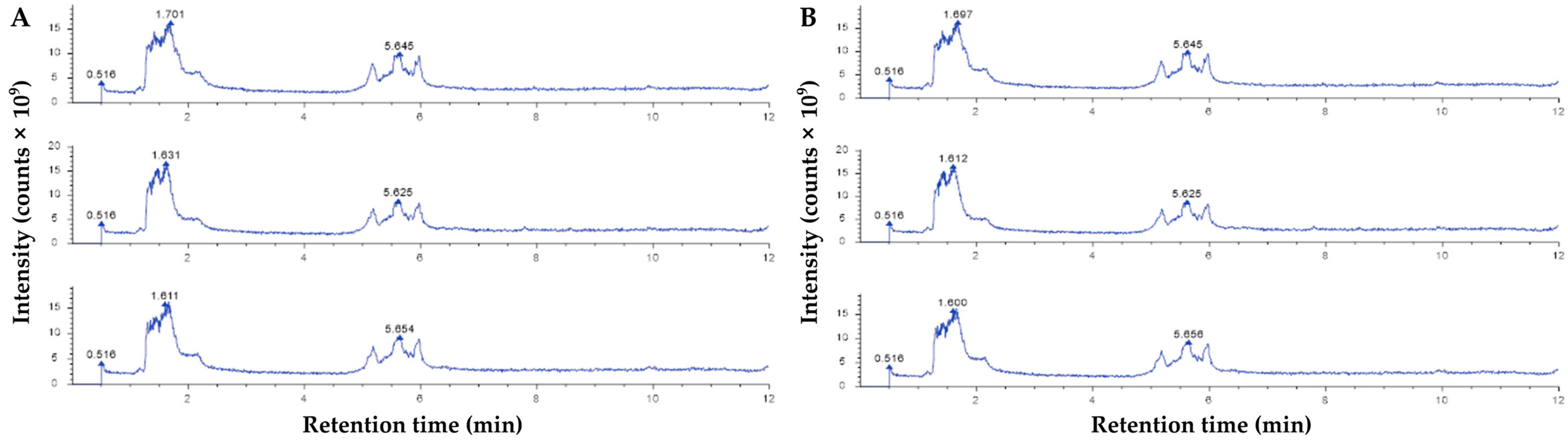

Figure S6. The (A) negative and (B) positive ion spectra of SH5 methanol extract.

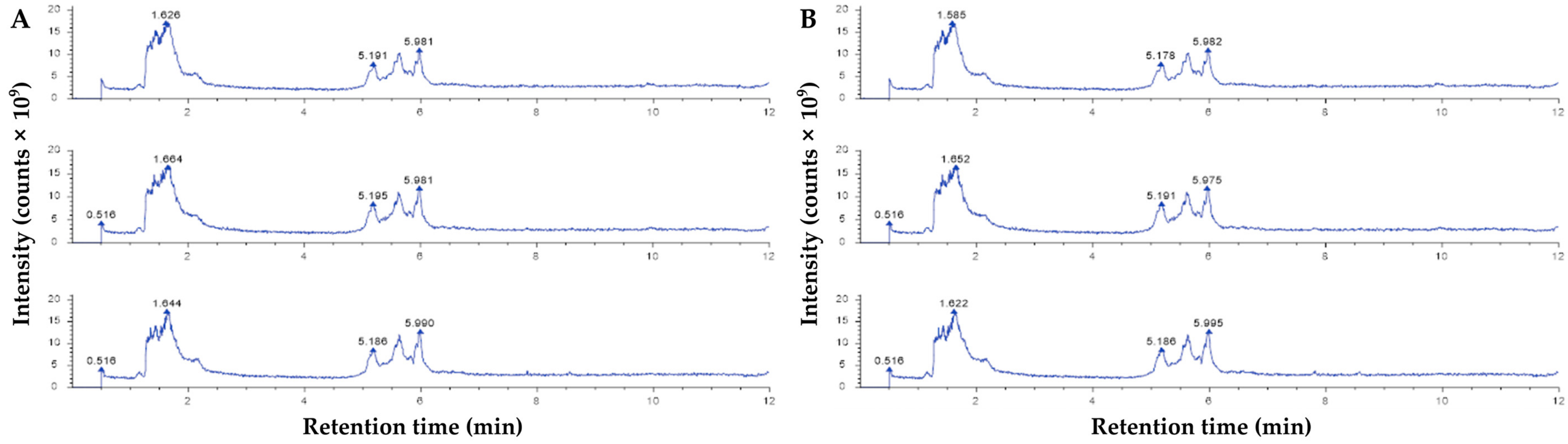

Figure S7. The (A) negative and (B) positive ion spectra of SH6 methanol extract.

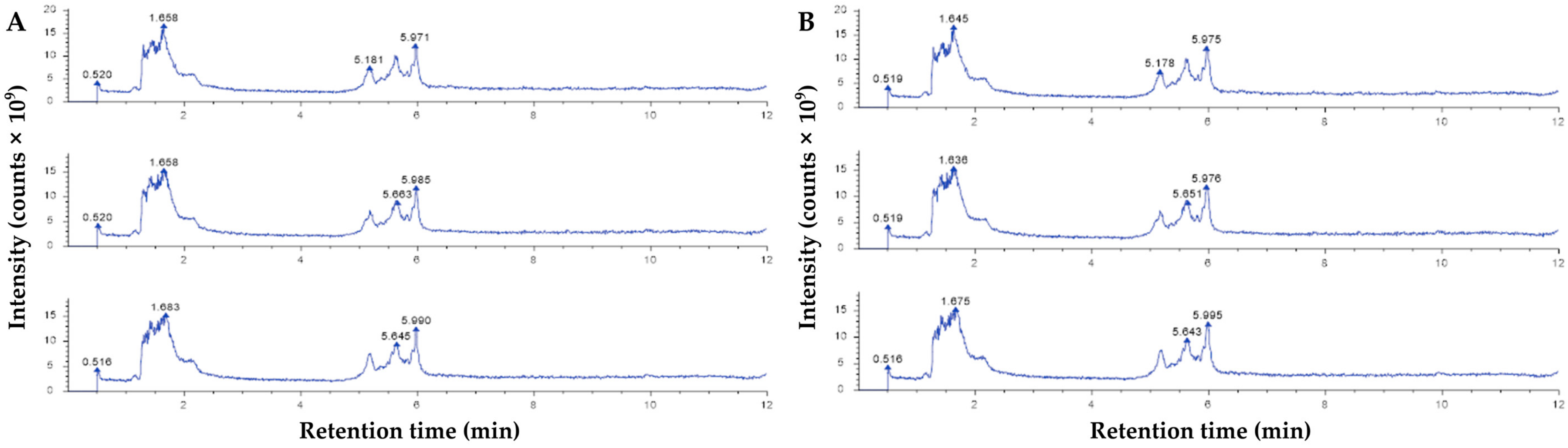

Figure S8. The (A) negative and (B) positive ion spectra of SH7 methanol extract.

Table S1. Components except phenolics, phenolic acids, flavonoids, solanine and soyasaponin I determined in methanol extract of SH by non-targeted metabolomics.

No	Name	Formula	Molecular weight (g/mol)	RT (min)	m/z	Relative quantitative value							Polarity mode
						SH1	SH2	SH3	SH4	SH5	SH6	SH7	
1	*D*-(+)-Glucose	$C_6H_{12}O_6$	226.06884	1.689	225.06157	1.142×10^{11}	1.441×10^{11}	1.536×10^{11}	1.090×10^{11}	1.244×10^{11}	1.264×10^{11}	1.419×10^{11}	Negative
2	*D*-(+)-Mannose	$C_6H_{12}O_6$	180.06342	1.712	179.05621	7.050×10^{10}	8.759×10^{10}	9.163×10^{10}	6.071×10^{10}	7.044×10^{10}	7.078×10^{10}	8.759×10^{10}	Negative
3	Oleamide	$C_{18}H_{35}NO$	281.27159	9.922	304.26072	7.166×10^{10}	7.115×10^{10}	5.363×10^{10}	6.294×10^{10}	6.730×10^{10}	5.946×10^{10}	5.908×10^{10}	Positive
4	Gluconic acid	$C_6H_{12}O_7$	196.05816	1.603	195.05089	5.229×10^{10}	5.483×10^{10}	3.867×10^{10}	4.592×10^{10}	4.458×10^{10}	5.331×10^{10}	4.621×10^{10}	Negative
5	Citric acid	$C_6H_8O_7$	192.02712	1.763	191.01985	3.160×10^{10}	2.908×10^{10}	3.718×10^{10}	3.599×10^{10}	3.442×10^{10}	3.432×10^{10}	3.925×10^{10}	Negative
6	Citral	$C_{10}H_{16}O$	152.12024	5.602	153.1275	1.640×10^{10}	1.660×10^{10}	1.885×10^{10}	1.951×10^{10}	1.845×10^{10}	2.033×10^{10}	1.853×10^{10}	Positive
7	3-hydroxy-3-methylpentanedioic acid	$C_6H_{10}O_5$	179.07955	1.564	180.08683	1.869×10^{10}	1.966×10^{10}	1.728×10^{10}	1.835×10^{10}	1.694×10^{10}	1.679×10^{10}	1.724×10^{10}	Positive
8	5-hydroxy-4-methoxy-5,6-dihydro-2H-pyran-2-one	$C_6H_8O_4$	144.04241	0.691	145.04967	1.644×10^{10}	1.762×10^{10}	1.573×10^{10}	1.751×10^{10}	1.630×10^{10}	1.487×10^{10}	1.597×10^{10}	Positive
9	*D*-(+)-Malic acid	$C_4H_6O_5$	134.02154	1.67	133.01427	2.566×10^{10}	2.718×10^{10}	2.999×10^{9}	3.182×10^{10}	2.693×10^{9}	5.043×10^{9}	2.128×10^{9}	Negative
10	Hexadecanamide	$C_{16}H_{33}NO$	255.25614	9.711	256.26342	1.157×10^{10}	1.266×10^{10}	1.144×10^{10}	9.249×10^{9}	1.143×10^{10}	1.062×10^{10}	1.055×10^{10}	Positive
11	Oleoyl ethylamide	$C_{20}H_{39}NO$	309.30302	10.763	332.29221	1.141×10^{10}	1.220×10^{10}	1.052×10^{10}	1.003×10^{10}	1.100×10^{10}	9.639×10^{9}	1.086×10^{10}	Positive
12	*D*-(+)-Maltose	$C_{12}H_{22}O_{11}$	388.12196	1.656	387.11468	9.336×10^{9}	8.995×10^{9}	8.793×10^{9}	5.576×10^{9}	1.011×10^{10}	1.272×10^{10}	8.965×10^{9}	Negative

13	*trans*-2-Butene-1,4-dicarboxylic Acid	$C_6H_8O_4$	144.04231	1.701	143.03503	7.305×10^9	1.128×10^{10}	1.069×10^{10}	6.319×10^9	6.919×10^9	7.719×10^9	6.515×10^9	Negative
14	1-Palmitoylglycerol	$C_{19}H_{38}O_4$	330.27681	9.912	353.26604	8.052×10^9	8.718×10^9	9.489×10^9	8.124×10^9	8.011×10^9	6.783×10^9	6.084×10^9	Positive
15	Phloroglucinol	$C_6H_6O_3$	126.03196	0.674	127.03923	6.223×10^9	6.923×10^9	6.323×10^9	7.156×10^9	6.815×10^9	6.504×10^9	6.908×10^9	Positive
16	1-Stearoylglycerol	$C_{21}H_{42}O_4$	358.30817	10.754	381.29738	7.324×10^9	7.643×10^9	7.005×10^9	5.543×10^9	6.489×10^9	6.039×10^9	5.381×10^9	Positive
17	Jasmonic acid	$C_{12}H_{18}O_3$	210.12336	5.601	211.13063	5.355×10^9	5.499×10^9	5.880×10^9	6.334×10^9	6.227×10^9	6.460×10^9	6.131×10^9	Positive
18	Methylmalonic acid	$C_4H_6O_4$	118.02668	2.774	117.0194	7.814×10^9	6.941×10^9	4.208×10^9	6.342×10^9	3.607×10^9	4.054×10^9	4.182×10^9	Negative
19	Stearamide	$C_{18}H_{37}NO$	283.2874	10.575	306.27655	4.969×10^9	6.090×10^9	4.801×10^9	4.076×10^9	4.626×10^9	4.752×10^9	5.865×10^9	Positive
20	Pyrogallol	$C_6H_6O_3$	126.03198	4.963	127.03925	4.413×10^9	4.927×10^9	4.657×10^9	5.204×10^9	5.286×10^9	4.346×10^9	5.483×10^9	Positive
21	2-(methylthio)-4-(3-pyridyl)pyrimidine	$C_{10}H_9N_3S$	203.04866	10.918	204.05591	4.452×10^9	5.061×10^9	4.638×10^9	5.133×10^9	4.796×10^9	4.508×10^9	4.702×10^9	Positive
22	4-oxododecanedioic acid	$C_{12}H_{20}O_5$	226.11813	5.862	227.12541	3.618×10^9	3.804×10^9	8.110×10^9	3.107×10^9	4.697×10^9	6.304×10^9	3.089×10^9	Positive
23	8-Hydroxyquinoline	C_9H_7NO	145.05289	5.354	146.06016	2.865×10^9	2.795×10^9	3.860×10^9	4.623×10^9	4.879×10^9	7.872×10^9	5.597×10^9	Positive
24	*(±)*-Abscisic acid	$C_{15}H_{20}O_4$	264.13615	5.931	263.12887	3.698×10^9	3.730×10^9	5.395×10^9	4.986×10^9	5.898×10^9	4.169×10^9	3.237×10^9	Negative
25	2,2-Dimethylsuccinic acid	$C_6H_{10}O_4$	146.05804	5.501	145.05076	1.881×10^9	1.821×10^9	4.814×10^9	2.411×10^9	5.991×10^9	4.647×10^9	4.542×10^9	Negative
26	*L*-Phenylalanine	$C_9H_{11}NO_2$	165.07916	5.009	166.08644	5.889×10^9	3.870×10^9	3.216×10^9	2.211×10^9	5.425×10^9	1.860×10^9	2.606×10^9	Positive
27	*trans*-3-Hexenoic acid	$C_6H_{10}O_2$	114.06839	5.425	115.07569	3.931×10^9	3.998×10^9	3.440×10^9	3.338×10^9	2.906×10^9	3.306×10^9	3.598×10^9	Positive
28	7-Hydroxycoumarine	$C_9H_6O_3$	162.03194	5.953	163.03922	2.789×10^9	3.706×10^9	3.286×10^9	3.459×10^9	3.934×10^9	2.024×10^9	3.864×10^9	Positive
29	*α*-Lactose	$C_{12}H_{22}O_{11}$	359.14283	1.555	360.15011	3.604×10^9	3.360×10^9	3.036×10^9	3.024×10^9	2.904×10^9	3.278×10^9	2.927×10^9	Positive
30	*p*-Mentha-1,3,8-triene	$C_{10}H_{14}$	134.10974	5.6	135.11702	2.602×10^9	2.622×10^9	3.079×10^9	3.180×10^9	3.107×10^9	3.289×10^9	2.932×10^9	Positive
31	*L*-Threonic acid	$C_4H_8O_5$	136.0372	1.593	135.02992	3.193×10^9	2.945×10^9	2.600×10^9	3.190×10^9	4.181×10^9	2.299×10^9	2.034×10^9	Negative
32	Mesaconic acid	$C_5H_6O_4$	130.02673	1.687	129.01945	3.405×10^9	3.502×10^9	1.533×10^9	2.968×10^9	1.870×10^9	2.797×10^9	3.004×10^9	Negative
33	*D*-Phenylalanine	$C_9H_{11}NO_2$	165.07914	5.18	166.08645	4.472×10^9	2.983×10^9	2.624×10^9	5.945×10^8	4.506×10^9	1.502×10^8	2.151×10^9	Positive
34	*D*-(-)-Quinic acid	$C_7H_{12}O_6$	192.06396	1.37	191.05669	3.608×10^9	3.273×10^9	1.658×10^9	4.421×10^9	1.289×10^9	9.441×10^8	1.596×10^9	Negative
35	Isophorone	$C_9H_{14}O$	138.10465	6.132	139.11192	2.621×10^9	2.346×10^9	2.246×10^9	2.724×10^9	1.771×10^9	2.532×10^9	2.498×10^9	Positive
36	Valproic acid	$C_8H_{16}O_2$	144.11525	5.91	167.10449	2.918×10^9	3.034×10^9	2.108×10^9	2.685×10^9	1.852×10^9	1.578×10^9	1.854×10^9	Positive
37	Inositol	$C_6H_{12}O_6$	180.06345	1.268	198.09742	1.920×10^9	2.160×10^9	2.348×10^9	2.838×10^9	1.928×10^9	2.283×10^9	2.182×10^9	Positive
38	octadec-9-ynoic acid	$C_{18}H_{32}O_2$	262.22977	9.434	263.23704	2.461×10^9	2.525×10^9	2.102×10^9	1.899×10^9	2.489×10^9	2.055×10^9	1.916×10^9	Positive
39	Citraconic acid	$C_5H_6O_4$	130.02674	2.143	111.00889	1.825×10^9	1.865×10^9	2.697×10^9	2.164×10^9	2.319×10^9	2.049×10^9	2.314×10^9	Negative
40	*D*-(-)-Ribose	$C_5H_{10}O_5$	150.05286	1.677	149.04559	1.874×10^9	2.519×10^9	2.639×10^9	2.171×10^9	2.096×10^9	2.128×10^9	1.737×10^9	Negative
41	3-Furoic acid	$C_5H_4O_3$	112.01613	1.757	111.00885	1.889×10^9	1.721×10^9	2.088×10^9	2.036×10^9	1.958×10^9	1.817×10^9	2.424×10^9	Negative
42	Ethylmalonate	$C_5H_8O_4$	132.04239	1.459	113.02451	2.814×10^9	1.493×10^9	1.695×10^9	1.749×10^9	2.403×10^9	1.866×10^9	1.524×10^9	Negative
43	Methyl EudesMate	$C_{11}H_{14}O_5$	226.08306	5.651	453.1734	1.417×10^9	1.462×10^9	1.977×10^9	2.046×10^9	1.821×10^9	2.300×10^9	2.271×10^9	Positive
44	*D*-Tagatose	$C_6H_{12}O_6$	180.06356	5.298	198.09737	1.930×10^9	2.145×10^9	1.691×10^9	1.804×10^9	1.648×10^9	1.557×10^9	1.728×10^9	Positive

45	Propionyl-L-carnitine	$C_{10}H_{19}NO_4$	217.13151	5.673	216.12423	1.438×10^{9}	1.371×10^{9}	4.919×10^{8}	2.051×10^{9}	3.372×10^{8}	3.301×10^{9}	1.824×10^{9}	Negative
46	7-(2-aminophenyl)heptanoic acid	$C_{13}H_{19}NO_2$	221.14182	6.038	222.14909	5.785×10^{8}	5.733×10^{8}	1.588×10^{9}	1.102×10^{9}	5.592×10^{8}	3.423×10^{9}	2.864×10^{9}	Positive
47	2'-Deoxyinosine	$C_{10}H_{12}N_4O_4$	252.08452	1.436	503.1619	2.165×10^{9}	1.833×10^{9}	1.119×10^{9}	1.590×10^{9}	1.660×10^{9}	1.333×10^{9}	7.555×10^{8}	Negative
48	Palmitic Acid	$C_{16}H_{32}O_2$	273.26681	6.643	274.27408	1.538×10^{9}	2.303×10^{9}	1.381×10^{9}	1.397×10^{9}	1.170×10^{9}	1.188×10^{9}	1.106×10^{9}	Positive
49	3-Methyl-2-oxobutanoic acid	$C_5H_8O_3$	116.04769	0.664	117.05497	1.865×10^{9}	1.817×10^{9}	1.444×10^{9}	1.439×10^{9}	1.228×10^{9}	1.091×10^{9}	1.068×10^{9}	Positive
50	Carvone	$C_{10}H_{14}O$	150.1047	5.554	151.11198	1.543×10^{9}	1.985×10^{9}	1.328×10^{9}	1.566×10^{9}	1.133×10^{9}	1.245×10^{9}	1.104×10^{9}	Positive
51	4,6,8-trihydroxy-7-methoxy-3-methyl-3,4-dihydro-1H-2-benzopyran-1-one	$C_{11}H_{12}O_6$	262.04904	6.57	263.05632	1.523×10^{9}	1.429×10^{9}	1.499×10^{9}	1.487×10^{9}	7.668×10^{8}	1.624×10^{9}	1.295×10^{9}	Positive
52	TNH	$C_{14}H_{22}N_6O_6$	370.16054	5.5	371.16782	1.060×10^{9}	1.075×10^{9}	9.791×10^{8}	1.068×10^{9}	1.054×10^{9}	2.079×10^{9}	2.086×10^{9}	Positive
53	*L*-(-)-Glyceric acid	$C_3H_6O_4$	106.02666	1.59	105.01938	1.211×10^{9}	1.251×10^{9}	1.386×10^{9}	1.190×10^{9}	1.718×10^{9}	1.095×10^{9}	1.221×10^{9}	Negative
54	*δ*-Ribono-1,4-lactone	$C_5H_8O_5$	148.03733	2.868	147.03006	1.146×10^{9}	1.060×10^{9}	2.010×10^{9}	1.408×10^{9}	1.421×10^{9}	8.796×10^{8}	1.060×10^{9}	Negative
55	Choline	$C_5H_{13}NO$	103.10011	1.27	104.10738	1.434×10^{9}	1.502×10^{9}	8.560×10^{8}	1.470×10^{9}	7.808×10^{8}	1.271×10^{9}	1.268×10^{9}	Positive
56	6-Pentyl-2H-pyran-2-one	$C_{10}H_{14}O_2$	166.09959	5.563	167.10686	1.182×10^{9}	1.138×10^{9}	7.634×10^{8}	1.531×10^{9}	1.366×10^{9}	1.584×10^{9}	9.970×10^{8}	Positive
57	7-Methylxanthine	$C_6H_6N_4O_2$	166.04819	7.82	165.04091	1.897×10^{9}	1.401×10^{9}	1.388×10^{9}	1.486×10^{9}	9.835×10^{8}	7.008×10^{8}	5.616×10^{8}	Negative
58	Erythronolactone	$C_4H_6O_4$	118.02669	1.942	117.01941	1.725×10^{9}	1.668×10^{9}	1.018×10^{9}	1.345×10^{9}	9.898×10^{8}	8.004×10^{8}	7.093×10^{8}	Negative
59	Styrene	C_8H_8	104.06294	5.18	105.07026	1.715×10^{9}	9.275×10^{8}	1.521×10^{9}	3.864×10^{8}	2.455×10^{9}	4.745×10^{8}	6.844×10^{8}	Positive
60	Cyclohexanecarboxylic acid	$C_7H_{12}O_2$	128.08398	7.019	129.09122	1.181×10^{9}	1.232×10^{9}	1.080×10^{9}	1.220×10^{9}	8.962×10^{8}	9.500×10^{8}	9.942×10^{8}	Positive
61	*D*-(+)-Camphor	$C_{10}H_{16}O$	152.1203	6.323	153.12757	6.716×10^{8}	7.934×10^{8}	1.016×10^{9}	5.940×10^{8}	1.110×10^{9}	9.977×10^{8}	1.743×10^{9}	Positive
62	Inosine	$C_{10}H_{12}N_4O_5$	268.07941	1.673	267.07212	8.875×10^{8}	1.181×10^{9}	1.384×10^{9}	8.411×10^{8}	1.068×10^{9}	6.546×10^{8}	8.960×10^{8}	Negative
63	*cis*,*cis*-Muconic acid	$C_6H_6O_4$	142.02691	1.418	125.02367	9.590×10^{8}	9.902×10^{8}	8.906×10^{8}	9.939×10^{8}	9.279×10^{8}	9.568×10^{8}	9.526×10^{8}	Positive
64	β-D-Glucopyranuronic acid	$C_6H_{10}O_7$	194.04313	1.33	193.03586	9.363×10^{8}	9.706×10^{8}	2.591×10^{8}	3.470×10^{8}	2.320×10^{8}	1.333×10^{9}	2.380×10^{9}	Negative
65	*D*-2-Aminoadipic acid	$C_6H_{11}NO_4$	161.06903	1.499	162.07631	8.964×10^{8}	9.875×10^{8}	9.306×10^{8}	6.943×10^{8}	8.279×10^{8}	9.568×10^{8}	9.014×10^{8}	Positive
66	3-Hydroxysebacic acid	$C_{10}H_{18}O_5$	218.11551	5.635	217.10821	1.378×10^{9}	1.274×10^{9}	3.582×10^{8}	8.676×10^{8}	3.272×10^{8}	9.219×10^{8}	1.053×10^{9}	Negative
67	4-(4-chlorophenoxy)-3,5-dimethyl-1H-pyrazole	$C_{11}H_{11}ClN_2O$	222.0566	7.154	223.06388	1.014×10^{9}	8.231×10^{8}	9.395×10^{8}	9.576×10^{8}	4.700×10^{8}	8.509×10^{8}	7.194×10^{8}	Positive
68	Gluconolactone	$C_6H_{10}O_6$	178.04799	1.515	179.05529	9.167×10^{8}	9.261×10^{8}	6.274×10^{8}	7.472×10^{8}	6.514×10^{8}	8.948×10^{8}	7.823×10^{8}	Positive
69	Kynurenic acid	$C_{10}H_7NO_3$	189.04283	5.358	188.03556	1.376×10^{9}	8.986×10^{8}	3.821×10^{8}	4.667×10^{8}	4.928×10^{8}	7.072×10^{8}	7.877×10^{8}	Negative
70	Adenosine	$C_{10}H_{13}N_5O_4$	267.09691	3.563	268.10419	1.289×10^{9}	1.188×10^{9}	1.775×10^{8}	1.136×10^{9}	1.704×10^{8}	9.082×10^{8}	1.691×10^{8}	Positive
71	4-oxo-4,5,6,7-tetrahydrobenzo[b]furan-3-carboxylic acid	$C_9H_8O_4$	180.04237	1.392	181.04964	7.279×10^{8}	7.119×10^{8}	6.921×10^{8}	6.799×10^{8}	6.658×10^{8}	6.503×10^{8}	6.597×10^{8}	Positive
72	Octopamine	$C_8H_{11}NO_2$	153.07926	6.046	136.07597	1.168×10^{8}	1.109×10^{8}	1.410×10^{9}	1.375×10^{8}	2.461×10^{9}	1.101×10^{8}	2.702×10^{8}	Positive
73	1,5,8-Trihydroxy-9-oxo-9H-xanthen-3-yl beta-D-glucopyranoside	$C_{19}H_{18}O_{11}$	422.0855	5.32	421.07822	2.432×10^{9}	1.819×10^{9}	3.568×10^{7}	1.270×10^{8}	1.062×10^{8}	2.286×10^{7}	2.595×10^{6}	Negative
74	*trans*-Aconitic acid	$C_6H_6O_6$	174.01648	1.721	219.0146	6.510×10^{8}	7.562×10^{8}	7.285×10^{8}	5.751×10^{8}	6.228×10^{8}	5.687×10^{8}	6.166×10^{8}	Negative

75	2-Hydroxy-2-methylbutanedioic acid	$C_5H_8O_5$	148.03759	7.804	297.08245	7.785×10^8	6.398×10^8	7.554×10^8	7.701×10^8	3.127×10^8	6.199×10^8	5.501×10^8	Positive
76	JWH 018 N-(5-hydroxypentyl) metabolite	$C_{24}H_{23}NO_2$	357.17885	5.24	358.18612	2.019×10^8	1.781×10^8	1.453×10^9	3.437×10^8	7.707×10^8	2.633×10^8	9.619×10^8	Positive
77	2-Isopropylmalic acid	$C_7H_{12}O_5$	176.06867	5.497	175.0614	1.081×10^9	9.363×10^8	1.667×10^8	1.244×10^9	1.992×10^8	1.742×10^8	2.720×10^8	Negative
78	Quercetin	$C_{15}H_{10}O_7$	302.04295	5.874	301.03567	6.200×10^8	5.706×10^8	8.050×10^8	1.155×10^9	2.115×10^8	3.015×10^8	3.613×10^8	Negative
79	Camptothecin	$C_{20}H_{16}N_2O_4$	370.09409	8.57	371.10137	7.046×10^8	5.160×10^8	7.269×10^8	6.514×10^8	3.839×10^8	5.364×10^8	4.928×10^8	Positive
80	*trans*,*trans*-Muconic acid	$C_6H_6O_4$	142.02678	0.529	141.01951	5.099×10^8	5.731×10^8	5.849×10^8	5.690×10^8	5.717×10^8	5.097×10^8	5.522×10^8	Negative
81	N-Acetylornithine	$C_7H_{14}N_2O_3$	174.10069	1.737	175.10797	7.034×10^8	6.606×10^8	6.365×10^7	7.593×10^8	9.962×10^8	3.539×10^8	3.160×10^8	Positive
82	3,5-Dihydroxybenzoic acid	$C_7H_6O_4$	154.02681	5.427	153.01954	2.092×10^8	1.830×10^8	7.958×10^8	1.465×10^8	2.245×10^9	1.006×10^8	8.994×10^7	Negative
83	Arachidonoyl amide	$C_{20}H_{33}NO$	303.25388	10.882	304.26116	5.915×10^8	6.126×10^8	5.251×10^8	4.745×10^8	5.541×10^8	5.000×10^8	4.662×10^8	Positive
84	2,3-dihydroxypropyl 12-methyltridecanoate	$C_{17}H_{34}O_4$	302.24572	9.04	325.23494	5.527×10^8	5.653×10^8	5.888×10^8	5.009×10^8	5.146×10^8	5.441×10^8	4.554×10^8	Positive
85	*L*-Pyroglutamic acid	$C_5H_7NO_3$	129.04273	1.275	147.07651	7.790×10^8	7.678×10^8	2.964×10^8	1.109×10^9	1.309×10^8	3.598×10^8	1.532×10^8	Positive
86	*D*-(-)-Glutamine	$C_5H_{10}N_2O_3$	146.06926	1.277	147.0765	7.790×10^8	7.678×10^8	2.964×10^8	1.109×10^9	1.309×10^8	3.598×10^8	1.532×10^8	Positive
87	Cycloheximide	$C_{15}H_{23}NO_4$	281.16278	5.672	265.14351	4.016×10^8	3.906×10^8	3.433×10^8	6.491×10^8	3.920×10^8	6.275×10^8	7.695×10^8	Positive
88	Adenine	$C_5H_5N_5$	135.05464	1.466	136.06191	5.470×10^8	4.988×10^8	2.841×10^8	6.713×10^8	3.984×10^8	4.901×10^8	6.831×10^8	Positive
89	Pantothenic acid	$C_9H_{17}NO_5$	219.11078	5.098	218.1035	6.083×10^8	6.327×10^8	4.345×10^8	6.482×10^8	4.160×10^8	4.216×10^8	3.397×10^8	Negative
90	(2R,3S,4S,5R,6R)-2-(hydroxymethyl)-6-(propan-2-yloxy)oxane-3,4,5-triol	$C_9H_{18}O_6$	100.06422	1.303	223.11766	8.862×10^8	9.220×10^8	4.526×10^8	4.101×10^8	3.110×10^8	1.534×10^8	3.510×10^8	Positive
91	Methyl alpha-D-glucopyranoside	$C_7H_{14}O_6$	194.07972	5.998	195.08699	6.027×10^8	5.238×10^8	4.885×10^8	5.533×10^8	4.035×10^8	4.649×10^8	4.146×10^8	Positive
92	*L*-Glutamic acid	$C_5H_9NO_4$	147.05319	1.322	148.06047	8.521×10^8	7.155×10^8	2.045×10^8	1.026×10^9	2.186×10^8	2.583×10^8	1.356×10^8	Positive
93	3-Hydroxybenzoic acid	$C_7H_6O_3$	138.03177	5.996	137.02449	6.872×10^8	5.553×10^8	5.573×10^8	5.251×10^8	5.847×10^8	1.723×10^8	1.933×10^8	Negative
94	*DL*-Stachydrine	$C_7H_{13}NO_2$	143.09489	1.441	144.10216	3.608×10^8	2.939×10^8	4.518×10^8	3.891×10^8	7.803×10^8	3.966×10^8	5.533×10^8	Positive
95	5-(hydroxymethyl)-4-methoxy-2,5-dihydrofuran-2-one	$C_6H_8O_4$	166.02446	9.654	167.03174	4.477×10^8	4.861×10^8	4.250×10^8	4.628×10^8	4.203×10^8	3.812×10^8	3.877×10^8	Positive
96	*D*-Erythrose 4-phosphate	$C_4H_9O_7P$	200.00907	1.222	201.01634	5.104×10^8	5.049×10^8	3.700×10^8	5.161×10^8	4.568×10^8	2.537×10^8	2.995×10^8	Positive
97	2-Methoxyresorcinol	$C_7H_8O_3$	140.04754	5.562	141.05477	3.667×10^8	3.565×10^8	2.644×10^8	5.260×10^8	5.261×10^8	5.414×10^8	3.133×10^8	Positive
98	Oleoyl ethanolamide	$C_{20}H_{39}NO_2$	307.28762	10.257	330.27687	4.471×10^8	4.629×10^8	3.961×10^8	3.473×10^8	4.375×10^8	3.721×10^8	3.832×10^8	Positive
99	Monobutyl phthalate	$C_{12}H_{14}O_4$	222.08696	5.983	221.07966	5.195×10^8	5.223×10^8	2.166×10^8	3.733×10^8	5.191×10^8	3.826×10^8	3.112×10^8	Negative
100	N-Tetradecanamide	$C_{14}H_{29}NO$	227.22509	8.786	228.23236	4.462×10^8	4.358×10^8	3.993×10^8	3.455×10^8	4.461×10^8	3.993×10^8	3.640×10^8	Positive
101	Sedanolide	$C_{12}H_{18}O_2$	176.12032	5.563	177.12761	3.876×10^8	3.714×10^8	2.601×10^8	5.108×10^8	4.967×10^8	4.976×10^8	3.009×10^8	Positive
102	3-methyl-5-oxo-5-(4-toluidino)pentanoic acid	$C_{13}H_{17}NO_3$	257.10281	1.282	258.11009	2.648×10^8	2.980×10^8	4.881×10^8	2.426×10^8	7.274×10^8	3.065×10^8	4.457×10^8	Positive
103	5-acetyl-2,6-dimethyl-1,2,3,4-tetrahydropyridin-4-one	$C_9H_{13}NO_2$	167.09487	5.951	168.10215	6.814×10^7	6.634×10^7	1.046×10^9	1.374×10^8	2.812×10^8	4.130×10^8	7.168×10^8	Positive
104	4-Methyl-2-Oxopentanoic Acid	$C_6H_{10}O_3$	130.06336	5.988	113.06009	3.343×10^8	3.479×10^8	6.953×10^8	3.417×10^8	2.918×10^8	3.851×10^8	3.151×10^8	Positive
105	1-(3,4-dimethoxyphenyl)ethan-1-one oxime	$C_{10}H_{13}NO_3$	195.08991	5.763	196.09719	1.473×10^8	1.593×10^8	6.377×10^8	2.568×10^8	1.110×10^9	2.012×10^8	1.912×10^8	Positive

106	*DL*-Lysine	$C_6H_{14}N_2O_2$	146.1057	1.179	147.11294	3.586×10^{8}	4.132×10^{8}	3.717×10^{8}	3.561×10^{8}	3.173×10^{8}	4.275×10^{8}	3.660×10^{8}	Positive
107	2,4-Dimethylbenzaldehyde	$C_9H_{10}O$	134.07345	5.562	135.08072	3.450×10^{8}	3.390×10^{8}	2.861×10^{8}	4.449×10^{8}	4.415×10^{8}	4.213×10^{8}	2.845×10^{8}	Positive
108	Suberic acid	$C_8H_{14}O_4$	174.08938	5.7	173.08208	2.986×10^{8}	3.003×10^{8}	3.659×10^{8}	2.960×10^{8}	5.209×10^{8}	3.337×10^{8}	3.522×10^{8}	Negative
109	Phenylglyoxylic acid	$C_8H_6O_3$	150.03181	1.393	151.03906	3.806×10^{8}	2.316×10^{8}	3.676×10^{8}	3.731×10^{8}	3.881×10^{8}	3.542×10^{8}	3.567×10^{8}	Positive
110	Syringic acid	$C_9H_{10}O_5$	198.05295	1.38	199.06027	3.760×10^{8}	3.732×10^{8}	3.734×10^{8}	3.662×10^{8}	3.537×10^{8}	2.289×10^{8}	3.730×10^{8}	Positive
111	1-(3-acetyl-2,4,6-trihydroxyphenyl)ethan-1-one	$C_{10}H_{10}O_5$	210.05303	1.385	211.0603	3.666×10^{8}	3.035×10^{8}	3.637×10^{8}	3.618×10^{8}	3.532×10^{8}	3.395×10^{8}	3.529×10^{8}	Positive
112	Fumaric acid	$C_4H_4O_4$	116.01102	1.955	115.00374	4.225×10^{8}	4.334×10^{8}	3.509×10^{8}	3.769×10^{8}	3.583×10^{8}	2.058×10^{8}	1.944×10^{8}	Negative
113	*cis*-Aconitic acid	$C_6H_6O_6$	174.0166	2.263	173.00933	3.803×10^{8}	3.616×10^{8}	2.914×10^{8}	3.737×10^{8}	3.075×10^{8}	2.757×10^{8}	2.685×10^{8}	Negative
114	4-(3,4-dihydro-2H-1,5-benzodioxepin-7-yl)-4-oxobutanoic acid	$C_{13}H_{14}O_5$	250.08776	5.773	249.08048	2.276×10^{8}	2.121×10^{8}	2.629×10^{8}	3.222×10^{8}	3.498×10^{8}	5.291×10^{8}	3.154×10^{8}	Negative
115	4-Hydroxybenzaldehyde	$C_7H_6O_2$	122.03709	5.987	123.04437	1.557×10^{8}	1.542×10^{8}	2.289×10^{8}	2.446×10^{8}	1.501×10^{8}	5.719×10^{8}	6.617×10^{8}	Positive
116	*L*-Tyrosinemethylester	$C_{10}H_{13}NO_3$	195.08973	7.025	194.08245	2.779×10^{8}	3.205×10^{8}	3.105×10^{8}	3.077×10^{8}	3.173×10^{8}	2.858×10^{8}	3.084×10^{8}	Negative
117	3,4,5-trihydroxycyclohex-1-ene-1-carboxylic acid	$C_7H_{10}O_5$	174.05286	1.706	173.04558	5.627×10^{8}	6.112×10^{8}	5.029×10^{7}	7.444×10^{8}	5.712×10^{7}	5.044×10^{7}	4.642×10^{7}	Negative
118	2-Amino-1,3-octadecanediol	$C_{18}H_{39}NO_2$	301.2981	7.166	302.30537	2.909×10^{8}	3.050×10^{8}	3.207×10^{8}	3.068×10^{8}	3.050×10^{8}	2.997×10^{8}	2.457×10^{8}	Positive
119	6-Deoxy-D-glucose	$C_6H_{12}O_5$	164.06847	1.687	209.06663	4.998×10^{8}	4.520×10^{8}	1.049×10^{8}	6.432×10^{8}	1.923×10^{8}	1.025×10^{8}	7.038×10^{7}	Negative
120	*α,α*-Trehalose	$C_{12}H_{22}O_{11}$	342.11675	5.019	341.10939	2.636×10^{8}	2.602×10^{8}	2.952×10^{8}	1.959×10^{8}	1.786×10^{8}	6.401×10^{8}	2.044×10^{8}	Negative
121	2,5-Furandicarboxylic acid	$C_6H_4O_5$	156.00613	2.118	139.00283	2.408×10^{8}	2.774×10^{8}	2.855×10^{8}	2.854×10^{8}	2.770×10^{8}	3.492×10^{8}	2.945×10^{8}	Positive
122	Ethyl-β-D-glucuronide	$C_8H_{14}O_7$	222.07391	1.315	221.06666	3.365×10^{8}	3.114×10^{8}	2.587×10^{8}	2.837×10^{8}	3.239×10^{8}	2.105×10^{8}	2.669×10^{8}	Negative
123	Stachyose	$C_{24}H_{42}O_{21}$	666.22408	1.452	665.21652	3.250×10^{8}	3.265×10^{8}	1.153×10^{8}	4.592×10^{8}	3.739×10^{8}	2.334×10^{8}	1.563×10^{8}	Negative
124	Cuminaldehyde	$C_{10}H_{12}O$	148.08904	5.663	149.09631	3.026×10^{8}	2.955×10^{8}	2.265×10^{8}	2.710×10^{8}	2.772×10^{8}	3.141×10^{8}	2.655×10^{8}	Positive
125	5-(6-hydroxy-6-methyloctyl)-2,5-dihydrofuran-2-one	$C_{13}H_{22}O_3$	208.14661	6.047	209.15391	2.236×10^{8}	2.232×10^{8}	3.471×10^{8}	2.858×10^{8}	3.355×10^{8}	2.422×10^{8}	2.890×10^{8}	Positive
126	*o*-Cresol	C_7H_8O	108.05795	5.113	109.06522	2.422×10^{8}	2.211×10^{8}	2.544×10^{8}	2.667×10^{8}	3.388×10^{8}	2.963×10^{8}	2.947×10^{8}	Positive
127	Trigonelline	$C_7H_7NO_2$	137.04781	1.383	138.05509	4.900×10^{8}	4.173×10^{8}	1.343×10^{8}	5.251×10^{8}	1.502×10^{8}	9.964×10^{7}	8.868×10^{7}	Positive
128	(2E)-4-Hydroxy-3,7-dimethyl-2,6-octadien-1-yl beta-D-glucopyranoside	$C_{16}H_{28}O_7$	349.21001	5.904	350.21729	3.305×10^{8}	3.379×10^{8}	2.169×10^{8}	2.801×10^{8}	2.497×10^{8}	2.984×10^{8}	1.796×10^{8}	Positive
129	(-)-Ephedrine	$C_{10}H_{15}NO$	165.11565	5.293	166.12293	1.289×10^{8}	1.263×10^{8}	3.557×10^{8}	2.090×10^{8}	3.306×10^{8}	3.150×10^{8}	3.707×10^{8}	Positive
130	Luteolin	$C_{15}H_{10}O_6$	286.048	6.079	285.04073	3.057×10^{8}	3.012×10^{8}	3.358×10^{8}	5.713×10^{8}	8.111×10^{7}	1.087×10^{8}	1.305×10^{8}	Negative
131	2-(2-acetyl-3,5-dihydroxyphenyl)acetic acid	$C_{10}H_{10}O_5$	192.04251	1.39	193.04979	2.833×10^{8}	2.813×10^{8}	2.803×10^{8}	2.818×10^{8}	2.407×10^{8}	1.759×10^{8}	2.790×10^{8}	Positive
132	2-Amino-1,3,4-octadecanetriol	$C_{18}H_{39}NO_3$	317.29302	6.633	318.30029	2.565×10^{8}	4.191×10^{8}	2.372×10^{8}	2.815×10^{8}	2.139×10^{8}	2.185×10^{8}	1.837×10^{8}	Positive
133	Adipic acid	$C_6H_{10}O_4$	146.05794	5.271	145.05062	3.488×10^{8}	3.774×10^{8}	1.163×10^{8}	3.264×10^{8}	5.824×10^{7}	3.153×10^{8}	2.504×10^{8}	Negative
134	Elaidic acid	$C_{18}H_{34}O_2$	282.25594	10.685	281.24865	2.139×10^{8}	2.299×10^{8}	2.730×10^{8}	1.354×10^{8}	5.088×10^{8}	2.117×10^{8}	1.978×10^{8}	Negative
135	Epinephrine	$C_9H_{13}NO_3$	183.08981	5.841	184.09709	1.019×10^{8}	1.036×10^{8}	5.186×10^{8}	1.848×10^{8}	4.545×10^{8}	1.538×10^{8}	2.471×10^{8}	Positive

136	2-[5-(2-hydroxypropyl)oxolan-2-yl]propanoic acid	$C_{10}H_{18}O_4$	112.05138	5.508	225.11005	3.336×10^{8}	2.517×10^{8}	1.236×10^{8}	2.796×10^{8}	2.511×10^{8}	3.223×10^{8}	1.913×10^{8}	Positive
137	Paraxanthine	$C_7H_8N_4O_2$	180.06382	6	181.07131	2.963×10^{8}	2.911×10^{8}	2.567×10^{8}	2.852×10^{8}	2.051×10^{8}	2.421×10^{8}	1.440×10^{8}	Positive
138	Homovanillic acid	$C_9H_{10}O_4$	182.05809	1.399	183.06537	2.510×10^{8}	2.674×10^{8}	2.439×10^{8}	2.526×10^{8}	2.406×10^{8}	2.166×10^{8}	2.326×10^{8}	Positive
139	5-Hydroxyindole	C_8H_7NO	133.05308	5.445	178.05128	1.455×10^{8}	2.289×10^{8}	1.878×10^{8}	3.435×10^{8}	4.182×10^{8}	1.826×10^{8}	1.954×10^{8}	Negative
140	Hippuric acid	$C_9H_9NO_3$	179.05854	5.465	178.05127	2.006×10^{8}	2.289×10^{8}	1.878×10^{8}	2.753×10^{8}	4.097×10^{8}	1.837×10^{8}	1.985×10^{8}	Negative
141	AICA ribonucleotide	$C_9H_{15}N_4O_8P$	338.06416	7.154	339.07143	3.103×10^{8}	2.413×10^{8}	2.822×10^{8}	2.829×10^{8}	1.087×10^{8}	2.430×10^{8}	2.070×10^{8}	Positive
142	*L*-(+)-Tartaric acid	$C_4H_6O_6$	168.02755	7.82	149.00971	3.971×10^{8}	2.785×10^{8}	2.877×10^{8}	2.995×10^{8}	1.567×10^{8}	1.293×10^{8}	1.074×10^{8}	Negative
143	Isoproterenol	$C_{11}H_{17}NO_3$	211.12114	5.409	212.12842	1.054×10^{8}	1.027×10^{8}	4.461×10^{8}	1.899×10^{8}	3.098×10^{8}	1.990×10^{8}	3.028×10^{8}	Positive
144	4-Oxoproline	$C_5H_7NO_3$	129.0427	1.888	128.03543	2.508×10^{8}	3.499×10^{8}	1.945×10^{8}	3.749×10^{8}	1.361×10^{8}	1.313×10^{8}	1.863×10^{8}	Negative
145	Quinic acid	$C_7H_{12}O_6$	192.06361	1.534	193.07089	2.719×10^{8}	2.790×10^{8}	1.331×10^{8}	2.922×10^{8}	9.417×10^{7}	2.518×10^{8}	2.491×10^{8}	Positive
146	Benzoic acid	$C_7H_6O_2$	122.03711	5.558	123.04437	1.907×10^{8}	1.943×10^{8}	1.478×10^{8}	2.844×10^{8}	2.771×10^{8}	2.831×10^{8}	1.543×10^{8}	Positive
147	5,5-dimethyl-3-morpholinocyclohex-2-en-1-one	$C_{12}H_{19}NO_2$	209.14184	5.881	210.14913	1.346×10^{8}	1.239×10^{8}	1.922×10^{8}	1.968×10^{8}	1.353×10^{8}	3.556×10^{8}	3.350×10^{8}	Positive
148	gamma-Nonanolactone	$C_9H_{16}O_2$	156.11524	5.648	155.10796	2.404×10^{8}	2.246×10^{8}	1.481×10^{8}	1.721×10^{8}	3.218×10^{8}	1.739×10^{8}	1.887×10^{8}	Negative
149	N-Acetyl-aspartic acid	$C_6H_9NO_5$	175.04832	1.469	176.0556	1.434×10^{8}	1.308×10^{8}	2.144×10^{8}	1.352×10^{8}	2.817×10^{8}	3.037×10^{8}	2.520×10^{8}	Positive
150	Dodecanedioic acid	$C_{12}H_{22}O_4$	230.15178	6.529	229.1445	3.322×10^{8}	3.106×10^{8}	7.629×10^{7}	2.828×10^{8}	5.745×10^{7}	2.799×10^{8}	1.121×10^{8}	Negative
151	Heroin-d3	$C_{21}H_{20}[2]H_3NO_5$	350.19427	5.86	373.18355	2.789×10^{8}	2.722×10^{8}	1.556×10^{8}	2.402×10^{8}	1.573×10^{8}	1.806×10^{8}	1.664×10^{8}	Positive
152	*D*-Raffinose	$C_{18}H_{32}O_{16}$	504.16906	5.045	503.16177	3.389×10^{8}	3.734×10^{8}	1.289×10^{8}	2.261×10^{8}	1.302×10^{8}	1.516×10^{8}	8.177×10^{7}	Negative
153	*DL*-Arginine	$C_6H_{14}N_4O_2$	174.1119	1.26	175.11917	2.112×10^{8}	2.340×10^{8}	2.040×10^{8}	2.546×10^{8}	1.123×10^{8}	2.594×10^{8}	1.308×10^{8}	Positive
154	3-Hydroxydecanoic acid	$C_{10}H_{20}O_3$	142.13591	6.754	187.13411	2.915×10^{8}	2.662×10^{8}	9.696×10^{7}	2.338×10^{8}	1.570×10^{8}	2.623×10^{8}	9.446×10^{7}	Negative
155	N-Acetyl-L-leucine	$C_8H_{15}NO_3$	173.10548	5.726	174.11276	1.446×10^{8}	1.287×10^{8}	1.268×10^{8}	2.083×10^{8}	1.649×10^{8}	4.053×10^{8}	2.144×10^{8}	Positive
156	3-Methylcrotonylglycine	$C_7H_{11}NO_3$	157.07416	1.828	158.08143	2.240×10^{8}	2.657×10^{8}	1.265×10^{8}	1.923×10^{8}	2.520×10^{8}	1.607×10^{8}	1.031×10^{8}	Positive
157	2-(4-methoxyphenyl)-3-(1H-pyrazol-4-yl)acrylonitrile	$C_{13}H_{11}N_3O$	263.04832	6.565	264.0556	1.978×10^{8}	1.858×10^{8}	1.980×10^{8}	2.069×10^{8}	1.027×10^{8}	2.151×10^{8}	1.688×10^{8}	Positive
158	12-Hydroxydodecanoic acid	$C_{12}H_{24}O_3$	216.1727	6.594	215.16542	3.001×10^{8}	2.569×10^{8}	7.013×10^{7}	2.572×10^{8}	7.121×10^{7}	2.313×10^{8}	7.583×10^{7}	Negative
159	2-Hydroxy-3-methylbutanoic acid	$C_5H_{10}O_3$	118.0631	4.965	117.05583	2.034×10^{8}	1.618×10^{8}	2.245×10^{8}	1.802×10^{8}	1.853×10^{8}	1.473×10^{8}	1.593×10^{8}	Negative
160	Phenylacetylglycine	$C_{10}H_{11}NO_3$	147.06871	5.558	192.06696	1.730×10^{8}	1.579×10^{8}	1.225×10^{8}	3.328×10^{8}	1.207×10^{8}	1.923×10^{8}	1.582×10^{8}	Negative
161	Hypoxanthine	$C_5H_4N_4O$	136.03876	2.046	137.04604	2.188×10^{8}	2.455×10^{8}	7.865×10^{7}	2.671×10^{8}	1.810×10^{8}	1.348×10^{8}	1.123×10^{8}	Positive
162	Naringenin	$C_{15}H_{12}O_5$	272.06864	6.042	271.06137	4.198×10^{7}	4.899×10^{7}	5.329×10^{8}	7.603×10^{7}	4.169×10^{8}	3.740×10^{7}	8.208×10^{7}	Negative
163	N-(2,6-Dimethylphenyl)-N-(methoxyacetyl) alanine	$C_{14}H_{19}NO_4$	265.1314	5.179	266.13868	3.267×10^{8}	1.419×10^{8}	1.938×10^{8}	1.553×10^{7}	3.694×10^{8}	1.444×10^{7}	1.677×10^{8}	Positive
164	*L*-Glutathione oxidized	$C_{20}H_{32}N_6O_{12}S_2$	612.15206	2.437	307.0833	3.434×10^{8}	3.129×10^{8}	1.235×10^{7}	4.528×10^{8}	7.609×10^{7}	2.370×10^{7}	7.604×10^{6}	Positive
165	*L*-Aspartic acid	$C_4H_7NO_4$	133.0377	1.276	134.04497	2.606×10^{8}	3.861×10^{8}	4.257×10^{7}	2.439×10^{8}	2.533×10^{7}	2.005×10^{8}	4.642×10^{7}	Positive
166	1-Aminocyclohexanecarboxylic acid	$C_7H_{13}NO_2$	143.09497	1.761	144.10225	1.953×10^{8}	1.524×10^{8}	1.951×10^{8}	1.434×10^{8}	2.274×10^{8}	1.279×10^{8}	1.624×10^{8}	Positive
167	Indole-3-acrylic acid	$C_{11}H_9NO_2$	187.06357	5.269	188.07084	1.898×10^{8}	1.797×10^{8}	1.742×10^{8}	2.066×10^{8}	1.897×10^{8}	1.434×10^{8}	1.039×10^{8}	Positive

168	Asparagine	$C_4H_8N_2O_3$	132.05371	1.271	133.06097	2.964×10^{8}	3.169×10^{8}	4.566×10^{7}	3.514×10^{8}	3.793×10^{7}	7.794×10^{7}	5.903×10^{7}	Positive
169	10-Hydroxydecanoic acid	$C_{10}H_{20}O_3$	188.14145	6.146	187.13417	3.673×10^{8}	3.395×10^{8}	4.254×10^{7}	2.137×10^{8}	3.591×10^{7}	1.247×10^{8}	5.333×10^{7}	Negative
170	*D*-Glucose 6-phosphate	$C_6H_{13}O_9P$	260.02964	1.279	259.02237	1.498×10^{8}	1.519×10^{8}	1.498×10^{8}	1.203×10^{8}	1.379×10^{8}	2.032×10^{8}	2.326×10^{8}	Negative
171	Gamma-Caprolactone	$C_6H_{10}O_2$	114.06822	5.564	159.06651	1.544×10^{8}	1.455×10^{8}	2.066×10^{8}	1.833×10^{8}	2.687×10^{8}	9.176×10^{7}	9.347×10^{7}	Negative
172	6-(4-methoxyphenyl)pyrimidine-2,4-diamine	$C_{11}H_{12}N_4O$	216.09997	5.643	215.09269	9.932×10^{7}	1.617×10^{8}	2.340×10^{8}	1.147×10^{8}	2.538×10^{8}	1.324×10^{8}	1.455×10^{8}	Negative
173	Lumefantrine	$C_{30}H_{32}Cl_3NO$	527.15535	6.034	528.16262	3.051×10^{8}	2.964×10^{8}	6.179×10^{7}	2.364×10^{8}	7.048×10^{7}	8.891×10^{7}	7.546×10^{7}	Positive
174	4-Butylresorcinol	$C_{10}H_{14}O_2$	166.09949	5.886	165.09221	1.154×10^{8}	1.242×10^{8}	1.932×10^{8}	1.319×10^{8}	3.196×10^{8}	1.366×10^{8}	1.122×10^{8}	Negative
175	Pipecolic acid	$C_6H_{11}NO_2$	129.07917	1.181	130.08652	1.518×10^{8}	1.730×10^{8}	1.515×10^{8}	1.401×10^{8}	1.411×10^{8}	1.778×10^{8}	1.507×10^{8}	Positive
176	Deoxyinosine	$C_{10}H_{12}N_4O_4$	252.08479	1.292	543.13287	2.476×10^{8}	1.668×10^{8}	9.670×10^{7}	2.055×10^{8}	1.827×10^{8}	6.703×10^{7}	1.060×10^{8}	Positive
177	Maltotriose	$C_{18}H_{32}O_{16}$	504.16955	1.446	505.17662	1.923×10^{8}	1.979×10^{8}	1.135×10^{8}	1.574×10^{8}	1.334×10^{8}	1.513×10^{8}	1.201×10^{8}	Positive
178	*L*-Histidine	$C_6H_9N_3O_2$	155.06964	1.262	156.07691	1.519×10^{8}	1.739×10^{8}	1.745×10^{8}	1.687×10^{8}	1.540×10^{8}	1.265×10^{8}	1.031×10^{8}	Positive
179	6-methyl-4-(morpholinomethyl)-2H-chromen-2-one	$C_{15}H_{17}NO_3$	259.12089	5.594	260.12816	1.061×10^{8}	9.995×10^{7}	2.109×10^{8}	1.918×10^{8}	1.383×10^{8}	1.476×10^{8}	1.515×10^{8}	Positive
180	7-(2-hydroxypropan-2-yl)-1,4a-dimethyl-decahydronaphthalen-1-ol	$C_{15}H_{28}O_2$	222.19843	9.921	240.23225	1.692×10^{8}	1.630×10^{8}	1.444×10^{8}	1.344×10^{8}	1.599×10^{8}	1.404×10^{8}	1.299×10^{8}	Positive
181	3-Methyladipic acid	$C_7H_{12}O_4$	160.0737	5.654	159.06641	1.236×10^{8}	1.155×10^{8}	2.066×10^{8}	1.467×10^{8}	2.687×10^{8}	9.176×10^{7}	7.486×10^{7}	Negative
182	Rutin	$C_{27}H_{30}O_{16}$	610.1547	5.507	609.14742	1.144×10^{8}	1.402×10^{8}	2.518×10^{8}	3.715×10^{8}	3.352×10^{7}	7.991×10^{7}	3.244×10^{7}	Negative
183	Ecgonine methyl ester	$C_{10}H_{17}NO_3$	199.12117	5.494	200.12845	8.877×10^{7}	6.032×10^{7}	2.504×10^{8}	1.171×10^{8}	1.555×10^{8}	1.862×10^{8}	1.343×10^{8}	Positive
184	N-Acetyl-L-phenylalanine	$C_{11}H_{13}NO_3$	207.08977	5.593	206.0825	2.316×10^{8}	1.812×10^{8}	6.296×10^{7}	2.025×10^{8}	1.104×10^{8}	9.432×10^{7}	9.538×10^{7}	Negative
185	4-Methoxycinnamaldehyde	$C_{10}H_{10}O_2$	162.06824	5.902	163.07554	1.466×10^{8}	1.263×10^{8}	1.482×10^{8}	1.548×10^{8}	1.996×10^{8}	9.918×10^{7}	9.591×10^{7}	Positive
186	Uracil	$C_4H_4N_2O_2$	112.02771	1.936	113.03499	1.848×10^{8}	1.457×10^{8}	8.260×10^{7}	1.629×10^{8}	1.149×10^{8}	1.447×10^{8}	1.338×10^{8}	Positive
187	Apigenin	$C_{15}H_{10}O_5$	270.0532	6.094	315.05145	2.279×10^{8}	1.805×10^{8}	1.571×10^{8}	1.230×10^{8}	1.072×10^{8}	7.363×10^{7}	9.890×10^{7}	Negative
188	3,8,9-trihydroxy-10-propyl-3,4,5,8,9,10-hexahydro-2H-oxecin-2-one	$C_{12}H_{20}O_5$	244.13119	5.348	243.12391	7.105×10^{7}	8.718×10^{7}	2.343×10^{8}	1.077×10^{8}	2.005×10^{8}	1.212×10^{8}	1.401×10^{8}	Negative
189	AKB48 N-(5-hydroxypentyl) metabolite	$C_{23}H_{31}N_3O_2$	403.22089	5.34	404.22816	6.522×10^{7}	6.361×10^{7}	2.512×10^{8}	1.046×10^{8}	3.056×10^{8}	7.461×10^{7}	9.412×10^{7}	Positive
190	alpha-Ketoglutaric acid	$C_5H_6O_5$	146.02166	2.116	147.02894	1.122×10^{8}	1.309×10^{8}	1.397×10^{8}	1.357×10^{8}	1.320×10^{8}	1.661×10^{8}	1.418×10^{8}	Positive
191	Uridine	$C_9H_{12}N_2O_6$	244.06953	2.391	243.06223	1.569×10^{8}	1.607×10^{8}	6.451×10^{7}	1.457×10^{8}	1.388×10^{8}	1.364×10^{8}	1.316×10^{8}	Negative
192	3-phenyl-5-[3-(trifluoromethyl)-1H-pyrazol-1-yl]-1,2,4-thiadiazole	$C_{12}H_7F_3N_4S$	296.03894	7.803	297.04622	1.550×10^{8}	1.308×10^{8}	1.522×10^{8}	1.622×10^{8}	7.813×10^{7}	1.355×10^{8}	1.171×10^{8}	Positive
193	Phenylpyruvic acid	$C_9H_8O_3$	164.04749	5.758	209.04569	3.215×10^{8}	2.522×10^{8}	9.703×10^{6}	2.967×10^{8}	1.345×10^{7}	1.010×10^{7}	7.896×10^{6}	Negative
194	Dimetghyl 4-Hydroxyisophthalate	$C_{10}H_{10}O_5$	210.05297	5.758	209.0457	3.215×10^{8}	2.522×10^{8}	9.218×10^{6}	2.967×10^{8}	1.294×10^{7}	9.701×10^{6}	7.532×10^{6}	Negative
195	*DL*-4-Hydroxyphenyllactic acid	$C_9H_{10}O_4$	182.05805	5.283	181.05078	1.750×10^{8}	1.897×10^{8}	1.068×10^{8}	1.997×10^{8}	7.039×10^{7}	5.959×10^{7}	9.698×10^{7}	Negative
196	1-(4-hydroxyphenyl)propane-1,2-diol	$C_9H_{12}O_3$	150.06841	5.664	151.07574	1.435×10^{8}	1.042×10^{8}	1.003×10^{8}	2.079×10^{8}	1.225×10^{8}	1.073×10^{8}	1.092×10^{8}	Positive

197	Dipropylene glycol dimethyl ether	$C_8H_{18}O_3$	162.12574	5.951	163.13305	1.406×10^{8}	1.564×10^{8}	1.286×10^{8}	1.336×10^{8}	1.222×10^{8}	1.102×10^{8}	1.010×10^{8}	Positive
198	1-(4-methoxyphenyl)propane-1,2-diol	$C_{10}H_{14}O_3$	164.08405	5.997	165.09137	1.186×10^{8}	8.559×10^{7}	1.010×10^{8}	1.130×10^{8}	1.421×10^{8}	1.693×10^{8}	1.620×10^{8}	Positive
199	*o*-Toluic Acid	$C_8H_8O_2$	136.05269	5.332	181.05092	1.755×10^{8}	1.824×10^{8}	1.068×10^{8}	1.969×10^{8}	7.228×10^{7}	5.959×10^{7}	9.698×10^{7}	Negative
200	2-(4-aminophenoxy)isophthalonitrile	$C_{14}H_9N_3O$	235.07244	1.234	236.07971	4.887×10^{7}	5.138×10^{7}	1.492×10^{8}	8.282×10^{7}	2.062×10^{8}	1.262×10^{8}	2.051×10^{8}	Positive
201	Methyldopa	$C_{10}H_{13}NO_4$	211.08478	5.368	212.09207	4.897×10^{7}	4.691×10^{7}	2.011×10^{8}	7.783×10^{7}	1.144×10^{8}	1.769×10^{8}	1.919×10^{8}	Positive
202	(2R,3S,4S,5R,6R)-2-(hydroxymethyl)-6-(2-phenylethoxy)oxane-3,4,5-triol	$C_{14}H_{20}O_6$	306.10792	5.657	307.11519	8.833×10^{7}	8.284×10^{7}	1.530×10^{8}	1.353×10^{8}	1.347×10^{8}	1.427×10^{8}	1.141×10^{8}	Positive
203	*DL*-Tryptophan	$C_{11}H_{12}N_2O_2$	204.09016	5.272	205.09746	1.647×10^{8}	1.013×10^{8}	1.482×10^{8}	1.745×10^{8}	5.213×10^{7}	1.206×10^{8}	8.479×10^{7}	Positive
204	Prolylleucine	$C_{11}H_{20}N_2O_3$	228.14754	2.022	229.15482	2.712×10^{8}	2.152×10^{8}	2.874×10^{7}	2.397×10^{8}	3.008×10^{7}	2.778×10^{7}	2.443×10^{7}	Positive
205	1-(4-Methoxyphenyl)-2-propanone	$C_{10}H_{12}O_2$	164.08406	5.352	165.09134	1.465×10^{8}	1.081×10^{8}	7.371×10^{7}	1.526×10^{8}	2.090×10^{8}	8.137×10^{7}	6.095×10^{7}	Positive
206	3,4-dihydro-2H,6H-[1,3]thiazino[2,3-b]quinazolin-6-one	$C_{11}H_{10}N_2OS$	240.03067	6.568	241.03794	1.390×10^{8}	1.201×10^{8}	1.271×10^{8}	1.317×10^{8}	6.948×10^{7}	1.343×10^{8}	1.068×10^{8}	Positive
207	(2E,4E)-N-(2-methylpropyl)dodeca-2,4-dienamide	$C_{16}H_{29}NO$	251.22507	8.605	252.23237	1.320×10^{8}	1.316×10^{8}	1.113×10^{8}	1.045×10^{8}	1.295×10^{8}	1.097×10^{8}	1.088×10^{8}	Positive
208	2-(Formylamino)Benzoic Acid	$C_8H_7NO_3$	165.04281	5.758	164.03554	5.568×10^{7}	5.404×10^{7}	1.742×10^{8}	6.861×10^{7}	3.396×10^{8}	1.027×10^{8}	3.199×10^{7}	Negative
209	Lipoic acid	$C_8H_{14}O_2S_2$	206.04281	1.959	205.03543	1.448×10^{8}	1.401×10^{8}	9.773×10^{7}	1.642×10^{8}	1.037×10^{8}	8.476×10^{7}	8.544×10^{7}	Negative
210	4-methoxy-6-[2-(4-methoxyphenyl)ethyl]-2H-pyran-2-one	$C_{15}H_{16}O_4$	138.03611	7.804	299.06152	1.328×10^{8}	1.180×10^{8}	1.364×10^{8}	1.363×10^{8}	6.255×10^{7}	1.193×10^{8}	1.106×10^{8}	Positive
211	Guanosine	$C_{10}H_{13}N_5O_5$	283.09169	3.853	282.08442	1.474×10^{8}	1.371×10^{8}	8.310×10^{7}	1.367×10^{8}	9.431×10^{7}	1.282×10^{8}	8.051×10^{7}	Negative
212	4-({[2-(2-oxo-1-imidazolidinyl)ethyl]imino}methyl)benzonitrile	$C_{13}H_{14}N_4O$	242.11325	5.291	243.12052	1.882×10^{8}	1.503×10^{8}	7.191×10^{7}	1.041×10^{8}	4.767×10^{7}	9.694×10^{7}	1.375×10^{8}	Positive
213	Salicylic acid	$C_7H_6O_3$	138.03179	5.759	137.02451	2.479×10^{8}	1.952×10^{8}	2.755×10^{7}	2.284×10^{8}	4.072×10^{7}	2.297×10^{7}	2.311×10^{7}	Negative
214	2-Phenylacetamide	C_8H_9NO	135.0687	5.502	136.07596	3.935×10^{7}	4.547×10^{7}	2.003×10^{8}	7.308×10^{7}	2.073×10^{8}	7.056×10^{7}	1.494×10^{8}	Positive
215	N-Acetylneuraminic acid	$C_{11}H_{19}NO_9$	309.10621	1.844	308.09888	1.666×10^{8}	1.631×10^{8}	6.727×10^{7}	1.657×10^{8}	5.765×10^{7}	4.506×10^{7}	8.381×10^{7}	Negative
216	N'1-methyl-N'1-[2-nitro-4-(trifluoromethyl)phenyl]ethanohydrazide	$C_{10}H_{10}F_3N_3O_3$	277.06378	6.747	278.07105	1.260×10^{8}	1.112×10^{8}	1.135×10^{8}	1.215×10^{8}	5.522×10^{7}	1.242×10^{8}	9.512×10^{7}	Positive
217	N1-methyl-5-methoxy-2-({2-[(methylamino)carbonyl]phenyl}thio)benzamide	$C_{17}H_{18}N_2O_3S$	352.07976	7.862	353.08704	1.420×10^{8}	1.010×10^{8}	1.335×10^{8}	1.373×10^{8}	8.765×10^{6}	1.086×10^{8}	9.352×10^{7}	Positive
218	*L*-Glutamate	$C_5H_9NO_4$	147.05321	1.509	148.06049	2.123×10^{8}	1.475×10^{8}	5.184×10^{7}	1.655×10^{8}	4.771×10^{7}	6.504×10^{7}	3.442×10^{7}	Positive
219	Quercetin-3β-D-glucoside	$C_{21}H_{20}O_{12}$	464.09629	5.556	463.08902	7.742×10^{7}	7.681×10^{7}	7.941×10^{7}	1.492×10^{8}	2.627×10^{7}	1.678×10^{8}	1.455×10^{8}	Negative
220	Ethyl chrysanthemumate	$C_{12}H_{20}O_2$	196.14647	5.759	241.14467	7.123×10^{7}	7.221×10^{7}	1.296×10^{8}	8.409×10^{7}	1.602×10^{8}	8.989×10^{7}	1.046×10^{8}	Negative
221	3-(4-hydroxy-3-methoxyphenyl)propanoic acid	$C_{10}H_{12}O_4$	178.06339	5.376	179.07082	9.494×10^{7}	9.443×10^{7}	1.037×10^{8}	1.211×10^{8}	1.314×10^{8}	7.612×10^{7}	8.352×10^{7}	Positive
222	Porphobilinogen	$C_{10}H_{14}N_2O_4$	226.09489	5.498	225.08761	8.206×10^{7}	7.267×10^{7}	7.739×10^{7}	8.924×10^{7}	1.833×10^{8}	8.769×10^{7}	1.126×10^{8}	Negative
223	Isohomovanillic acid	$C_9H_{10}O_4$	182.05823	0.734	183.06554	1.354×10^{8}	1.292×10^{8}	1.000×10^{8}	9.865×10^{7}	8.626×10^{7}	7.681×10^{7}	7.497×10^{7}	Positive
224	Corchorifatty acid F	$C_{18}H_{32}O_5$	328.22545	6.191	327.21818	6.566×10^{7}	5.037×10^{7}	2.037×10^{8}	6.165×10^{7}	2.156×10^{8}	4.856×10^{7}	5.550×10^{7}	Negative

225	4-((5-(4-Nitrophenyl)oxazol-2-yl)amino)benzonitrile	$C_{16}H_{10}N_4O_3$	306.07766	5.334	305.07039	2.691×10^{8}	2.687×10^{8}	1.915×10^{7}	8.580×10^{7}	1.844×10^{7}	2.500×10^{7}	1.404×10^{7}	Negative
226	Gentisic acid	$C_7H_6O_4$	154.0268	5.563	153.01952	8.102×10^{7}	7.758×10^{7}	1.132×10^{8}	7.620×10^{7}	2.781×10^{8}	3.611×10^{7}	3.610×10^{7}	Negative
227	N-Acetylvaline	$C_7H_{13}NO_3$	159.08973	1.478	160.09699	1.025×10^{8}	1.337×10^{8}	8.506×10^{7}	1.216×10^{8}	8.827×10^{7}	7.059×10^{7}	9.619×10^{7}	Positive
228	2-Anisic acid	$C_8H_8O_3$	152.04763	5.703	153.05492	8.434×10^{7}	1.167×10^{8}	8.832×10^{7}	9.142×10^{7}	2.166×10^{8}	5.722×10^{7}	4.194×10^{7}	Positive
229	N-cyclooctylurea	$C_9H_{18}N_2O$	170.14185	5.94	171.14935	7.507×10^{7}	1.199×10^{8}	9.421×10^{7}	8.664×10^{7}	1.002×10^{8}	1.278×10^{8}	8.883×10^{7}	Positive
230	16-Hydroxyhexadecanoic acid	$C_{16}H_{32}O_3$	272.23517	7.875	271.22789	1.203×10^{8}	1.058×10^{8}	8.892×10^{7}	1.052×10^{8}	1.282×10^{8}	7.706×10^{7}	6.570×10^{7}	Negative
231	N-Acetyl-L-tyrosine	$C_{11}H_{13}NO_4$	223.08476	5.308	222.07748	1.783×10^{8}	1.826×10^{8}	2.519×10^{7}	2.197×10^{8}	2.952×10^{7}	2.831×10^{7}	2.631×10^{7}	Negative
232	Ecgonine	$C_9H_{15}NO_3$	185.10551	5.715	186.11277	3.464×10^{7}	3.590×10^{7}	1.879×10^{8}	5.928×10^{7}	2.074×10^{8}	8.054×10^{7}	7.897×10^{7}	Positive
233	Acetylcarnitine	$C_9H_{17}NO_4$	203.11605	5.717	186.11278	3.489×10^{7}	3.590×10^{7}	1.879×10^{8}	5.928×10^{7}	2.065×10^{8}	8.054×10^{7}	7.899×10^{7}	Positive
234	4-Phenylbutyric acid	$C_{10}H_{12}O_2$	181.11057	5.452	182.11782	3.927×10^{7}	2.190×10^{7}	1.592×10^{8}	6.025×10^{7}	9.543×10^{7}	1.149×10^{8}	1.892×10^{8}	Positive
235	Mevalonic acid	$C_6H_{12}O_4$	148.07375	4.899	147.06647	6.096×10^{7}	7.724×10^{7}	6.804×10^{7}	5.540×10^{7}	8.308×10^{7}	2.413×10^{8}	8.260×10^{7}	Negative
236	N-Acetyl-D-alloisoleucine	$C_8H_{15}NO_3$	173.10541	5.515	172.09814	1.547×10^{8}	1.114×10^{8}	4.995×10^{7}	8.428×10^{7}	6.088×10^{7}	1.139×10^{8}	8.696×10^{7}	Negative
237	1-[4-hydroxy-3-(3-methylbut-2-en-1-yl)phenyl]ethan-1-one	$C_{13}H_{16}O_2$	204.11535	5.753	205.12266	6.790×10^{7}	6.643×10^{7}	1.284×10^{8}	9.751×10^{7}	9.876×10^{7}	9.517×10^{7}	1.071×10^{8}	Positive
238	N(6)-OH-Me-Adenosine	$C_{11}H_{15}N_5O_5$	297.10665	1.861	298.11392	1.280×10^{8}	1.215×10^{8}	5.348×10^{7}	1.280×10^{8}	5.249×10^{7}	4.966×10^{7}	1.227×10^{8}	Positive
239	*D*-glutamine	$C_5H_{10}N_2O_3$	146.06921	1.434	147.07648	1.756×10^{8}	1.255×10^{8}	4.867×10^{7}	1.810×10^{8}	3.364×10^{7}	6.359×10^{7}	2.208×10^{7}	Positive
240	3-(propan-2-yl)-octahydropyrrolo[1,2-a]pyrazine-1,4-dione	$C_{10}H_{16}N_2O_2$	196.12153	5.871	197.1288	7.497×10^{7}	1.205×10^{8}	1.073×10^{8}	7.928×10^{7}	1.050×10^{8}	8.822×10^{7}	7.389×10^{7}	Positive
241	Methyl jasmonate	$C_{13}H_{20}O_3$	224.14152	5.491	207.13827	8.827×10^{7}	8.988×10^{7}	7.159×10^{7}	1.099×10^{8}	1.065×10^{8}	8.775×10^{7}	8.598×10^{7}	Positive
242	Phosphocholine	$C_5H_{14}NO_4P$	145.11031	1.281	184.07347	5.414×10^{7}	7.053×10^{7}	1.600×10^{8}	5.852×10^{7}	1.339×10^{8}	9.154×10^{7}	6.532×10^{7}	Positive
243	5-Hydroxytryptophan	$C_{11}H_{12}N_2O_3$	220.08506	5.062	221.09236	4.788×10^{7}	4.457×10^{7}	1.360×10^{8}	7.844×10^{7}	9.796×10^{7}	1.007×10^{8}	1.254×10^{8}	Positive
244	Gly-Tyr	$C_{11}H_{14}N_2O_4$	238.0957	5.062	221.09242	4.788×10^{7}	4.457×10^{7}	1.360×10^{8}	7.844×10^{7}	9.796×10^{7}	1.007×10^{8}	1.254×10^{8}	Positive
245	Bialaphos	$C_{11}H_{22}N_3O_6P$	323.12175	1.438	324.12901	9.432×10^{7}	9.660×10^{7}	9.377×10^{7}	9.529×10^{7}	8.857×10^{7}	6.882×10^{7}	9.168×10^{7}	Positive
246	Malonic acid	$C_3H_4O_4$	104.01106	1.656	103.00375	8.353×10^{7}	8.735×10^{7}	9.045×10^{7}	8.592×10^{7}	7.723×10^{7}	7.068×10^{7}	1.239×10^{8}	Negative
247	Vanillyl alcohol	$C_8H_{10}O_3$	154.0631	5.127	153.05582	1.054×10^{8}	9.165×10^{7}	9.359×10^{7}	1.150×10^{8}	8.903×10^{7}	6.270×10^{7}	5.799×10^{7}	Negative
248	Coniferin	$C_{16}H_{22}O_8$	359.15811	5.455	360.16537	1.182×10^{8}	1.042×10^{8}	4.235×10^{7}	1.418×10^{8}	1.732×10^{8}	1.680×10^{7}	1.480×10^{7}	Positive
249	Dibenzoyl Thiamine	$C_{26}H_{26}N_4O_4S$	490.16905	5.503	489.16178	5.112×10^{7}	5.494×10^{7}	1.258×10^{8}	7.810×10^{7}	9.049×10^{7}	9.866×10^{7}	1.101×10^{8}	Negative
250	10-Undecenoic acid	$C_{11}H_{20}O_2$	184.14629	5.624	229.14448	4.474×10^{7}	4.438×10^{7}	1.179×10^{8}	6.746×10^{7}	1.357×10^{8}	9.322×10^{7}	1.003×10^{8}	Negative
251	Nonanoic acid	$C_9H_{18}O_2$	158.13081	6.059	139.11299	1.246×10^{8}	9.774×10^{7}	4.002×10^{7}	8.391×10^{7}	6.545×10^{7}	1.299×10^{8}	6.206×10^{7}	Negative
252	Coniferyl alcohol	$C_{10}H_{12}O_3$	180.08294	5.384	181.09022	8.871×10^{7}	9.895×10^{7}	8.129×10^{7}	8.962×10^{7}	8.275×10^{7}	7.915×10^{7}	8.248×10^{7}	Positive
253	Sorbic acid	$C_6H_8O_2$	112.05117	5.214	225.10986	7.437×10^{7}	8.372×10^{7}	5.649×10^{7}	9.800×10^{7}	6.669×10^{7}	1.157×10^{8}	9.764×10^{7}	Positive
254	Lactobionic acid	$C_{12}H_{22}O_{12}$	358.11091	1.311	376.14467	5.985×10^{7}	5.990×10^{7}	7.955×10^{7}	6.856×10^{7}	8.891×10^{7}	1.322×10^{8}	1.020×10^{8}	Positive
255	*L*-Kynurenine	$C_{10}H_{12}N_2O_3$	208.08512	4.866	209.09241	6.236×10^{7}	4.011×10^{7}	7.760×10^{7}	1.159×10^{8}	1.167×10^{8}	1.040×10^{8}	6.848×10^{7}	Positive
256	Maltotetraose	$C_{24}H_{42}O_{21}$	688.20398	5.95	689.21126	1.453×10^{8}	1.332×10^{8}	2.642×10^{7}	1.518×10^{8}	2.599×10^{7}	4.491×10^{7}	5.009×10^{7}	Positive

257	Glucose 1-phosphate	$C_6H_{13}O_9P$	260.02968	1.278	261.03696	7.492×10^{7}	9.226×10^{7}	7.017×10^{7}	6.575×10^{7}	5.476×10^{7}	1.052×10^{8}	1.090×10^{8}	Positive
258	2-Hydroxyphenylacetic acid	$C_8H_8O_3$	152.04753	5.581	151.04024	6.610×10^{7}	6.400×10^{7}	8.462×10^{7}	7.351×10^{7}	1.729×10^{8}	4.663×10^{7}	5.491×10^{7}	Negative
259	*L*-Carnitine	$C_7H_{15}NO_3$	161.10545	1.441	162.11263	6.108×10^{7}	5.667×10^{7}	9.833×10^{7}	5.867×10^{7}	1.993×10^{8}	4.229×10^{7}	4.538×10^{7}	Positive
260	2-Phenylethylamine	$C_8H_{11}N$	121.08934	5.376	166.08755	3.566×10^{7}	3.833×10^{7}	9.720×10^{7}	6.110×10^{7}	9.809×10^{7}	1.143×10^{8}	1.132×10^{8}	Negative
261	N1-(1H-1,2,4-triazol-3-yl)-2-hydroxybenzamide	$C_9H_8N_4O_2$	204.06368	0.585	205.07095	8.537×10^{7}	9.007×10^{7}	7.816×10^{7}	8.528×10^{7}	6.563×10^{7}	7.515×10^{7}	7.501×10^{7}	Positive
262	Chrysin	$C_{15}H_{10}O_4$	254.05801	6.684	253.05073	7.896×10^{6}	6.067×10^{6}	2.637×10^{8}	1.848×10^{7}	2.410×10^{8}	4.648×10^{6}	1.261×10^{7}	Negative
263	*L*-Malate	$C_4H_6O_5$	134.02164	4.996	133.01436	1.319×10^{8}	1.190×10^{8}	4.776×10^{7}	1.290×10^{8}	4.552×10^{7}	3.722×10^{7}	3.339×10^{7}	Negative
264	2-hydroxy-6-[(8Z,11Z)-pentadeca-8,11,14-trien-1-yl]benzoic acid	$C_{22}H_{30}O_3$	324.20408	10.105	325.21136	7.146×10^{7}	9.363×10^{7}	9.499×10^{7}	6.257×10^{7}	1.124×10^{8}	5.196×10^{7}	4.973×10^{7}	Positive
265	Norepinephrine	$C_8H_{11}NO_3$	169.07415	5.715	168.0668	2.254×10^{7}	2.365×10^{7}	1.190×10^{8}	4.120×10^{7}	1.497×10^{8}	1.153×10^{8}	6.164×10^{7}	Negative
266	Pyridoxamine	$C_8H_{12}N_2O_2$	168.08963	5.644	381.17743	1.145×10^{8}	1.150×10^{8}	3.796×10^{7}	8.377×10^{7}	3.264×10^{7}	8.538×10^{7}	5.993×10^{7}	Negative
267	2-Pyridylacetic acid	$C_7H_7NO_2$	137.04794	5.385	138.05521	5.425×10^{7}	5.189×10^{7}	2.026×10^{8}	9.641×10^{7}	4.784×10^{7}	3.212×10^{7}	3.895×10^{7}	Positive
268	Undecanedioic acid	$C_{11}H_{20}O_4$	216.13637	6.072	215.12909	1.014×10^{8}	9.684×10^{7}	3.836×10^{7}	8.212×10^{7}	6.155×10^{7}	9.105×10^{7}	4.986×10^{7}	Negative
269	alpha-D-Glucopyranosyl 2-O-(2-methylbutanoyl)-alpha-D-glucopyranoside	$C_{17}H_{30}O_{12}$	472.1797	5.459	471.17242	1.767×10^{8}	2.259×10^{8}	2.355×10^{7}	6.122×10^{7}	4.217×10^{6}	2.197×10^{7}	6.669×10^{6}	Negative
270	Oxaceprol	$C_7H_{11}NO_4$	173.06905	1.567	174.07635	1.113×10^{8}	7.939×10^{7}	5.468×10^{7}	1.198×10^{8}	4.484×10^{7}	5.515×10^{7}	5.300×10^{7}	Positive
271	Guanine	$C_5H_5N_5O$	151.04956	3.763	152.05684	1.045×10^{8}	9.327×10^{7}	4.570×10^{7}	8.332×10^{7}	6.016×10^{7}	8.436×10^{7}	4.492×10^{7}	Positive
272	5-amino-1-phenyl-1H-pyrazole-4-carbonitrile	$C_{10}H_8N_4$	184.07374	5.77	183.06646	4.800×10^{7}	4.387×10^{7}	9.324×10^{7}	4.908×10^{7}	1.856×10^{8}	5.496×10^{7}	3.998×10^{7}	Negative
273	3-[4-methyl-1-(2-methylpropanoyl)-3-oxocyclohexyl]butanoic acid	$C_{15}H_{24}O_4$	268.16758	5.743	267.16031	6.333×10^{7}	6.432×10^{7}	2.336×10^{8}	1.203×10^{8}	1.155×10^{7}	7.691×10^{6}	1.051×10^{7}	Negative
274	Uric Acid	$C_5H_4N_4O_3$	168.02846	1.934	167.02118	9.607×10^{7}	1.049×10^{8}	5.197×10^{7}	1.319×10^{8}	3.367×10^{7}	6.583×10^{7}	2.564×10^{7}	Negative
275	N-acetyl-D-glucosamine	$C_8H_{15}NO_6$	221.09005	1.566	222.09733	6.971×10^{7}	6.511×10^{7}	5.146×10^{7}	1.820×10^{8}	5.396×10^{7}	3.453×10^{7}	4.944×10^{7}	Positive
276	AL 8810 Methyl ester	$C_{25}H_{33}FO_4$	438.22331	6.023	439.23059	1.911×10^{8}	1.304×10^{8}	2.333×10^{7}	6.383×10^{7}	4.384×10^{7}	3.526×10^{7}	9.837×10^{6}	Positive
277	N'1-phenylpropanohydrazide	$C_9H_{12}N_2O$	164.09513	5.358	163.08786	8.862×10^{5}	8.597×10^{5}	9.598×10^{5}	8.255×10^{5}	1.421×10^{6}	8.247×10^{5}	4.899×10^{8}	Negative
278	Myricetin	$C_{15}H_{10}O_8$	318.03798	5.68	317.0307	5.480×10^{7}	4.625×10^{7}	2.184×10^{8}	1.454×10^{8}	1.251×10^{7}	8.715×10^{6}	9.458×10^{6}	Negative
279	Paracetamol	$C_8H_9NO_2$	151.06354	5.469	152.07081	2.791×10^{7}	3.312×10^{7}	8.364×10^{7}	3.013×10^{7}	2.332×10^{8}	3.930×10^{7}	4.806×10^{7}	Positive
280	*L*-Threonine	$C_4H_9NO_3$	119.05853	1.294	120.0658	1.164×10^{8}	1.032×10^{8}	2.671×10^{7}	1.580×10^{8}	3.041×10^{7}	2.931×10^{7}	2.372×10^{7}	Positive
281	Tropine	$C_8H_{15}NO$	141.11556	9.432	142.12284	7.573×10^{7}	7.966×10^{7}	6.453×10^{7}	6.306×10^{7}	7.595×10^{7}	6.651×10^{7}	6.213×10^{7}	Positive
282	Dihydroroseoside	$C_{19}H_{32}O_8$	388.21062	5.562	433.20887	6.680×10^{7}	6.241×10^{7}	7.045×10^{7}	6.950×10^{7}	7.693×10^{7}	6.510×10^{7}	7.577×10^{7}	Negative
283	3-[3,5-bis(trifluoromethyl)phenyl]-N'-hydroxyprop-2-enimidamide	$C_{11}H_8F_6N_2O$	242.08741	7.792	281.05057	7.998×10^{7}	6.883×10^{7}	8.276×10^{7}	8.168×10^{7}	3.731×10^{7}	7.022×10^{7}	6.519×10^{7}	Positive
284	4-Methylaminoantipyrine	$C_{12}H_{15}N_3O$	217.12181	5.131	218.12909	9.120×10^{5}	8.809×10^{5}	8.766×10^{5}	1.110×10^{6}	1.900×10^{6}	1.446×10^{6}	4.713×10^{8}	Positive

285	2-Phenylglycine	$C_8H_9NO_2$	151.0634	5.149	150.05612	3.284×10^7	3.028×10^7	1.271×10^8	4.488×10^7	7.063×10^7	2.944×10^7	1.412×10^8	Negative
286	5-Methoxyindole-3-Carbaldehyde	$C_{10}H_9NO_2$	175.06349	5.342	174.05621	1.290×10^8	1.167×10^8	1.731×10^7	1.612×10^8	1.745×10^7	1.516×10^7	1.867×10^7	Negative
287	2,5-Dihydroxybenzaldehyde	$C_7H_6O_3$	138.03183	5.549	137.02453	9.526×10^7	8.801×10^7	5.307×10^7	1.144×10^8	6.958×10^7	2.630×10^7	2.880×10^7	Negative
288	2-Arachidonyl Glycerol ether	$C_{23}H_{40}O_3$	364.29549	10.717	365.30276	9.117×10^7	9.923×10^7	2.686×10^7	7.734×10^7	1.518×10^7	7.295×10^7	9.037×10^7	Positive
289	Methyl dihydrojasmonate	$C_{13}H_{22}O_3$	226.15711	6.002	227.16445	8.195×10^7	6.512×10^7	6.048×10^7	7.277×10^7	5.662×10^7	7.395×10^7	5.112×10^7	Positive
290	Traumatic acid	$C_{12}H_{20}O_4$	228.1364	6.003	455.26562	5.666×10^7	4.708×10^7	2.795×10^7	5.775×10^7	3.323×10^7	1.648×10^8	7.230×10^7	Negative
291	Chlorogenic acid	$C_{16}H_{18}O_9$	354.09571	5.348	353.08843	4.209×10^7	4.256×10^7	8.198×10^7	6.643×10^7	6.434×10^7	7.734×10^7	8.256×10^7	Negative
292	*DL*-3,4-Dihydroxyphenyl glycol	$C_8H_{10}O_4$	170.05811	5.583	169.05084	8.267×10^7	7.623×10^7	6.200×10^7	5.779×10^7	9.286×10^7	4.701×10^7	3.599×10^7	Negative
293	3-hydroxy-2-octylpentanedioic acid	$C_{13}H_{24}O_5$	242.15198	5.495	243.15916	7.091×10^7	6.194×10^7	5.258×10^7	7.710×10^7	7.102×10^7	6.823×10^7	4.616×10^7	Positive
294	6-(3-hydroxybutan-2-yl)-5-(hydroxymethyl)-4-methoxy-2H-pyran-2-one	$C_{11}H_{16}O_5$	188.10518	5.036	211.09441	7.107×10^7	8.499×10^7	1.353×10^8	1.511×10^8	1.164×10^6	2.504×10^6	1.647×10^6	Positive
295	2,2-dimethyl-6,7-di[(4-nitrobenzyl)oxy]chroman-4-one	$C_{25}H_{22}N_2O_8$	500.11898	9.432	501.12626	8.137×10^7	6.273×10^7	8.218×10^7	6.949×10^7	2.676×10^7	6.196×10^7	5.493×10^7	Positive
296	*o*-Veratraldehyde	$C_9H_{10}O_3$	166.06325	5.997	167.07059	4.049×10^7	4.374×10^7	5.811×10^7	5.176×10^7	8.299×10^7	7.512×10^7	8.427×10^7	Positive
297	6,7-Dihydroxycoumarin	$C_9H_6O_4$	178.02696	5.393	177.01968	6.840×10^7	7.366×10^7	3.141×10^7	1.037×10^8	3.576×10^7	7.322×10^7	4.870×10^7	Negative
298	Azelaic acid	$C_9H_{16}O_4$	142.09954	5.574	141.09231	5.165×10^7	5.094×10^7	4.987×10^7	6.771×10^7	6.051×10^7	7.290×10^7	7.894×10^7	Negative
299	Guvacoline	$C_7H_{11}NO_2$	141.07928	4.855	142.08656	4.537×10^7	4.779×10^7	1.891×10^8	9.818×10^7	1.703×10^7	1.637×10^7	1.468×10^7	Positive
300	*L*-Ascorbate	$C_6H_8O_6$	176.03228	1.465	175.02507	6.652×10^7	7.027×10^7	5.697×10^7	6.607×10^7	5.940×10^7	4.770×10^7	6.009×10^7	Negative
301	3',4'-Dihydroxyphenylacetone	$C_9H_{10}O_3$	166.06333	5.342	167.07062	4.845×10^7	2.337×10^7	7.389×10^7	4.435×10^7	1.472×10^8	3.890×10^7	4.397×10^7	Positive
302	2-{[3-(3,4,5-trimethoxyphenyl)propanoyl]amino}acetic acid	$C_{14}H_{19}NO_6$	297.12163	5.425	296.11429	3.680×10^7	3.764×10^7	9.476×10^7	5.304×10^7	6.329×10^7	5.449×10^7	7.680×10^7	Negative
303	Xanthosine	$C_{10}H_{12}N_4O_6$	284.07579	4.771	283.06852	8.005×10^7	8.612×10^7	2.122×10^7	8.026×10^7	8.106×10^7	3.612×10^7	2.712×10^7	Negative
304	Levodopa	$C_9H_{11}NO_4$	197.06902	5.925	178.05118	2.671×10^7	2.695×10^7	9.656×10^7	5.465×10^7	1.215×10^8	3.612×10^7	4.746×10^7	Negative
305	3-Phenyllactic acid	$C_9H_{10}O_3$	120.05762	5.734	119.05029	6.826×10^7	6.217×10^7	4.822×10^7	6.928×10^7	3.656×10^7	3.526×10^7	8.723×10^7	Negative
306	4-Hydroxy-3-methylbenzoic acid	$C_8H_8O_3$	152.04756	5.422	151.04029	6.558×10^7	5.559×10^7	6.077×10^7	7.142×10^7	6.147×10^7	3.265×10^7	5.643×10^7	Negative
307	2-methyl-1,2-dihydrophthalazin-1-one	$C_9H_8N_2O$	160.06388	5.773	161.07118	5.873×10^7	6.154×10^7	6.001×10^7	7.156×10^7	6.135×10^7	3.661×10^7	5.122×10^7	Positive
308	(5E)-7-methylidene-10-oxo-4-(propan-2-yl)undec-5-enoic acid	$C_{15}H_{24}O_3$	118.09929	6.316	275.16168	7.644×10^7	7.786×10^7	4.435×10^7	6.340×10^7	4.039×10^7	6.089×10^7	3.711×10^7	Positive
309	5-Hydroxytryptophol	$C_{10}H_{11}NO_2$	177.07923	5.76	178.08655	2.679×10^7	3.475×10^7	9.183×10^7	4.621×10^7	1.240×10^8	3.895×10^7	3.712×10^7	Positive
310	N6-Me-Adenosine	$C_{11}H_{15}N_5O_4$	281.1111	1.49	282.11836	4.593×10^7	4.413×10^7	7.482×10^7	6.103×10^7	6.210×10^7	4.275×10^7	6.540×10^7	Positive
311	Methyl nicotinate	$C_7H_7NO_2$	137.04793	5.522	138.0552	1.703×10^7	1.696×10^7	8.780×10^7	4.720×10^7	1.685×10^8	2.563×10^7	2.973×10^7	Positive
312	Methionine sulfoxide	$C_5H_{11}NO_3S$	165.04614	1.377	166.05342	8.274×10^7	8.759×10^7	1.909×10^7	1.035×10^8	3.187×10^7	3.766×10^7	2.883×10^7	Positive
313	Hydroquinone	$C_6H_6O_2$	110.03692	5.43	109.02964	4.622×10^7	3.999×10^7	6.705×10^7	4.566×10^7	1.220×10^8	3.691×10^7	3.182×10^7	Negative
314	Sucrose	$C_{12}H_{22}O_{11}$	342.1167	0.511	341.10937	5.210×10^7	5.403×10^7	4.442×10^7	4.118×10^7	4.178×10^7	6.719×10^7	8.775×10^7	Negative

315	Spermidine	$C_7H_{19}N_3$	145.15796	1.07	146.16524	1.082×10^{8}	1.077×10^{8}	2.263×10^{7}	6.820×10^{7}	2.332×10^{7}	4.081×10^{7}	1.515×10^{7}	Positive
316	N-Acetylglycine	$C_4H_7NO_3$	117.04303	1.4	118.05021	3.589×10^{7}	2.758×10^{7}	7.688×10^{7}	4.164×10^{7}	6.275×10^{7}	7.353×10^{7}	6.163×10^{7}	Positive
317	2-Isopropylmalate	$C_7H_{12}O_5$	176.06879	11.978	159.06552	5.080×10^{7}	6.314×10^{7}	5.115×10^{7}	5.869×10^{7}	5.386×10^{7}	5.242×10^{7}	4.978×10^{7}	Positive
318	Spiculisporic Acid	$C_{17}H_{28}O_6$	328.18921	5.93	373.18741	7.812×10^{7}	7.259×10^{7}	5.721×10^{7}	6.743×10^{7}	5.387×10^{7}	3.844×10^{7}	1.138×10^{7}	Negative
319	Royal jelly acid	$C_{10}H_{18}O_3$	186.1258	5.552	185.11852	4.192×10^{7}	4.188×10^{7}	5.709×10^{7}	4.247×10^{7}	1.026×10^{8}	4.478×10^{7}	4.688×10^{7}	Negative
320	1-Methylguanosine	$C_{11}H_{15}N_5O_5$	297.10593	2.121	298.11321	8.625×10^{7}	6.820×10^{7}	2.532×10^{7}	7.808×10^{7}	2.606×10^{7}	2.670×10^{7}	6.352×10^{7}	Positive
321	(12Z)-9,10,11-trihydroxyoctadec-12-enoic acid	$C_{18}H_{34}O_5$	352.22276	6.407	353.22998	2.320×10^{7}	2.277×10^{7}	8.991×10^{7}	2.726×10^{7}	1.717×10^{8}	1.720×10^{7}	1.868×10^{7}	Positive
322	2-Isopropylaniline	$C_9H_{13}N$	153.11555	5.886	136.11227	2.734×10^{7}	2.516×10^{7}	5.892×10^{7}	3.790×10^{7}	4.080×10^{7}	6.994×10^{7}	1.079×10^{8}	Positive
323	5,8-dihydroxy-10-methyl-5,8,9,10-tetrahydro-2H-oxecin-2-one	$C_{10}H_{14}O_4$	180.07882	5.352	181.0861	4.609×10^{7}	4.415×10^{7}	6.842×10^{7}	5.685×10^{7}	9.056×10^{7}	3.128×10^{7}	2.863×10^{7}	Positive
324	*L*-(-)-alpha-Amino-epsilon-Caprolactam	$C_6H_{12}N_2O$	128.09536	5.451	295.1539	4.951×10^{7}	5.599×10^{7}	5.906×10^{7}	5.063×10^{7}	6.188×10^{7}	3.078×10^{7}	5.575×10^{7}	Positive
325	*L*-serine	$C_3H_7NO_3$	105.04297	1.271	106.05025	8.059×10^{7}	8.501×10^{7}	2.807×10^{7}	1.011×10^{8}	2.112×10^{7}	2.420×10^{7}	2.205×10^{7}	Positive
326	N'3-(3,4,5-trimethoxybenzylidene)pyridine-3-carbohydrazide	$C_{16}H_{17}N_3O_4$	337.10095	1.428	338.10823	3.134×10^{7}	3.082×10^{7}	5.081×10^{7}	5.400×10^{7}	4.950×10^{7}	7.324×10^{7}	7.235×10^{7}	Positive
327	4'-Methoxyacetophenone	$C_9H_{10}O_2$	150.06829	5.281	151.07556	2.183×10^{7}	2.081×10^{7}	4.227×10^{7}	2.507×10^{7}	2.181×10^{8}	1.413×10^{7}	1.973×10^{7}	Positive
328	Acetylcholine	$C_7H_{15}NO_2$	145.1103	1.446	146.11758	9.566×10^{6}	1.200×10^{7}	9.157×10^{7}	1.109×10^{7}	2.113×10^{8}	8.366×10^{6}	1.689×10^{7}	Positive
329	2,4-Dihydroxybenzoic acid	$C_7H_6O_4$	154.02681	5.794	153.01953	3.954×10^{7}	4.102×10^{7}	5.878×10^{7}	3.995×10^{7}	1.065×10^{8}	3.215×10^{7}	3.435×10^{7}	Negative
330	Linustatin	$C_{16}H_{27}NO_{11}$	426.18703	5.695	427.19429	3.027×10^{7}	3.236×10^{7}	3.612×10^{7}	4.776×10^{7}	2.283×10^{7}	6.453×10^{7}	1.179×10^{8}	Positive
331	2-(3,4-dimethoxyphenyl)ethanamine	$C_{10}H_{15}NO_2$	181.11043	6.039	180.10315	4.687×10^{7}	4.313×10^{7}	2.726×10^{7}	5.050×10^{7}	6.355×10^{7}	8.561×10^{7}	3.444×10^{7}	Negative
332	N1-(1,3-thiazol-2-yl)-2-chlorobenzamide	$C_{10}H_7ClN_2OS$	237.99583	0.591	239.00308	5.288×10^{7}	5.663×10^{7}	5.094×10^{7}	5.523×10^{7}	5.183×10^{7}	4.770×10^{7}	3.573×10^{7}	Positive
333	Perillic acid	$C_{10}H_{14}O_2$	166.09951	6.244	165.09223	2.958×10^{7}	2.941×10^{7}	6.286×10^{7}	4.455×10^{7}	9.659×10^{7}	4.561×10^{7}	3.763×10^{7}	Negative
334	Eicosapentaenoic acid ethyl ester	$C_{22}H_{34}O_2$	347.28016	8.4	348.28744	6.647×10^{7}	4.564×10^{7}	3.450×10^{7}	4.517×10^{7}	5.103×10^{7}	6.408×10^{7}	3.824×10^{7}	Positive
335	(5-L-Glutamyl)-L-Amino Acid	$C_8H_{14}N_2O_5$	218.09056	2.261	219.09784	8.342×10^{6}	8.893×10^{6}	3.995×10^{7}	5.953×10^{7}	4.312×10^{6}	9.499×10^{6}	2.068×10^{8}	Positive
336	6-anilino-1,3-dimethyl-1,2,3,4-tetrahydropyrimidine-2,4-dione	$C_{12}H_{13}N_3O_2$	231.10497	7.307	232.11225	5.933×10^{5}	6.400×10^{5}	2.255×10^{8}	1.230×10^{6}	8.914×10^{7}	6.174×10^{5}	1.838×10^{7}	Positive
337	THJ2201 N-(5-hydroxypentyl) metabolite	$C_{23}H_{22}N_2O_2$	358.17405	5.538	359.18134	1.082×10^{7}	1.182×10^{7}	1.029×10^{8}	1.469×10^{7}	2.182×10^{7}	4.060×10^{7}	1.325×10^{8}	Positive
338	Homogentisic Acid	$C_8H_8O_4$	168.04248	5.411	167.03521	2.859×10^{7}	2.778×10^{7}	5.603×10^{7}	2.787×10^{7}	7.638×10^{7}	1.031×10^{8}	1.337×10^{7}	Negative
339	ethyl 4-amino-2-(methylsulfanyl)-1,3-thiazole-5-carboxylate	$C_7H_{10}N_2O_2S_2$	218.01959	6.157	219.02687	7.457×10^{7}	7.087×10^{7}	3.267×10^{7}	6.253×10^{7}	4.435×10^{7}	2.201×10^{7}	2.580×10^{7}	Positive
340	4-decyl-3-hydroxy-5-oxooxolane-2,3-dicarboxylic acid	$C_{16}H_{26}O_7$	352.15031	5.647	353.15743	4.964×10^{7}	4.328×10^{7}	4.246×10^{7}	5.140×10^{7}	5.117×10^{7}	3.510×10^{7}	5.219×10^{7}	Positive
341	KPH	$C_{17}H_{28}N_6O_4$	362.20674	5.752	363.21402	4.821×10^{7}	4.391×10^{7}	4.858×10^{7}	5.661×10^{7}	7.765×10^{7}	2.505×10^{7}	2.427×10^{7}	Positive
342	Adrenosterone	$C_{19}H_{24}O_3$	300.17218	6.118	318.20612	2.586×10^{7}	2.680×10^{7}	6.172×10^{7}	3.945×10^{7}	1.142×10^{8}	2.810×10^{7}	2.649×10^{7}	Positive

343	(2R)-2-[(2R,5S)-5-[(2S)-2-hydroxybutyl]oxolan-2-yl]propanoic acid	$C_{11}H_{20}O_4$	216.13646	6.073	217.14374	6.524×10^7	7.406×10^7	1.866×10^7	5.551×10^7	2.976×10^7	4.658×10^7	2.950×10^7	Positive
344	4-(2-((4-Cyanophenyl)amino)oxazol-5-yl)benzonitrile	$C_{17}H_{10}N_4O$	286.08772	5.363	285.08044	1.006×10^8	9.953×10^7	3.182×10^6	1.098×10^8	2.545×10^6	1.334×10^6	1.691×10^6	Negative
345	1-(5H-dibenzo[b,f]azepin-5-yl)ethan-1-one	$C_{16}H_{13}NO$	219.12583	6.402	258.08906	4.742×10^6	5.492×10^6	9.709×10^7	1.315×10^7	5.096×10^7	9.883×10^7	4.818×10^7	Positive
346	Choline bitartrate	$C_9H_{19}NO_7$	253.1164	1.454	254.12372	4.285×10^7	5.346×10^7	5.482×10^7	3.928×10^7	3.956×10^7	3.827×10^7	5.013×10^7	Positive
347	3-Methoxytyramine	$C_9H_{13}NO_2$	167.09475	5.887	166.08748	1.071×10^7	1.011×10^7	1.016×10^8	2.205×10^7	2.175×10^7	6.433×10^7	8.361×10^7	Negative
348	9-(3-O-Methylpentofuranosyl)-1,9-dihydro-6H-purin-6-one	$C_{11}H_{14}N_4O_5$	282.0951	1.432	281.08762	3.387×10^7	3.592×10^7	7.126×10^7	3.672×10^7	5.513×10^7	2.728×10^7	5.252×10^7	Negative
349	Cyclohexaneacetic acid	$C_8H_{14}O_2$	142.09958	5.793	141.09228	2.625×10^7	3.306×10^7	5.673×10^7	3.503×10^7	9.302×10^7	3.478×10^7	3.328×10^7	Negative
350	2-(2,6-dihydroxyphenyl)-3,5,7-trihydroxy-4H-chromen-4-one	$C_{15}H_{10}O_7$	302.04266	5.386	303.04993	7.598×10^7	5.955×10^7	5.080×10^7	7.828×10^7	1.267×10^7	2.285×10^7	1.014×10^7	Positive
351	*L*-Cycloserine	$C_3H_6N_2O_2$	102.0434	3.773	103.05068	2.088×10^7	1.928×10^7	4.406×10^7	2.461×10^7	3.381×10^7	3.112×10^7	1.358×10^8	Positive
352	Xanthurenic Acid	$C_{10}H_7NO_4$	205.03771	5.572	204.03043	7.090×10^7	3.306×10^7	5.721×10^7	1.785×10^7	4.973×10^7	2.349×10^7	5.666×10^7	Negative
353	3-Indoleacetonitrile	$C_{10}H_8N_2$	156.069	6.03	157.07627	1.367×10^6	1.164×10^6	2.446×10^8	2.704×10^6	2.113×10^7	1.187×10^6	3.640×10^7	Positive
354	Prostaglandin E1	$C_{20}H_{34}O_5$	354.23947	5.775	389.20862	1.509×10^7	1.678×10^7	1.061×10^8	3.070×10^7	1.310×10^7	6.142×10^7	6.537×10^7	Negative
355	Sulforidazine	$C_{21}H_{26}N_2O_2S_2$	402.15045	5.451	403.1577	8.064×10^7	8.905×10^7	2.216×10^7	1.954×10^7	2.751×10^7	2.967×10^7	3.821×10^7	Positive
356	Leucylproline	$C_{11}H_{20}N_2O_3$	228.14737	5.224	229.15465	6.257×10^7	4.551×10^7	3.028×10^7	8.610×10^7	2.498×10^7	2.407×10^7	3.285×10^7	Positive
357	2-Methoxybenzaldehyde	$C_8H_8O_2$	136.04981	5.034	137.05708	4.626×10^7	5.069×10^7	3.877×10^7	4.599×10^7	3.573×10^7	3.938×10^7	4.433×10^7	Positive
358	Isoeugenyl acetate	$C_{12}H_{14}O_3$	206.09229	5.686	207.09954	2.801×10^7	4.209×10^7	4.261×10^7	4.540×10^7	4.006×10^7	4.906×10^7	5.042×10^7	Positive
359	2-(2,4-dihydroxyphenyl)-3,5,7-trihydroxy-4H-chromen-4-one	$C_{15}H_{10}O_7$	302.04247	5.544	303.04975	3.989×10^7	3.909×10^7	4.716×10^7	7.708×10^7	8.839×10^6	5.276×10^7	3.128×10^7	Positive
360	N-acetyl-L-ornithine	$C_7H_{14}N_2O_3$	174.10051	1.715	173.09323	4.419×10^7	5.674×10^7	5.992×10^6	4.290×10^7	8.622×10^7	3.035×10^7	2.859×10^7	Negative
361	3-[3-(beta-D-Glucopyranosyloxy)-2-hydroxyphenyl]propanoic acid	$C_{15}H_{20}O_9$	344.11134	5.576	343.10406	2.108×10^7	2.020×10^7	6.744×10^7	2.500×10^7	8.290×10^7	2.819×10^7	4.298×10^7	Negative
362	2-(2-hydroxy-3-methylbutanamido)-4-methylpentanoic acid	$C_{11}H_{21}NO_4$	253.13157	4.775	254.13884	3.522×10^7	3.393×10^7	3.564×10^7	4.862×10^7	4.557×10^7	4.354×10^7	4.369×10^7	Positive
363	3-Hydroxy-3-methylbutanoic acid	$C_5H_{10}O_3$	118.05255	4.777	254.13887	3.522×10^7	3.393×10^7	3.564×10^7	4.862×10^7	4.557×10^7	4.354×10^7	4.369×10^7	Positive
364	N-lactoyl-phenylalanine	$C_{12}H_{15}NO_4$	237.1002	5.027	236.09292	7.537×10^7	4.518×10^7	2.410×10^7	2.628×10^7	3.832×10^7	1.168×10^7	6.523×10^7	Negative
365	JWH 073 N-(4-hydroxybutyl) metabolite	$C_{23}H_{21}NO_2$	163.06342	5.513	164.07082	3.211×10^7	3.286×10^7	4.859×10^7	3.767×10^7	3.553×10^7	4.499×10^7	5.140×10^7	Positive
366	6-Sialyllactose	$C_{23}H_{39}NO_{19}$	633.21379	1.782	632.20652	7.546×10^7	6.947×10^7	8.143×10^6	7.946×10^7	7.373×10^6	1.599×10^7	2.524×10^7	Negative
367	Furanyl fentanyl 3-furancarboxamide isomer-d5	$C_{24}H_{21}[2]H_5N_2O_2$	379.23589	6.237	380.2431	3.441×10^7	3.152×10^7	4.441×10^7	2.278×10^7	4.383×10^7	5.599×10^7	4.746×10^7	Positive
368	2-Methylbutyroylcarnitine	$C_{12}H_{23}NO_4$	245.16277	6.02	244.15549	3.228×10^7	2.958×10^7	1.141×10^7	3.916×10^7	1.153×10^7	1.184×10^8	3.775×10^7	Negative
369	Oxoamide	$C_{10}H_{12}N_2O_2$	192.08986	5.679	193.09746	2.283×10^7	2.796×10^7	5.762×10^7	3.592×10^6	7.138×10^7	5.118×10^7	3.744×10^7	Positive
370	Ornithine	$C_5H_{12}N_2O_2$	132.09022	1.177	133.09742	7.381×10^7	6.814×10^7	2.276×10^7	8.390×10^7	6.630×10^6	1.046×10^7	6.287×10^6	Positive
371	Glycocholic acid	$C_{26}H_{43}NO_6$	465.30674	9.986	466.31402	4.379×10^7	4.290×10^7	3.080×10^7	4.249×10^7	4.010×10^7	3.647×10^7	3.292×10^7	Positive

372	Metanephrine	$C_{10}H_{15}NO_3$	197.1055	5.566	196.09821	1.417×10^{7}	1.911×10^{7}	5.605×10^{7}	3.287×10^{7}	5.856×10^{7}	4.625×10^{7}	4.229×10^{7}	Negative
373	Chalcone	$C_{15}H_{12}O$	208.08905	6.717	209.09634	4.729×10^{6}	4.340×10^{6}	3.268×10^{7}	3.683×10^{6}	2.155×10^{8}	2.052×10^{6}	5.311×10^{6}	Positive
374	2-Hydroxy-2-methyl-3-buten-1-yl beta-D-glucopyranoside	$C_{11}H_{20}O_7$	246.11053	5.462	247.1178	8.475×10^{7}	1.010×10^{8}	1.231×10^{7}	3.168×10^{7}	7.564×10^{6}	1.364×10^{7}	1.287×10^{7}	Positive
375	13-HPODE	$C_{18}H_{32}O_4$	312.22942	7.109	313.2363	3.975×10^{7}	3.797×10^{7}	3.356×10^{7}	4.456×10^{7}	3.615×10^{7}	3.801×10^{7}	3.304×10^{7}	Positive
376	Genistein	$C_{15}H_{10}O_5$	270.05296	6.759	269.04569	7.687×10^{6}	6.540×10^{6}	1.148×10^{8}	1.689×10^{7}	1.014×10^{8}	7.427×10^{6}	5.671×10^{6}	Negative
377	2-Phenylpropionic acid	$C_9H_{10}O_2$	150.06823	5.927	149.0609	2.918×10^{7}	3.082×10^{7}	3.756×10^{7}	4.171×10^{7}	4.833×10^{7}	3.941×10^{7}	3.230×10^{7}	Negative
378	6-Ketoprostaglandin F1α	$C_{20}H_{34}O_6$	370.23594	5.938	369.22867	8.231×10^{7}	7.373×10^{7}	3.166×10^{6}	8.915×10^{7}	5.147×10^{6}	2.226×10^{6}	2.845×10^{6}	Negative
379	Taurine	$C_2H_7NO_3S$	125.0147	1.288	124.00742	4.082×10^{7}	4.662×10^{7}	3.467×10^{7}	3.843×10^{7}	3.464×10^{7}	3.091×10^{7}	3.104×10^{7}	Negative
380	Adenosine 5'-monophosphate	$C_{10}H_{14}N_5O_7P$	347.06334	1.723	346.05606	2.101×10^{7}	2.895×10^{7}	9.012×10^{7}	1.219×10^{7}	7.615×10^{7}	1.972×10^{7}	6.308×10^{6}	Negative
381	Gly-Phe	$C_{11}H_{14}N_2O_3$	222.10059	5.263	203.08287	4.239×10^{7}	4.241×10^{7}	3.975×10^{7}	4.019×10^{7}	3.783×10^{7}	2.758×10^{7}	2.337×10^{7}	Negative
382	*L*-Tryptophan	$C_{11}H_{12}N_2O_2$	204.09012	5.266	203.08285	4.239×10^{7}	4.241×10^{7}	3.975×10^{7}	4.019×10^{7}	3.783×10^{7}	2.758×10^{7}	2.337×10^{7}	Negative
383	methyl 2-[(2-acetyl-3-oxo-1-butenyl)amino]acetate	$C_9H_{13}NO_4$	199.08483	4.834	200.09212	2.364×10^{7}	2.424×10^{7}	2.690×10^{7}	3.096×10^{7}	5.088×10^{7}	5.990×10^{7}	3.671×10^{7}	Positive
384	Uridine 5'-diphosphogalactose	$C_{15}H_{24}N_2O_{17}P_2$	566.05608	1.281	565.04887	3.069×10^{7}	3.065×10^{7}	5.165×10^{7}	2.356×10^{7}	4.671×10^{7}	3.745×10^{7}	2.817×10^{7}	Negative
385	N-Formylkynurenine	$C_{11}H_{12}N_2O_4$	236.07978	4.978	237.0871	3.128×10^{7}	3.289×10^{7}	2.378×10^{7}	8.461×10^{7}	4.335×10^{7}	2.662×10^{7}	5.133×10^{6}	Positive
386	TQH	$C_{15}H_{24}N_6O_6$	384.17691	5.589	385.18353	6.445×10^{7}	5.672×10^{7}	2.015×10^{7}	3.328×10^{7}	2.579×10^{7}	3.230×10^{7}	1.428×10^{7}	Positive
387	Perillartine	$C_{10}H_{15}NO$	165.1156	6.707	166.12288	3.252×10^{6}	2.644×10^{6}	6.612×10^{7}	3.760×10^{6}	1.440×10^{8}	9.407×10^{6}	1.741×10^{7}	Positive
388	N-(4-piperidinophenyl)-2-thiophenecarboxamide	$C_{16}H_{18}N_2OS$	270.14418	5.942	309.10724	2.966×10^{7}	3.189×10^{7}	3.591×10^{7}	4.104×10^{7}	4.503×10^{7}	2.856×10^{7}	2.915×10^{7}	Positive
389	*L*-lysine	$C_6H_{14}N_2O_2$	146.10558	1.261	147.11285	4.886×10^{7}	3.513×10^{7}	2.982×10^{7}	3.002×10^{7}	2.809×10^{7}	3.515×10^{7}	3.032×10^{7}	Positive
390	Stearic acid	$C_{18}H_{36}O_2$	284.27153	10.758	267.26822	3.958×10^{7}	3.991×10^{7}	3.658×10^{7}	2.746×10^{7}	3.443×10^{7}	3.146×10^{7}	2.742×10^{7}	Positive
391	N-(4-butyl-2-methylphenyl)-N'-[4-(4-methylpiperazino)phenyl]urea	$C_{23}H_{32}N_4O$	380.25416	6.069	381.26144	3.155×10^{7}	3.261×10^{7}	2.239×10^{7}	5.202×10^{7}	4.322×10^{7}	3.765×10^{7}	1.661×10^{7}	Positive
392	5-[4-(4-methoxyphenoxy)phenyl]-1H-pyrazole	$C_{16}H_{14}N_2O_2$	266.10576	6.015	267.11304	1.371×10^{6}	1.398×10^{6}	1.357×10^{8}	3.042×10^{6}	8.113×10^{7}	2.476×10^{6}	7.960×10^{6}	Positive
393	*trans*-10-Heptadecenoic Acid	$C_{17}H_{32}O_2$	314.24594	7.546	313.23867	3.555×10^{7}	3.317×10^{7}	1.944×10^{7}	2.231×10^{7}	6.644×10^{7}	3.413×10^{7}	2.167×10^{7}	Negative
394	Tyrosol	$C_8H_{10}O_2$	138.06822	5.607	137.06092	2.602×10^{7}	3.210×10^{7}	3.734×10^{7}	4.127×10^{7}	3.726×10^{7}	2.778×10^{7}	3.089×10^{7}	Negative
395	7α-Hydroxytestosterone	$C_{19}H_{28}O_3$	304.20154	6.703	305.20881	4.754×10^{7}	2.985×10^{7}	4.367×10^{7}	2.966×10^{7}	1.724×10^{7}	3.420×10^{7}	2.928×10^{7}	Positive
396	5-Hydroxyindole-3-acetic acid	$C_{10}H_9NO_3$	191.05857	4.866	192.06583	2.498×10^{7}	2.359×10^{7}	2.272×10^{7}	4.770×10^{7}	4.704×10^{7}	3.846×10^{7}	2.641×10^{7}	Positive
397	5-Hydroxyindole-2-carboxylic acid	$C_9H_7NO_3$	177.04289	4.962	176.03561	1.782×10^{7}	1.702×10^{7}	6.240×10^{7}	2.344×10^{7}	3.671×10^{7}	2.590×10^{7}	4.512×10^{7}	Negative
398	Corticosterone	$C_{21}H_{30}O_4$	346.21238	9.593	345.20511	4.660×10^{7}	5.754×10^{7}	3.144×10^{7}	1.515×10^{7}	4.450×10^{7}	1.604×10^{7}	1.658×10^{7}	Negative
399	5-Phenylvaleric Acid	$C_{11}H_{14}O_2$	178.09963	5.383	177.09235	2.541×10^{7}	2.946×10^{7}	3.685×10^{7}	2.491×10^{7}	2.405×10^{7}	3.864×10^{7}	4.658×10^{7}	Negative
400	Benzyl 6-O-beta-D-glucopyranosyl-beta-D-glucopyranoside	$C_{19}H_{28}O_{11}$	396.18731	5.36	431.15668	3.276×10^{7}	3.577×10^{7}	4.802×10^{7}	5.660×10^{7}	1.923×10^{7}	1.627×10^{7}	1.645×10^{7}	Negative
401	Pyridoxine	$C_8H_{11}NO_3$	169.0742	5.039	214.0724	2.314×10^{7}	2.010×10^{7}	4.050×10^{7}	2.928×10^{7}	4.059×10^{7}	2.787×10^{7}	4.204×10^{7}	Negative

402	N'-(cyclohexylcarbonyl)-4-methyl-1,2,3-thiadiazole-5-carbohydrazide	$C_{11}H_{16}N_4O_2S$	268.09816	5.083	267.09086	2.397×10^7	3.500×10^7	1.156×10^8	2.449×10^7	1.867×10^7	2.071×10^6	1.793×10^6	Negative
403	Acipimox	$C_6H_6N_2O_3$	154.03801	5.43	155.04528	1.543×10^6	1.331×10^6	2.279×10^7	1.695×10^6	2.326×10^7	2.160×10^7	1.469×10^8	Positive
404	Jasmone	$C_{11}H_{16}O$	164.12036	5.294	165.12764	2.968×10^7	3.343×10^7	2.843×10^7	4.295×10^7	2.478×10^7	2.855×10^7	3.115×10^7	Positive
405	4-[2-(2-methylphenyl)-2-oxoethyl]benzamide	$C_{16}H_{15}NO_2$	253.11038	6.429	254.11766	3.039×10^6	2.869×10^6	9.135×10^7	7.371×10^6	6.211×10^7	1.220×10^7	3.742×10^7	Positive
406	Thr-Leu	$C_{10}H_{20}N_2O_4$	232.14258	1.133	216.12337	3.005×10^7	3.380×10^7	2.341×10^7	4.377×10^7	2.858×10^7	2.926×10^7	2.691×10^7	Positive
407	Sebacic acid	$C_{10}H_{18}O_4$	202.12073	6.073	203.12791	4.100×10^7	4.411×10^7	1.779×10^7	3.795×10^7	2.150×10^7	3.250×10^7	2.050×10^7	Positive
408	(+/-)12(13)-DiHOME	$C_{18}H_{34}O_4$	296.23525	7.985	295.22797	2.688×10^7	2.514×10^7	4.969×10^7	2.733×10^7	4.494×10^7	1.453×10^7	2.589×10^7	Negative
409	2-{4-[(4-methylphenyl)sulfonyl]piperazino}-1-morpholino-1-ethanone	$C_{17}H_{25}N_3O_4S$	367.16072	5.847	368.168	4.723×10^6	5.509×10^6	7.296×10^7	6.443×10^6	9.367×10^7	8.368×10^6	2.258×10^7	Positive
410	Nor-9-carboxy-δ9-THC	$C_{21}H_{28}O_4$	344.19647	6.055	345.20381	2.372×10^7	2.350×10^7	4.492×10^7	2.566×10^7	5.918×10^7	1.727×10^7	1.919×10^7	Positive
411	3-hydroxy-3,4-bis[(4-hydroxy-3-methoxyphenyl)methyl]oxolan-2-one	$C_{20}H_{22}O_7$	412.08324	8.682	413.09052	3.994×10^7	3.135×10^7	3.853×10^7	3.632×10^7	9.803×10^6	3.108×10^7	2.603×10^7	Positive
412	N-Acetyl-Dl-glutamic acid	$C_7H_{11}NO_5$	189.06398	1.538	172.06054	3.994×10^7	3.599×10^7	2.843×10^7	4.235×10^7	1.843×10^7	1.899×10^7	2.798×10^7	Positive
413	*L*-Adrenaline	$C_9H_{13}NO_3$	183.08975	5.609	184.09699	1.080×10^7	1.013×10^7	5.898×10^7	1.587×10^7	3.052×10^7	3.194×10^7	5.137×10^7	Positive
414	(2E)-3-(3,4-dimethoxyphenyl)prop-2-enoic acid	$C_{11}H_{12}O_4$	190.06321	5.339	191.07045	1.915×10^7	2.113×10^7	3.627×10^7	2.180×10^7	7.817×10^7	1.369×10^7	1.917×10^7	Positive
415	2-(1-{2-[(3-furylmethyl)amino]-2-oxoethyl}cyclohexyl)acetic acid	$C_{15}H_{21}NO_4$	279.14725	5.441	278.13997	1.025×10^7	1.341×10^7	3.012×10^7	2.017×10^7	1.504×10^7	8.297×10^7	3.736×10^7	Negative
416	Biotin	$C_{10}H_{16}N_2O_3S$	244.08407	5.899	245.09145	2.175×10^7	2.034×10^7	4.987×10^7	1.762×10^7	5.161×10^7	2.195×10^7	2.605×10^7	Positive
417	4-oxo-4-(5,6,7,8-tetrahydronaphthalen-1-ylamino)but-2-enoic acid	$C_{14}H_{15}NO_3$	245.10535	5.344	246.11263	1.977×10^7	1.714×10^7	5.591×10^7	1.891×10^7	3.492×10^7	2.401×10^7	3.850×10^7	Positive
418	Tetranor-12(S)-HETE	$C_{16}H_{26}O_3$	288.17095	6.407	289.17822	4.044×10^7	2.879×10^7	3.094×10^7	3.606×10^7	1.498×10^7	3.233×10^7	2.534×10^7	Positive
419	4-Toluenesulfonic acid	$C_7H_8O_3S$	172.01961	5.289	171.01233	3.361×10^7	3.576×10^7	3.579×10^7	2.648×10^7	2.537×10^7	2.627×10^7	2.392×10^7	Negative
420	2,6-Di-tert-butyl-1,4-benzoquinone	$C_{14}H_{20}O_2$	220.14652	5.706	221.15379	1.692×10^7	1.719×10^7	3.250×10^7	3.233×10^7	4.879×10^7	3.560×10^7	2.111×10^7	Positive
421	3-(phenethylethanimidoyl)tetrahydrofuran-2-one	$C_{14}H_{17}NO_2$	209.1417	5.601	192.1384	3.862×10^7	3.614×10^7	1.754×10^7	3.653×10^7	2.836×10^7	2.316×10^7	2.285×10^7	Positive
422	*D*-Xylonic Acid	$C_5H_{10}O_6$	166.04819	5.96	149.04477	4.112×10^7	3.190×10^7	2.764×10^7	3.383×10^7	2.657×10^7	1.937×10^7	2.168×10^7	Positive
423	Asp-Phe methyl ester	$C_{14}H_{18}N_2O_5$	294.12257	5.475	293.11482	1.403×10^7	1.698×10^7	7.331×10^7	1.870×10^7	2.412×10^7	1.329×10^7	4.149×10^7	Negative
424	*DL*-m-Tyrosine	$C_9H_{11}NO_3$	181.07424	5.389	182.08152	1.979×10^7	1.908×10^7	4.194×10^7	2.567×10^7	4.252×10^7	2.218×10^7	3.053×10^7	Positive
425	Ethyl paraben	$C_9H_{10}O_3$	166.06345	5.006	167.07079	2.135×10^7	2.614×10^7	2.764×10^7	3.842×10^7	2.409×10^7	2.930×10^7	3.328×10^7	Positive
426	6-Hydroxymelatonin	$C_{13}H_{16}N_2O_3$	248.11646	5.449	293.11465	1.845×10^7	1.738×10^7	7.034×10^7	1.814×10^7	2.214×10^7	1.329×10^7	4.000×10^7	Negative
427	2,3,4-Trihydroxybenzoic acid	$C_7H_6O_5$	170.02174	4.867	169.01446	3.033×10^7	3.680×10^7	2.684×10^7	7.454×10^7	1.400×10^7	8.434×10^6	8.492×10^6	Negative
428	6-methyl-5-nitroquinoline	$C_{10}H_8N_2O_2$	188.05875	5.491	187.05147	1.358×10^7	1.452×10^7	2.946×10^7	2.442×10^7	2.613×10^7	5.188×10^7	3.903×10^7	Negative

429	N-Acetyl-DL-phenylalanine	$C_{11}H_{13}NO_3$	207.08987	5.767	208.0972	4.771×10^{7}	4.292×10^{7}	2.623×10^{7}	1.043×10^{7}	4.050×10^{7}	1.140×10^{7}	1.800×10^{7}	Positive
430	N-[2-chloro-6-(trifluoromethoxy)phenyl]-2,2-dimethylpropanamide	$C_{12}H_{13}ClF_3NO_2$	295.05866	1.259	296.06593	3.212×10^{7}	2.859×10^{7}	2.435×10^{7}	2.332×10^{7}	5.996×10^{7}	1.358×10^{7}	1.502×10^{7}	Positive
431	Asaraldehyde	$C_{10}H_{12}O_4$	196.07384	4.974	195.06656	2.745×10^{7}	2.711×10^{7}	2.667×10^{7}	2.994×10^{7}	4.518×10^{7}	1.739×10^{7}	2.290×10^{7}	Negative
432	SQH	$C_{14}H_{22}N_6O_6$	392.14492	6.288	393.1522	2.139×10^{7}	2.125×10^{7}	1.981×10^{7}	3.529×10^{7}	3.249×10^{7}	4.485×10^{7}	1.758×10^{7}	Positive
433	Mycophenolic acid	$C_{17}H_{20}O_6$	137.06036	5.578	136.05305	2.189×10^{7}	2.327×10^{7}	2.284×10^{7}	2.784×10^{7}	4.552×10^{7}	2.540×10^{7}	2.357×10^{7}	Negative
434	1,3-Diphenylacetone	$C_{15}H_{14}O$	227.1312	5.85	228.13848	1.890×10^{7}	2.017×10^{7}	8.248×10^{7}	5.877×10^{7}	1.385×10^{6}	4.194×10^{6}	4.157×10^{6}	Positive
435	2'-Deoxyadenosine	$C_{10}H_{13}N_5O_3$	251.10114	3.905	252.10841	3.755×10^{7}	3.155×10^{7}	1.818×10^{7}	4.133×10^{7}	2.214×10^{7}	2.007×10^{7}	1.838×10^{7}	Positive
436	1-(3-ethyl-2,4-dihydroxy-6-methoxyphenyl)butan-1-one	$C_{13}H_{18}O_4$	238.12081	5.457	239.12795	2.405×10^{7}	2.462×10^{7}	2.676×10^{7}	3.744×10^{7}	3.363×10^{7}	1.951×10^{7}	2.191×10^{7}	Positive
437	Atropine	$C_{17}H_{23}NO_3$	289.16776	5.809	290.1751	8.795×10^{6}	8.333×10^{6}	4.091×10^{7}	1.701×10^{7}	5.849×10^{7}	2.517×10^{7}	2.622×10^{7}	Positive
438	Trolox	$C_{14}H_{18}O_4$	250.11818	6.403	249.1109	3.805×10^{7}	3.760×10^{7}	1.263×10^{7}	2.730×10^{7}	1.578×10^{7}	3.566×10^{7}	1.706×10^{7}	Negative
439	Picrotin	$C_{15}H_{18}O_7$	310.10568	5.335	309.09838	8.890×10^{6}	8.301×10^{6}	3.593×10^{7}	8.743×10^{6}	1.136×10^{8}	2.935×10^{6}	5.269×10^{6}	Negative
440	N-(1,3-benzodioxol-5-ylmethyl)-6-morpholinonicotinamide	$C_{18}H_{19}N_3O_4$	341.13249	5.601	342.13977	4.019×10^{7}	3.594×10^{7}	9.049×10^{6}	2.847×10^{7}	1.908×10^{7}	2.480×10^{7}	2.560×10^{7}	Positive
441	N'-(4-methoxy-1,3-benzothiazol-2-yl)-N,N-dimethyliminoformamide	$C_{11}H_{13}N_3OS$	235.08205	5.57	236.08941	7.898×10^{6}	7.193×10^{6}	3.865×10^{7}	2.207×10^{7}	5.287×10^{7}	3.513×10^{7}	1.910×10^{7}	Positive
442	D(-)-Amygdalin	$C_{20}H_{27}NO_{11}$	457.15902	5.033	456.15187	1.284×10^{7}	1.165×10^{7}	5.052×10^{7}	1.259×10^{7}	5.139×10^{7}	1.623×10^{7}	2.748×10^{7}	Negative
443	ethyl 5-cyano-6-piperazino-2-(trifluoromethyl)nicotinate	$C_{14}H_{15}F_3N_4O_2$	328.11344	4.827	329.1207	2.946×10^{7}	3.073×10^{7}	3.312×10^{7}	2.918×10^{7}	1.956×10^{7}	1.782×10^{7}	2.246×10^{7}	Positive
444	Coenzyme Q2	$C_{19}H_{26}O_4$	140.1124	6.26	319.18799	3.436×10^{7}	2.901×10^{7}	2.190×10^{7}	4.241×10^{7}	6.465×10^{6}	2.995×10^{7}	1.678×10^{7}	Positive
445	4-morpholinobenzoic acid	$C_{11}H_{13}NO_3$	207.08972	5.577	208.09716	1.916×10^{7}	1.973×10^{7}	2.546×10^{7}	2.887×10^{7}	1.553×10^{7}	3.703×10^{7}	3.414×10^{7}	Positive
446	(11E,15Z)-9,10,13-trihydroxyoctadeca-11,15-dienoic acid	$C_{18}H_{32}O_5$	350.20715	6.277	351.21438	1.715×10^{7}	1.631×10^{7}	3.979×10^{7}	1.691×10^{7}	6.459×10^{7}	1.282×10^{7}	1.166×10^{7}	Positive
447	N-(2,5-dichlorobenzyl)-5-ethyl-1H-indole-2-carboxamide	$C_{18}H_{16}Cl_2N_2O$	346.06953	6.077	345.06226	2.102×10^{7}	2.149×10^{7}	7.545×10^{7}	5.305×10^{7}	2.350×10^{6}	1.749×10^{6}	3.136×10^{6}	Negative
448	Fenvalerate	$C_{25}H_{22}ClNO_3$	451.14828	5.265	452.15556	1.990×10^{7}	1.873×10^{7}	1.962×10^{7}	2.720×10^{7}	3.613×10^{7}	3.267×10^{7}	2.245×10^{7}	Positive
449	Phe-Phe	$C_{18}H_{20}N_2O_3$	312.14886	5.591	311.14158	1.081×10^{7}	1.008×10^{7}	4.190×10^{7}	2.025×10^{7}	2.847×10^{7}	3.153×10^{7}	3.182×10^{7}	Negative
450	4-acetyl-4-(ethoxycarbonyl)heptanedioic acid	$C_{12}H_{18}O_7$	274.10296	4.911	275.11024	5.044×10^{7}	5.935×10^{7}	4.437×10^{6}	1.744×10^{7}	1.226×10^{7}	9.812×10^{6}	2.016×10^{7}	Positive
451	α-Cyclodextrin	$C_{36}H_{60}O_{30}$	972.31714	1.451	973.32441	3.672×10^{7}	3.405×10^{7}	1.145×10^{7}	3.189×10^{7}	1.333×10^{7}	7.514×10^{6}	3.861×10^{7}	Positive
452	Picrotoxinin	$C_{15}H_{16}O_6$	309.12121	5.544	310.12858	5.000×10^{6}	4.057×10^{6}	5.115×10^{7}	5.569×10^{6}	7.959×10^{7}	7.275×10^{6}	2.070×10^{7}	Positive
453	Tretinoin	$C_{20}H_{28}O_2$	300.2091	9.128	299.20182	1.103×10^{7}	1.276×10^{7}	2.490×10^{7}	3.548×10^{6}	8.147×10^{7}	5.388×10^{6}	3.299×10^{7}	Negative
454	*DL*-α-Methoxyphenylacetic acid	$C_9H_{10}O_3$	120.05762	5.327	119.0503	2.921×10^{7}	2.758×10^{7}	3.164×10^{7}	3.030×10^{7}	1.625×10^{7}	1.752×10^{7}	1.931×10^{7}	Negative
455	*L*-Saccharopine	$C_{11}H_{20}N_2O_6$	276.13219	1.275	277.13948	4.640×10^{7}	4.546×10^{7}	2.135×10^{6}	7.135×10^{7}	3.089×10^{6}	2.641×10^{6}	7.169×10^{5}	Positive
456	Lignoceric Acid	$C_{24}H_{48}O_2$	368.36583	11.161	367.35855	1.541×10^{7}	1.754×10^{7}	2.539×10^{7}	2.199×10^{7}	3.839×10^{7}	4.126×10^{7}	1.176×10^{7}	Negative
457	4-Methoxycinnamic Acid	$C_{10}H_{10}O_3$	178.0631	5.586	177.05604	1.043×10^{7}	2.076×10^{7}	4.066×10^{7}	2.778×10^{7}	3.350×10^{7}	1.823×10^{7}	2.030×10^{7}	Negative
458	Tyramine	$C_8H_{11}NO$	137.08427	5.591	182.08251	1.106×10^{7}	1.211×10^{7}	3.815×10^{7}	1.801×10^{7}	3.096×10^{7}	3.126×10^{7}	2.992×10^{7}	Negative

459	LPC 18:3	$C_{26}H_{48}NO_7P$	563.32373	8.371	562.31645	6.194×10^{7}	6.249×10^{7}	1.600×10^{7}	1.190×10^{7}	7.917×10^{6}	3.661×10^{6}	6.391×10^{6}	Negative
460	(R)-3-Hydroxy myristic acid	$C_{14}H_{28}O_3$	244.20386	7.172	243.19659	3.424×10^{7}	3.382×10^{7}	1.244×10^{7}	2.801×10^{7}	1.522×10^{7}	3.230×10^{7}	1.212×10^{7}	Negative
461	5-[(1-benzothiophen-3-ylmethyl)sulfanyl]-1-methyl-4-nitro-1H-imidazole	$C_{13}H_{11}N_3O_2S_2$	193.07429	5.31	194.08196	1.642×10^{7}	1.473×10^{7}	3.545×10^{7}	2.309×10^{7}	2.621×10^{7}	1.846×10^{7}	3.223×10^{7}	Positive
462	2-(3-nitrophenyl)-1,3-diphenylimidazolidine	$C_{21}H_{19}N_3O_2$	345.14237	5.008	346.14964	3.648×10^{7}	2.428×10^{7}	2.073×10^{7}	1.444×10^{7}	3.852×10^{7}	1.091×10^{7}	2.085×10^{7}	Positive
463	Nicotinate ribonucleoside	$C_{11}H_{13}NO_6$	255.07427	1.492	256.08156	3.070×10^{7}	2.894×10^{7}	1.232×10^{7}	4.425×10^{7}	1.099×10^{7}	1.108×10^{7}	2.604×10^{7}	Positive
464	6-(7-methyloctyl)-1H,3H,4H,6H-furo[3,4-c]furan-1-one	$C_{15}H_{24}O_3$	252.17068	6.213	253.17796	8.158×10^{6}	4.447×10^{6}	2.801×10^{7}	2.314×10^{7}	5.082×10^{7}	3.069×10^{7}	1.515×10^{7}	Positive
465	JNJ-1661010	$C_{19}H_{19}N_5OS$	365.13241	5.227	364.12516	8.857×10^{6}	8.812×10^{6}	6.017×10^{7}	1.655×10^{7}	3.407×10^{7}	1.406×10^{7}	1.687×10^{7}	Negative
466	N'2-(2-hydroxybenzylidene)-5-nitrofuran-2-carbohydrazide	$C_{12}H_9N_3O_5$	297.03795	7.809	298.04523	2.867×10^{7}	2.249×10^{7}	2.782×10^{7}	2.724×10^{7}	1.075×10^{7}	2.202×10^{7}	2.002×10^{7}	Positive
467	Cytosine	$C_4H_5N_3O$	111.04359	1.464	112.05087	2.978×10^{7}	2.810×10^{7}	1.456×10^{7}	4.334×10^{7}	1.677×10^{7}	1.250×10^{7}	1.086×10^{7}	Positive
468	3-amino-2-phenyl-2H-pyrazolo[4,3-c]pyridine-4,6-diol	$C_{12}H_{10}N_4O_2$	110.04921	5.864	243.08788	2.800×10^{7}	2.373×10^{7}	8.941×10^{6}	2.000×10^{7}	5.490×10^{7}	1.064×10^{7}	8.785×10^{6}	Positive
469	2,3-Dinor-11β-prostaglandin F2α	$C_{18}H_{30}O_5$	362.1946	5.953	361.18732	1.771×10^{7}	1.763×10^{7}	2.962×10^{7}	2.914×10^{7}	3.759×10^{7}	1.610×10^{7}	6.960×10^{6}	Negative
470	*L*-Tyrosine	$C_9H_{11}NO_3$	181.07418	5.99	182.08147	1.865×10^{6}	1.982×10^{6}	2.562×10^{7}	2.192×10^{6}	1.133×10^{8}	2.851×10^{6}	6.445×10^{6}	Positive
471	*P*-Coumaroyl Agmatine	$C_{14}H_{20}N_4O_2$	276.15771	5.551	275.15022	1.520×10^{7}	2.845×10^{7}	2.085×10^{7}	2.719×10^{7}	2.105×10^{7}	1.878×10^{7}	2.266×10^{7}	Negative
472	Acetaminophen glucuronide	$C_{14}H_{17}NO_8$	327.09575	5.073	326.08847	8.579×10^{6}	9.289×10^{6}	4.996×10^{7}	1.150×10^{7}	3.228×10^{7}	1.446×10^{7}	2.728×10^{7}	Negative
473	2-[(3S)-1-(3-Methoxybenzyl)-3-pyrrolidinyl]-1,3-benzothiazole	$C_{19}H_{20}N_2OS$	324.13216	5.493	325.1394	1.658×10^{7}	8.710×10^{6}	3.234×10^{7}	1.188×10^{7}	3.770×10^{7}	1.852×10^{7}	2.706×10^{7}	Positive
474	Prostaglandin A3	$C_{20}H_{28}O_4$	368.17048	5.874	367.16322	3.425×10^{7}	3.078×10^{7}	6.298×10^{6}	2.492×10^{7}	4.512×10^{6}	1.845×10^{7}	3.121×10^{7}	Negative
475	4-Hydroxybenzoic acid	$C_7H_6O_3$	138.03186	1.149	139.03914	1.767×10^{7}	1.864×10^{7}	2.325×10^{7}	2.220×10^{7}	2.441×10^{7}	2.199×10^{7}	2.163×10^{7}	Positive
476	4-Methylvaleric Acid	$C_6H_{12}O_2$	116.08389	5.563	115.07657	2.013×10^{7}	2.261×10^{7}	2.324×10^{7}	2.437×10^{7}	3.086×10^{7}	1.331×10^{7}	1.409×10^{7}	Negative
477	2-{2-oxo-2-[(2-oxo-3-azepanyl)amino]ethoxy}acetic acid	$C_{10}H_{16}N_2O_5$	244.10764	6.412	243.10032	2.085×10^{7}	2.374×10^{7}	2.361×10^{7}	1.708×10^{7}	1.995×10^{7}	1.879×10^{7}	2.368×10^{7}	Negative
478	4-[1-(acetyloxy)prop-2-en-1-yl]-2-methoxyphenyl 2-methylpropanoate	$C_{16}H_{20}O_5$	309.15768	5.272	310.16494	7.842×10^{6}	4.539×10^{6}	4.792×10^{7}	1.040×10^{7}	3.891×10^{7}	8.232×10^{6}	2.771×10^{7}	Positive
479	2-Naphthol	$C_{10}H_8O$	144.05748	5.931	145.06459	1.788×10^{7}	1.663×10^{7}	2.947×10^{7}	2.330×10^{7}	2.943×10^{7}	1.525×10^{7}	1.315×10^{7}	Positive
480	(R)-Prunasin	$C_{14}H_{17}NO_6$	295.10561	5.114	296.1129	1.559×10^{7}	1.575×10^{7}	3.212×10^{7}	1.816×10^{7}	2.645×10^{7}	1.173×10^{7}	2.371×10^{7}	Positive
481	3-[3-(beta-D-Glucopyranosyloxy)-2-methoxyphenyl]propanoic acid	$C_{16}H_{22}O_9$	380.10853	5.527	381.11581	1.920×10^{7}	1.361×10^{7}	1.425×10^{7}	2.503×10^{7}	2.152×10^{7}	2.494×10^{7}	2.360×10^{7}	Positive
482	4-Aminohippuric acid	$C_9H_{10}N_2O_3$	194.06919	5.301	193.06204	6.263×10^{6}	5.547×10^{6}	3.039×10^{7}	1.340×10^{7}	1.632×10^{7}	3.008×10^{7}	4.009×10^{7}	Negative
483	6 β-Hydroxycortisol	$C_{21}H_{30}O_6$	360.18972	5.589	361.19696	4.926×10^{6}	3.979×10^{6}	1.517×10^{7}	9.064×10^{6}	2.487×10^{6}	2.331×10^{7}	8.125×10^{7}	Positive
484	(±)12(13)-DiHOME	$C_{18}H_{34}O_4$	314.24597	7.303	313.2387	1.880×10^{7}	1.693×10^{7}	2.911×10^{7}	1.578×10^{7}	3.304×10^{7}	1.095×10^{7}	1.398×10^{7}	Negative
485	2-Amino-3-(4-hydroxy-3-methoxyphenyl)propanoic acid	$C_{10}H_{13}NO_4$	165.0792	5.371	164.0719	1.846×10^{7}	1.546×10^{7}	2.970×10^{7}	1.137×10^{7}	2.318×10^{7}	1.457×10^{7}	2.503×10^{7}	Negative
486	1-Caffeoylquinic Acid	$C_{16}H_{18}O_9$	354.09523	5.088	353.08796	1.451×10^{7}	8.966×10^{6}	2.847×10^{7}	2.092×10^{7}	2.275×10^{7}	2.268×10^{7}	1.908×10^{7}	Negative

487	*D*-Proline	$C_5H_9NO_2$	115.06332	5.068	114.05606	1.741×10^7	1.879×10^7	2.360×10^7	1.971×10^7	2.614×10^7	1.449×10^7	1.528×10^7	Negative
488	N-Acetyl-D-lactosamine	$C_{14}H_{25}NO_{11}$	383.14254	1.442	384.14974	2.354×10^7	2.481×10^7	1.196×10^7	2.186×10^7	1.326×10^7	2.469×10^7	1.504×10^7	Positive
489	N-Methyldioctylamine	$C_{17}H_{37}N$	255.29272	7.161	256.29999	1.232×10^7	1.068×10^7	1.513×10^7	2.938×10^7	6.366×10^6	5.134×10^7	9.775×10^6	Positive
490	Aldosterone	$C_{21}H_{28}O_5$	360.19014	5.593	359.18287	4.272×10^6	3.658×10^6	1.332×10^7	6.886×10^6	1.638×10^6	2.313×10^7	8.132×10^7	Negative
491	5-[(Benzoyloxy)methyl]-4,5,6-trihydroxy-2-cyclohexen-1-yl benzoate	$C_{21}H_{20}O_7$	430.13264	3.643	429.12537	2.605×10^7	2.225×10^7	1.541×10^7	1.769×10^7	2.502×10^7	1.480×10^7	9.615×10^6	Negative
492	Citrinin	$C_{13}H_{14}O_5$	232.0737	5.367	233.081	1.726×10^6	1.889×10^6	2.807×10^7	2.268×10^6	9.177×10^7	1.104×10^6	2.808×10^6	Positive
493	*P*-Hydroxybenzaldehyde	$C_7H_6O_2$	122.03344	1.124	123.04074	1.734×10^7	1.734×10^7	1.653×10^7	2.277×10^7	1.630×10^7	1.971×10^7	1.826×10^7	Positive
494	5-([1,1'-biphenyl]-2-ylamino)-5-oxopentanoic acid	$C_{17}H_{17}NO_3$	283.12119	5.73	284.12821	1.591×10^7	2.096×10^7	2.583×10^7	1.119×10^7	3.073×10^7	1.061×10^7	1.183×10^7	Positive
495	N-[3-(1H-imidazol-1-yl)propyl]-5-methoxy-1H-indole-2-carboxamide	$C_{16}H_{18}N_4O_2$	298.13927	5.35	299.14654	1.991×10^7	2.242×10^7	1.201×10^7	1.849×10^7	2.029×10^7	1.755×10^7	1.587×10^7	Positive
496	Ip7G	$C_{16}H_{23}N_5O_5$	365.16863	5.414	348.16531	4.216×10^6	6.244×10^6	5.215×10^7	8.963×10^6	3.124×10^7	6.721×10^6	1.651×10^7	Positive
497	4-hydroxy-3-(3-methylbut-2-en-1-yl)benzoic acid	$C_{12}H_{14}O_3$	206.09465	5.512	207.10202	3.993×10^6	3.944×10^6	2.774×10^7	5.697×10^6	8.004×10^7	2.009×10^6	2.112×10^6	Positive
498	Morphine	$C_{17}H_{19}NO_3$	285.13652	6.09	286.1438	4.939×10^6	5.070×10^6	3.413×10^7	9.244×10^6	4.105×10^7	1.576×10^7	1.425×10^7	Positive
499	*D*-Glucosamine	$C_6H_{13}NO_5$	179.07974	1.285	214.04915	1.528×10^7	1.865×10^7	1.648×10^7	1.121×10^7	1.433×10^7	1.893×10^7	2.881×10^7	Negative
500	Glycitein	$C_{16}H_{12}O_5$	284.06854	6.852	283.06127	1.539×10^6	1.068×10^6	6.000×10^7	3.257×10^6	5.368×10^7	8.556×10^5	2.424×10^6	Negative
501	Butylparaben	$C_{11}H_{14}O_3$	148.08896	5.658	147.0817	1.518×10^7	1.471×10^7	1.521×10^7	1.925×10^7	2.855×10^7	1.720×10^7	1.204×10^7	Negative
502	3-[(4-chlorophenyl)thio]-1-phenylprop-2-en-1-one	$C_{15}H_{11}ClOS$	296.00427	11.474	297.01155	1.417×10^7	1.618×10^7	1.404×10^7	1.181×10^7	1.983×10^7	1.765×10^7	2.805×10^7	Positive
503	tert-Butyl N-[1-(aminocarbonyl)-3-methylbutyl]carbamate	$C_{11}H_{22}N_2O_3$	230.16311	5.32	231.17039	2.088×10^7	2.728×10^7	9.640×10^6	4.239×10^7	5.266×10^6	9.005×10^6	6.761×10^6	Positive
504	11-Deoxy prostaglandin F1*α*	$C_{20}H_{36}O_4$	362.24344	6.306	363.25071	1.067×10^7	1.295×10^7	1.488×10^7	1.724×10^7	4.311×10^7	8.525×10^6	1.342×10^7	Positive
505	7-Hydroxy-3,4-dihydrocarbostyril	$C_9H_9NO_2$	163.06346	5.107	208.06169	2.137×10^7	1.918×10^7	1.666×10^7	1.498×10^7	9.557×10^6	1.350×10^7	2.265×10^7	Negative
506	LPI 18:3	$C_{27}H_{47}O_{12}P$	594.28165	8.729	593.27437	4.600×10^7	6.013×10^7	3.377×10^6	4.197×10^6	2.338×10^6	6.300×10^5	1.145×10^6	Negative
507	Thymine	$C_5H_6N_2O_2$	126.04276	4.925	127.05003	2.001×10^7	1.946×10^7	1.386×10^7	2.291×10^7	2.451×10^7	7.906×10^6	7.802×10^6	Positive
508	*Dl*-3-Hydroxynorvaline	$C_5H_{11}NO_3$	133.07415	6.647	134.08143	7.656×10^6	9.001×10^6	1.043×10^7	2.254×10^7	8.300×10^6	3.495×10^7	2.353×10^7	Positive
509	Kaempferol	$C_{15}H_{10}O_6$	286.04777	5.774	287.05504	7.797×10^6	9.314×10^6	1.303×10^7	1.679×10^7	9.587×10^6	3.452×10^7	2.510×10^7	Positive
510	1-(4-butylphenyl)-3-(dimethylamino)propan-1-one hydrochloride	$C_{15}H_{23}NO$	211.19377	7.769	212.20105	1.896×10^7	1.850×10^7	1.522×10^7	1.568×10^7	1.579×10^7	1.552×10^7	1.568×10^7	Positive
511	Palmitoleic Acid	$C_{16}H_{30}O_2$	276.20891	8.009	277.21619	2.727×10^7	3.036×10^7	9.693×10^6	1.306×10^7	2.245×10^7	6.499×10^6	5.842×10^6	Positive
512	*L*-Dopa	$C_9H_{11}NO_4$	197.06914	5.317	196.06186	1.138×10^7	1.189×10^7	2.304×10^7	1.723×10^7	1.989×10^7	1.788×10^7	1.379×10^7	Negative
513	Obscurolide A1	$C_{15}H_{17}NO_5$	291.11081	5.046	292.11809	2.861×10^7	1.872×10^7	8.586×10^6	8.719×10^6	1.564×10^7	5.982×10^6	2.881×10^7	Positive
514	Diacetoxyscirpenol	$C_{19}H_{26}O_7$	348.15753	5.984	349.16463	1.047×10^7	8.558×10^6	1.664×10^7	1.431×10^7	4.726×10^7	1.081×10^7	6.576×10^6	Positive
515	Linoleoyl Ethanolamide	$C_{20}H_{37}NO_2$	323.28014	7.168	324.28741	1.595×10^7	1.576×10^7	1.836×10^7	1.807×10^7	1.503×10^7	1.806×10^7	1.336×10^7	Positive

516	2-benzyl-6-hydroxy-2-azabicyclo[2.2.2]octan-3-one	$C_{14}H_{17}NO_2$	231.12352	5.607	232.13079	4.472×10^{7}	3.461×10^{7}	1.633×10^{6}	2.687×10^{7}	1.889×10^{6}	1.969×10^{6}	1.648×10^{6}	Positive
517	2,6-Dihydroxypurine	$C_5H_4N_4O_2$	152.03355	2.256	151.02627	2.301×10^{7}	2.539×10^{7}	9.776×10^{6}	1.761×10^{7}	1.432×10^{7}	9.963×10^{6}	1.302×10^{7}	Negative
518	Quinoline-4-carboxylic acid	$C_{10}H_7NO_2$	173.04783	5.34	174.05516	1.359×10^{7}	1.360×10^{7}	1.378×10^{7}	1.634×10^{7}	2.156×10^{7}	2.135×10^{7}	1.247×10^{7}	Positive
519	Farnesyl pyrophosphate	$C_{15}H_{28}O_7P_2$	382.12703	5.517	381.11979	5.910×10^{6}	7.433×10^{6}	4.791×10^{6}	1.077×10^{7}	1.099×10^{7}	4.395×10^{6}	6.741×10^{7}	Negative
520	Muramic acid	$C_9H_{17}NO_7$	251.10048	1.278	252.10783	1.727×10^{7}	1.622×10^{7}	1.209×10^{7}	2.302×10^{7}	1.590×10^{7}	7.486×10^{6}	1.954×10^{7}	Positive
521	3-(benzylthio)-5-[4-(tert-butyl)phenyl]-4-methyl-4H-1,2,4-triazole	$C_{20}H_{23}N_3S$	337.16637	6.074	338.17365	1.323×10^{7}	1.436×10^{7}	1.338×10^{7}	1.850×10^{7}	2.677×10^{7}	1.686×10^{7}	8.071×10^{6}	Positive
522	Maltopentaose	$C_{30}H_{52}O_{26}$	850.25655	1.537	851.26383	2.761×10^{7}	2.433×10^{7}	8.574×10^{6}	2.554×10^{7}	7.468×10^{6}	8.878×10^{6}	8.706×10^{6}	Positive
523	4,5-Dicaffeoylquinic acid	$C_{25}H_{24}O_{12}$	516.12728	5.467	515.12001	4.261×10^{6}	3.686×10^{6}	3.383×10^{7}	8.359×10^{6}	1.676×10^{7}	1.849×10^{7}	2.410×10^{7}	Negative
524	13(S)-HOTrE	$C_{18}H_{30}O_3$	294.21941	6.412	295.22682	1.415×10^{7}	1.186×10^{7}	1.890×10^{7}	1.283×10^{7}	3.387×10^{7}	9.100×10^{6}	8.585×10^{6}	Positive
525	10-Nitrolinoleate	$C_{18}H_{31}NO_4$	285.23063	6.751	308.21988	1.392×10^{7}	1.710×10^{7}	2.139×10^{7}	1.472×10^{7}	1.044×10^{7}	8.447×10^{6}	2.187×10^{7}	Positive
526	(±)9(10)-DiHOME	$C_{18}H_{34}O_4$	314.24604	6.733	313.23877	1.311×10^{7}	1.333×10^{7}	2.024×10^{7}	1.161×10^{7}	2.887×10^{7}	9.976×10^{6}	1.050×10^{7}	Negative
527	Nonadecanoic acid	$C_{19}H_{38}O_2$	298.28729	10.754	299.29457	1.845×10^{7}	1.792×10^{7}	1.651×10^{7}	1.196×10^{7}	1.477×10^{7}	1.407×10^{7}	1.270×10^{7}	Positive
528	Lauric Acid	$C_{12}H_{24}O_2$	200.17796	6.64	223.16707	1.844×10^{7}	1.827×10^{7}	1.939×10^{7}	1.676×10^{7}	6.980×10^{6}	1.496×10^{7}	1.142×10^{7}	Positive
529	Prostaglandin F3α	$C_{20}H_{32}O_5$	398.2343	9.195	397.22702	1.644×10^{7}	1.277×10^{7}	2.534×10^{7}	1.384×10^{7}	1.055×10^{7}	1.506×10^{7}	1.164×10^{7}	Negative
530	Prostaglandin G2	$C_{20}H_{32}O_6$	368.21918	6.141	367.2119	3.029×10^{7}	2.235×10^{7}	5.935×10^{6}	2.831×10^{7}	6.614×10^{6}	7.643×10^{6}	4.367×10^{6}	Negative
531	Kinetin 9-riboside	$C_{15}H_{17}N_5O_5$	347.12208	5.063	346.11481	6.021×10^{6}	6.322×10^{6}	6.416×10^{7}	1.268×10^{7}	7.716×10^{6}	3.435×10^{6}	5.145×10^{6}	Negative
532	4-[2-(2-oxo-1-imidazolidinyl)ethyl]-1lambda~6~,4-thiazinane-1,1-dione	$C_9H_{17}N_3O_3S$	248.10435	5.572	293.10214	2.099×10^{7}	1.730×10^{7}	1.744×10^{7}	8.352×10^{6}	2.590×10^{7}	6.025×10^{6}	8.899×10^{6}	Negative
533	6-methyl-7-nitro-2,3-dihydro-1,4-benzodioxine	$C_9H_9NO_4$	177.04282	5.123	178.0501	8.586×10^{6}	6.208×10^{6}	1.745×10^{7}	1.232×10^{7}	1.994×10^{7}	1.807×10^{7}	2.176×10^{7}	Positive
534	Valylproline	$C_{10}H_{18}N_2O_3$	214.13196	4.911	215.13924	1.927×10^{7}	1.825×10^{7}	1.260×10^{7}	2.624×10^{7}	7.786×10^{6}	9.365×10^{6}	1.065×10^{7}	Positive
535	Prostaglandin H2	$C_{20}H_{32}O_5$	334.21249	7.123	379.21065	1.912×10^{7}	2.096×10^{7}	1.072×10^{7}	1.459×10^{7}	1.820×10^{7}	1.107×10^{7}	9.202×10^{6}	Negative
536	N-Acetyl-α-D-glucosamine 1-phosphate	$C_8H_{16}NO_9P$	301.05638	1.276	300.0491	1.330×10^{7}	1.389×10^{7}	1.200×10^{7}	1.271×10^{7}	7.854×10^{6}	1.926×10^{7}	2.458×10^{7}	Negative
537	Terbutaline	$C_{12}H_{19}NO_3$	225.13669	5.106	226.14393	7.840×10^{6}	5.561×10^{6}	2.662×10^{7}	1.275×10^{7}	1.882×10^{7}	1.446×10^{7}	1.729×10^{7}	Positive
538	Equol	$C_{15}H_{14}O_3$	242.09053	4.919	241.08325	1.268×10^{7}	1.296×10^{7}	1.512×10^{7}	1.721×10^{7}	2.031×10^{7}	9.258×10^{6}	1.560×10^{7}	Negative
539	N1-(3,5-dichlorophenyl)-3-amino-3-hydroxyiminopropanamide	$C_9H_9Cl_2N_3O_2$	282.98606	11.443	283.99332	5.157×10^{6}	5.480×10^{6}	4.883×10^{6}	5.133×10^{6}	5.149×10^{6}	1.829×10^{7}	5.819×10^{7}	Positive
540	Guggulsterone	$C_{21}H_{28}O_2$	312.20915	8.309	311.20187	1.493×10^{7}	1.551×10^{7}	1.644×10^{7}	1.420×10^{7}	1.467×10^{7}	1.264×10^{7}	1.320×10^{7}	Negative
541	8-iso-15-keto Prostaglandin E2	$C_{20}H_{30}O_5$	350.20795	6.268	395.20581	8.833×10^{6}	1.014×10^{7}	2.415×10^{7}	7.839×10^{6}	3.865×10^{7}	5.372×10^{6}	5.441×10^{6}	Negative
542	6-phenyl-3,4-dihydro-1H-2,5-benzoxazocin-1-one	$C_{16}H_{13}NO_2$	251.09474	6.323	252.102	1.612×10^{6}	1.314×10^{6}	4.000×10^{7}	4.472×10^{6}	2.719×10^{7}	8.421×10^{6}	1.675×10^{7}	Positive
543	2-(14,15-Epoxyeicosatrienoyl) glycerol	$C_{23}H_{38}O_5$	394.26969	8.177	395.27697	1.452×10^{7}	1.442×10^{7}	1.643×10^{7}	1.430×10^{7}	1.412×10^{7}	1.436×10^{7}	1.104×10^{7}	Positive
544	Glycerol-3-phosphate	$C_3H_9O_6P$	172.01412	1.215	173.0214	1.887×10^{7}	1.922×10^{7}	1.376×10^{7}	1.632×10^{7}	1.541×10^{7}	7.601×10^{6}	7.277×10^{6}	Positive

545	5-[(E)-2-(3,5-dihydroxyphenyl)ethenyl]-2-methoxybenzene-1,3-diol	$C_{15}H_{14}O_{5}$	274.0844	5.354	275.09164	1.065×10^{6}	1.032×10^{6}	2.098×10^{7}	9.718×10^{5}	7.206×10^{7}	1.229×10^{6}	1.044×10^{6}	Positive
546	Boc-beta-cyano-L-alanine	$C_{9}H_{14}N_{2}O_{4}$	214.09554	5.39	215.10281	6.438×10^{6}	6.329×10^{6}	1.382×10^{7}	1.617×10^{7}	1.377×10^{7}	2.193×10^{7}	1.874×10^{7}	Positive
547	Methyl 3-indolyacetate	$C_{11}H_{11}NO_{2}$	189.07937	5.61	190.08648	8.642×10^{6}	7.266×10^{6}	1.426×10^{7}	7.050×10^{6}	3.978×10^{7}	9.457×10^{6}	1.057×10^{7}	Positive
548	2,4-Dinitrophenol	$C_{6}H_{4}N_{2}O_{5}$	184.01209	5.956	183.00481	8.349×10^{6}	8.576×10^{6}	1.171×10^{7}	7.434×10^{6}	5.036×10^{7}	5.230×10^{6}	4.898×10^{6}	Negative
549	N,N-dimethyl-5-nitro-6-[3-(trifluoromethyl)phenoxy]pyrimidin-4-amine	$C_{13}H_{11}F_{3}N_{4}O_{3}$	328.07701	2.027	329.08429	9.991×10^{6}	9.688×10^{6}	9.139×10^{6}	8.929×10^{6}	8.550×10^{6}	7.986×10^{6}	4.079×10^{7}	Positive
550	Benzoylecgonine	$C_{16}H_{19}NO_{4}$	289.13543	5.785	290.14277	8.129×10^{6}	8.369×10^{6}	1.317×10^{7}	1.222×10^{7}	7.346×10^{6}	2.144×10^{7}	2.160×10^{7}	Positive
551	Monolaurin	$C_{15}H_{30}O_{4}$	296.19654	6.036	297.2038	1.522×10^{7}	1.669×10^{7}	2.203×10^{7}	2.446×10^{7}	4.536×10^{6}	4.616×10^{6}	3.437×10^{6}	Positive
552	Eicosapentaenoic acid	$C_{20}H_{30}O_{2}$	302.22477	9.901	301.21749	5.980×10^{6}	9.243×10^{6}	1.117×10^{7}	1.035×10^{6}	4.933×10^{7}	2.816×10^{6}	1.141×10^{7}	Negative
553	5,6-dimethyl-4-oxo-4H-pyran-2-carboxylic acid	$C_{8}H_{8}O_{4}$	168.04269	5.361	169.05011	1.248×10^{7}	1.283×10^{7}	1.362×10^{7}	1.006×10^{7}	1.854×10^{7}	1.127×10^{7}	1.217×10^{7}	Positive
554	Pentadecanoic acid	$C_{15}H_{30}O_{2}$	242.22463	9.927	241.21736	1.303×10^{7}	1.483×10^{7}	1.363×10^{7}	1.043×10^{7}	1.483×10^{7}	1.212×10^{7}	1.129×10^{7}	Negative
555	Prostaglandin F2α-1-glyceryl ester	$C_{23}H_{40}O_{7}$	450.25958	5.992	451.26708	7.585×10^{6}	8.244×10^{6}	1.191×10^{7}	1.325×10^{7}	9.526×10^{6}	1.832×10^{7}	2.087×10^{7}	Positive
556	Artemisinin	$C_{15}H_{22}O_{5}$	282.14656	5.516	283.1531	1.099×10^{7}	8.751×10^{6}	1.149×10^{7}	1.133×10^{7}	2.147×10^{7}	9.656×10^{6}	1.577×10^{7}	Positive
557	1-adamantyl(piperidino)methanone	$C_{16}H_{25}NO$	247.19131	8.125	248.19858	1.830×10^{7}	1.042×10^{7}	8.843×10^{6}	7.594×10^{6}	2.759×10^{7}	9.043×10^{6}	7.457×10^{6}	Positive
558	(2S)-2-(2-hydroxypropan-2-yl)-2H,3H,7H-furo[3,2-g]chromen-7-one	$C_{14}H_{14}O_{4}$	246.08937	5.347	247.09665	2.665×10^{6}	3.802×10^{6}	1.973×10^{7}	4.331×10^{6}	5.223×10^{7}	2.091×10^{6}	3.902×10^{6}	Positive
559	3-(3-furylmethylidene)-1,5-dioxaspiro[5.5]undecane-2,4-dione	$C_{14}H_{14}O_{5}$	262.08418	5.356	263.09157	4.033×10^{6}	3.519×10^{6}	2.487×10^{7}	4.201×10^{6}	4.412×10^{7}	2.211×10^{6}	5.616×10^{6}	Positive
560	4-Hydroxy-L-Glutamic Acid	$C_{5}H_{9}NO_{5}$	163.048	1.285	208.04618	3.366×10^{6}	3.130×10^{6}	1.204×10^{7}	4.308×10^{6}	1.132×10^{7}	2.876×10^{7}	2.560×10^{7}	Negative
561	N-2-Hydroxycyclopentyladenosine	$C_{15}H_{21}N_{5}O_{5}$	351.15396	5.361	352.16125	9.487×10^{6}	9.160×10^{6}	1.207×10^{7}	9.640×10^{6}	2.298×10^{7}	1.168×10^{7}	1.082×10^{7}	Positive
562	3,4,5-trihydroxy-6-methyloxan-2-yl 2-(methylamino)benzoate	$C_{14}H_{19}NO_{6}$	319.10312	5.581	320.11039	2.352×10^{6}	2.470×10^{6}	2.571×10^{7}	2.772×10^{6}	4.601×10^{7}	2.726×10^{6}	3.440×10^{6}	Positive
563	3-Methylindole	$C_{9}H_{9}N$	131.0739	5.598	132.08124	2.266×10^{7}	4.188×10^{6}	1.572×10^{7}	6.852×10^{6}	1.464×10^{7}	1.072×10^{7}	1.019×10^{7}	Positive
564	4-(2,3-dihydro-1,4-benzodioxin-6-yl)butanoic acid	$C_{12}H_{14}O_{4}$	244.06856	5.98	245.07582	1.676×10^{7}	1.771×10^{7}	4.667×10^{6}	1.291×10^{7}	1.775×10^{7}	9.733×10^{6}	4.638×10^{6}	Positive
565	*L*-beta-Imidazolelactic acid	$C_{6}H_{8}N_{2}O_{3}$	156.05366	1.539	157.06093	2.966×10^{6}	1.895×10^{6}	2.353×10^{7}	2.440×10^{6}	1.775×10^{6}	8.919×10^{6}	4.264×10^{7}	Positive
566	2-Hydroxy-1-(4-methoxyphenyl)propyl hexopyranoside	$C_{16}H_{24}O_{8}$	390.15224	5.512	389.14429	2.175×10^{7}	2.590×10^{7}	3.225×10^{6}	1.595×10^{7}	2.152×10^{6}	1.193×10^{7}	3.128×10^{6}	Negative
567	Docosanoic Acid	$C_{22}H_{44}O_{2}$	340.33458	11.363	339.3273	5.285×10^{6}	8.931×10^{6}	1.332×10^{7}	5.214×10^{6}	3.534×10^{7}	9.696×10^{6}	6.108×10^{6}	Negative
568	3-(3,4-dihydroxyphenyl)propanoic acid	$C_{9}H_{10}O_{4}$	164.04764	6.547	165.05491	5.859×10^{6}	7.075×10^{6}	1.688×10^{7}	1.217×10^{7}	2.321×10^{7}	7.457×10^{6}	1.096×10^{7}	Positive
569	MN-18 N-(5-hydroxypentyl) metabolite	$C_{23}H_{23}N_{3}O_{2}$	373.17347	5.398	374.18072	7.530×10^{5}	6.996×10^{5}	5.355×10^{7}	7.587×10^{5}	1.882×10^{7}	1.058×10^{6}	6.315×10^{6}	Positive
570	N1-(2,3-dihydro-1,4-benzodioxin-2-ylmethyl)-2,2-dimethylpropanamide	$C_{14}H_{19}NO_{3}$	249.13665	5.72	250.14393	2.081×10^{6}	2.080×10^{6}	4.437×10^{7}	1.385×10^{7}	1.173×10^{7}	2.028×10^{6}	5.184×10^{6}	Positive

571	*D*-ribose 5-phosphate	$C_5H_{11}O_8P$	230.01944	1.28	229.01209	7.244×10^{6}	8.103×10^{6}	6.740×10^{6}	1.018×10^{7}	3.946×10^{6}	1.656×10^{7}	2.742×10^{7}	Negative
572	*D*-Xylulose 5-phosphate	$C_5H_{11}O_8P$	184.01376	1.277	229.01196	7.244×10^{6}	8.103×10^{6}	6.740×10^{6}	1.018×10^{7}	3.946×10^{6}	1.656×10^{7}	2.742×10^{7}	Negative
573	Cytidine	$C_9H_{13}N_3O_5$	243.08575	1.461	244.09303	1.638×10^{7}	1.454×10^{7}	6.605×10^{6}	2.357×10^{7}	7.462×10^{6}	5.990×10^{6}	4.876×10^{6}	Positive
574	*L*-arginine	$C_6H_{14}N_4O_2$	174.11194	1.259	173.10466	1.037×10^{7}	1.176×10^{7}	1.412×10^{7}	1.024×10^{7}	5.993×10^{6}	1.683×10^{7}	9.825×10^{6}	Negative
575	N1-(6-methyl-4-oxo-3,4-dihydroquinazolin-2-yl)-4-nitrobenzamide	$C_{16}H_{12}N_4O_4$	324.08818	5.229	323.0809	3.702×10^{6}	3.217×10^{6}	3.223×10^{7}	9.350×10^{6}	2.045×10^{7}	2.720×10^{6}	6.960×10^{6}	Negative
576	Vitamin A	$C_{20}H_{30}O$	286.22958	5.982	287.23681	3.098×10^{6}	3.230×10^{6}	1.445×10^{7}	8.665×10^{6}	3.738×10^{7}	8.377×10^{6}	2.185×10^{6}	Positive
577	2-(tert-butyl)-6,7-dimethoxy-4H-3,1-benzoxazin-4-one	$C_{14}H_{17}NO_4$	263.11586	5.044	264.12317	1.888×10^{7}	1.094×10^{7}	7.635×10^{6}	5.705×10^{6}	1.121×10^{7}	3.984×10^{6}	1.899×10^{7}	Positive
578	1-O-(3,4,5-Trimethoxybenzoyl)-beta-L-galactopyranose	$C_{16}H_{22}O_{10}$	356.10891	5.394	357.11607	1.224×10^{7}	9.769×10^{6}	7.875×10^{6}	7.298×10^{6}	2.319×10^{7}	6.884×10^{6}	9.757×10^{6}	Positive
579	(±)9-HpODE	$C_{18}H_{32}O_4$	312.23034	8.195	311.22306	8.074×10^{6}	7.546×10^{6}	1.330×10^{7}	5.872×10^{6}	3.069×10^{7}	4.557×10^{6}	4.214×10^{6}	Negative
580	Capric acid	$C_{10}H_{20}O_2$	172.14636	5.828	217.14448	9.169×10^{6}	8.760×10^{6}	1.221×10^{7}	1.134×10^{7}	1.158×10^{7}	1.124×10^{7}	9.736×10^{6}	Negative
581	gamma-Glutamylleucine	$C_{11}H_{20}N_2O_5$	260.13724	5.407	259.13004	1.725×10^{7}	2.099×10^{7}	6.021×10^{6}	1.546×10^{7}	7.006×10^{6}	3.079×10^{6}	3.343×10^{6}	Negative
582	Kinetin	$C_{10}H_9N_5O$	237.06392	1.843	238.0712	2.011×10^{7}	1.280×10^{7}	3.910×10^{6}	2.134×10^{7}	2.117×10^{6}	1.567×10^{6}	1.113×10^{7}	Positive
583	3-hydroxy-1,5-diphenylpentan-1-one	$C_{17}H_{18}O_2$	219.08968	5.388	237.12331	3.476×10^{6}	2.688×10^{6}	1.206×10^{7}	5.571×10^{6}	3.569×10^{7}	4.493×10^{6}	8.203×10^{6}	Positive
584	Bisphenol A	$C_{15}H_{16}O_2$	228.11507	5.672	229.12234	8.724×10^{6}	7.597×10^{6}	7.964×10^{6}	1.386×10^{7}	8.060×10^{6}	1.198×10^{7}	1.342×10^{7}	Positive
585	*D*-Gluconic acid	$C_6H_{12}O_7$	196.05851	1.307	197.06583	1.004×10^{7}	9.457×10^{6}	4.886×10^{6}	1.991×10^{7}	8.204×10^{6}	1.071×10^{7}	7.889×10^{6}	Positive
586	4-Hydroxyisoleucine	$C_6H_{13}NO_3$	147.08956	5.099	146.08228	1.026×10^{7}	1.257×10^{7}	8.897×10^{6}	1.211×10^{7}	8.989×10^{6}	9.665×10^{6}	8.536×10^{6}	Negative
587	LPC 16:0	$C_{24}H_{50}NO_7P$	541.33802	9.445	540.33074	2.456×10^{7}	2.935×10^{7}	3.270×10^{6}	9.173×10^{6}	2.887×10^{6}	1.197×10^{6}	5.867×10^{5}	Negative
588	Prostaglandin E2	$C_{20}H_{32}O_5$	352.2253	5.786	397.22348	1.819×10^{7}	1.486×10^{7}	5.772×10^{6}	1.961×10^{7}	6.698×10^{6}	1.300×10^{6}	2.577×10^{6}	Negative
589	PC O-18:3	$C_{26}H_{48}NO_7P$	517.31775	8.366	518.32497	2.597×10^{7}	2.733×10^{7}	4.607×10^{6}	4.837×10^{6}	2.849×10^{6}	1.107×10^{6}	2.294×10^{6}	Positive
590	Docosahexaenoic Acid	$C_{22}H_{32}O_2$	345.26459	8.359	346.27187	1.152×10^{7}	1.110×10^{7}	1.044×10^{7}	9.046×10^{6}	9.965×10^{6}	9.683×10^{6}	7.189×10^{6}	Positive
591	(5S)-5-hydroxy-1,7-diphenylheptan-3-one	$C_{19}H_{22}O_2$	304.14562	4.955	305.15288	7.261×10^{6}	6.685×10^{6}	9.178×10^{6}	1.132×10^{7}	1.004×10^{7}	1.005×10^{7}	1.442×10^{7}	Positive
592	2'-Deoxyinosine 5'-monophosphate	$C_{10}H_{13}N_4O_7P$	332.05423	5.834	331.04695	4.553×10^{6}	3.264×10^{6}	1.482×10^{7}	1.095×10^{7}	5.272×10^{6}	2.313×10^{7}	6.373×10^{6}	Negative
593	Argininosuccinic acid	$C_{10}H_{18}N_4O_6$	290.12217	1.472	291.12973	2.023×10^{7}	1.735×10^{7}	1.065×10^{6}	2.649×10^{7}	1.065×10^{6}	9.290×10^{5}	1.003×10^{6}	Positive
594	dUMP	$C_9H_{13}N_2O_8P$	308.04231	0.522	155.02846	1.111×10^{7}	1.203×10^{7}	9.904×10^{6}	9.789×10^{6}	8.972×10^{6}	8.083×10^{6}	8.057×10^{6}	Positive
595	*L*-Methionine Methyl Ester	$C_6H_{13}NO_2S$	163.06689	1.203	164.07417	1.357×10^{7}	1.099×10^{7}	1.087×10^{7}	6.360×10^{6}	8.182×10^{6}	1.216×10^{7}	5.749×10^{6}	Positive
596	Epigallocatechin	$C_{15}H_{14}O_7$	306.07613	2.263	307.08341	1.566×10^{7}	1.856×10^{7}	8.956×10^{5}	2.430×10^{7}	5.053×10^{6}	1.497×10^{6}	6.615×10^{5}	Positive
597	*P*-Aminohippuric Acid	$C_9H_{10}N_2O_3$	194.06925	5.381	193.0621	2.963×10^{6}	3.511×10^{6}	1.292×10^{7}	7.495×10^{6}	1.165×10^{7}	1.464×10^{7}	1.339×10^{7}	Negative
598	Daidzein	$C_{15}H_{10}O_4$	254.05797	5.854	253.0507	2.915×10^{6}	2.347×10^{6}	3.708×10^{7}	1.210×10^{6}	1.993×10^{7}	9.042×10^{5}	9.940×10^{5}	Negative
599	H-Gly-Pro-OH	$C_7H_{12}N_2O_3$	172.08469	5.225	173.09218	6.021×10^{6}	5.880×10^{6}	9.081×10^{6}	1.108×10^{7}	9.399×10^{6}	1.179×10^{7}	1.059×10^{7}	Positive
600	carbamazepine-d10	$C_{15}H_2[2]H_{10}N_2O$	246.15885	6.743	247.1662	9.822×10^{6}	9.517×10^{6}	8.177×10^{6}	2.057×10^{7}	1.492×10^{6}	8.634×10^{6}	5.297×10^{6}	Positive
601	lipoamide	$C_8H_{15}NOS_2$	205.05879	1.439	206.06611	7.274×10^{6}	6.764×10^{6}	8.232×10^{6}	1.145×10^{7}	1.404×10^{7}	6.491×10^{6}	8.882×10^{6}	Positive

602	(5-methyl-3-isoxazolyl)[4-(5-propyl-2-pyrimidinyl)piperazino]methanone	$C_{16}H_{21}N_5O_2$	298.14118	5.233	316.17523	1.364×10^{6}	1.232×10^{6}	2.534×10^{7}	1.862×10^{6}	2.865×10^{7}	2.721×10^{6}	1.937×10^{6}	Positive
603	alpha-Benzylsuccinic acid	$C_{11}H_{12}O_4$	208.07342	5.31	207.06618	1.025×10^{6}	9.711×10^{5}	1.495×10^{7}	8.999×10^{5}	4.238×10^{7}	9.537×10^{5}	9.439×10^{5}	Negative
604	7-(2-thienyl)[1,2,4]triazolo[4,3-a]pyrimidine	$C_9H_6N_4S$	224.0086	1.274	225.01587	8.476×10^{6}	9.471×10^{6}	5.284×10^{6}	7.664×10^{6}	6.247×10^{6}	1.249×10^{7}	1.245×10^{7}	Positive
605	Serotonin	$C_{10}H_{12}N_2O$	176.09521	5.715	177.10252	9.682×10^{5}	9.225×10^{5}	2.471×10^{7}	2.577×10^{6}	4.129×10^{6}	9.566×10^{6}	1.826×10^{7}	Positive
606	3-Indoleacrylic acid	$C_{11}H_9NO_2$	187.06344	5.321	186.05615	2.777×10^{6}	2.443×10^{6}	2.069×10^{7}	4.902×10^{6}	1.410×10^{7}	6.281×10^{6}	9.577×10^{6}	Negative
607	Lysope 16:0	$C_{21}H_{44}NO_7P$	453.28611	9.422	452.27884	1.955×10^{7}	2.583×10^{7}	3.639×10^{6}	7.284×10^{6}	2.601×10^{6}	1.054×10^{6}	5.708×10^{5}	Negative
608	*O*-Desmethylnaproxen	$C_{13}H_{12}O_3$	216.07888	5.35	217.08615	4.586×10^{6}	5.093×10^{6}	1.250×10^{7}	6.529×10^{6}	2.272×10^{7}	2.902×10^{6}	5.807×10^{6}	Positive
609	Quercetin-3-O-beta-glucopyranosyl-6'-acetate	$C_{23}H_{22}O_{13}$	506.10678	5.56	505.0995	1.084×10^{6}	1.083×10^{6}	6.628×10^{6}	1.312×10^{6}	4.737×10^{7}	1.224×10^{6}	1.129×10^{6}	Negative
610	2-(2-oxo-2-{[2-(2-oxo-1-imidazolidinyl)ethyl]amino}ethoxy)acetic acid	$C_9H_{15}N_3O_5$	245.10044	6.107	246.10769	1.043×10^{7}	1.001×10^{7}	1.765×10^{6}	1.186×10^{7}	4.771×10^{6}	1.582×10^{7}	4.779×10^{6}	Positive
611	LPC 18:2	$C_{26}H_{50}NO_7P$	565.33932	8.88	564.33204	1.837×10^{7}	2.034×10^{7}	6.592×10^{6}	3.853×10^{6}	6.676×10^{6}	9.323×10^{5}	2.441×10^{6}	Negative
612	6,7,8-trimethoxy-2-(2-phenoxy-3-pyridyl)-4H-3,1-benzoxazin-4-one	$C_{22}H_{18}N_2O_6$	444.08008	0.525	445.08734	7.316×10^{6}	1.062×10^{7}	8.560×10^{6}	9.073×10^{6}	7.183×10^{6}	7.903×10^{6}	8.359×10^{6}	Positive
613	Isorhamnetin	$C_{16}H_{12}O_7$	316.05803	6.101	317.0653	1.301×10^{7}	1.151×10^{7}	8.612×10^{6}	9.060×10^{6}	5.996×10^{6}	4.275×10^{6}	6.463×10^{6}	Positive
614	N-(1,3-benzodioxol-5-yl)-2-methyl-5-(piperidinosulfonyl)-3-furamide	$C_{18}H_{20}N_2O_6S$	392.10853	5.721	393.11581	6.855×10^{6}	5.190×10^{6}	1.173×10^{7}	5.752×10^{6}	1.838×10^{7}	8.659×10^{6}	1.651×10^{6}	Positive
615	N-(9-oxodecyl)acetamide	$C_{12}H_{23}NO_2$	213.17299	8.708	236.16218	1.004×10^{7}	1.563×10^{7}	8.126×10^{6}	4.337×10^{6}	6.530×10^{6}	6.573×10^{6}	6.766×10^{6}	Positive
616	Psilocybin	$C_{12}H_{17}N_2O_4P$	284.09248	5.362	283.08526	7.820×10^{6}	1.021×10^{7}	1.963×10^{7}	7.237×10^{6}	4.412×10^{6}	5.715×10^{6}	1.260×10^{6}	Negative
617	Tetradecanedioic acid	$C_{14}H_{26}O_4$	258.18338	7.097	257.17611	9.573×10^{6}	9.950×10^{6}	6.900×10^{6}	8.191×10^{6}	7.224×10^{6}	8.768×10^{6}	5.324×10^{6}	Negative
618	UDP-N-acetylglucosamine	$C_{17}H_{27}N_3O_{17}P_2$	607.08289	1.306	606.07553	8.891×10^{6}	8.793×10^{6}	8.013×10^{6}	7.734×10^{6}	9.027×10^{6}	6.325×10^{6}	6.634×10^{6}	Negative
619	FAHFA 16:0/18:2	$C_{34}H_{62}O_4$	534.46234	10.477	533.45506	7.568×10^{6}	8.905×10^{6}	8.839×10^{6}	4.530×10^{6}	1.007×10^{7}	9.175×10^{6}	5.914×10^{6}	Negative
620	*D*-Panthenol	$C_9H_{19}NO_4$	205.13166	5.11	206.13895	1.799×10^{7}	6.650×10^{6}	1.368×10^{7}	1.070×10^{7}	1.118×10^{6}	2.565×10^{6}	1.670×10^{6}	Positive
621	1-benzoyl-1,2-dihydroquinoline-2-carbonitrile	$C_{17}H_{12}N_2O$	260.09527	5.985	259.088	1.190×10^{6}	9.775×10^{5}	2.467×10^{7}	2.593×10^{6}	1.941×10^{7}	1.811×10^{6}	3.576×10^{6}	Negative
622	N-morpholino-N'-[(5-nitro-3-thienyl)carbonyl]urea	$C_{10}H_{12}N_4O_5S$	300.05095	8.571	301.05823	1.030×10^{7}	6.296×10^{6}	1.006×10^{7}	8.927×10^{6}	3.119×10^{6}	8.415×10^{6}	6.802×10^{6}	Positive
623	Deoxyguanosine	$C_{10}H_{13}N_5O_4$	267.09548	1.279	268.1029	8.431×10^{6}	9.317×10^{6}	5.202×10^{6}	1.438×10^{7}	4.519×10^{6}	2.800×10^{6}	8.225×10^{6}	Positive
624	2,3-Dinor-TXB2	$C_{18}H_{30}O_6$	342.20361	6.191	343.21092	1.240×10^{7}	9.247×10^{6}	4.102×10^{6}	1.021×10^{7}	2.728×10^{6}	8.814×10^{6}	5.263×10^{6}	Positive
625	2-[amino(3-chloroanilino)methylene]malononitrile	$C_{10}H_7ClN_4$	196.0585	1.567	197.06596	8.932×10^{6}	8.489×10^{6}	4.571×10^{6}	7.404×10^{6}	8.450×10^{6}	8.364×10^{6}	6.270×10^{6}	Positive
626	5,7-dimethyl-2-phenylpyrazolo[1,5-a]pyrimidine	$C_{14}H_{13}N_3$	223.1056	1.263	224.11288	5.428×10^{6}	5.448×10^{6}	1.164×10^{7}	5.815×10^{6}	7.100×10^{6}	3.923×10^{6}	1.305×10^{7}	Positive
627	*Dl*-Glyceraldehyde3-phosphate	$C_3H_7O_6P$	169.9984	1.217	171.00568	9.044×10^{6}	1.088×10^{7}	6.929×10^{6}	7.035×10^{6}	8.052×10^{6}	4.718×10^{6}	5.628×10^{6}	Positive
628	SPB 16:1;2O	$C_{16}H_{33}NO_2$	271.2513	6.676	272.25861	6.122×10^{6}	8.326×10^{6}	5.537×10^{6}	7.791×10^{6}	8.135×10^{6}	1.063×10^{7}	5.046×10^{6}	Positive
629	N1-(1H-indol-4-yl)cyclohexane-1-carboxamide	$C_{15}H_{18}N_2O$	280.09957	4.965	281.10685	3.854×10^{6}	3.268×10^{6}	7.847×10^{6}	7.663×10^{6}	1.152×10^{7}	9.611×10^{6}	7.759×10^{6}	Positive

630	Kynurenine	$C_{10}H_{12}N_2O_3$	208.08455	5.537	209.09249	2.092×10^{6}	3.555×10^{6}	2.822×10^{7}	8.402×10^{6}	4.879×10^{6}	2.316×10^{6}	1.806×10^{6}	Positive
631	(4-isobutoxyphenyl)(phenyl)methanone oxime	$C_{17}H_{19}NO_2$	269.14169	6.598	270.14896	2.011×10^{6}	1.149×10^{6}	1.723×10^{7}	3.479×10^{6}	1.975×10^{7}	2.063×10^{6}	5.428×10^{6}	Positive
632	4,5-diphenyl-2,3-dihydro-1H-pyrazolo[3,4-c]pyridazin-3-one	$C_{17}H_{12}N_4O$	288.10327	5.444	287.09599	2.751×10^{7}	1.406×10^{7}	1.125×10^{6}	3.438×10^{6}	1.066×10^{6}	1.113×10^{6}	2.007×10^{6}	Negative
633	Nervonic acid	$C_{24}H_{46}O_2$	366.35023	10.214	365.34296	1.730×10^{6}	1.867×10^{6}	2.864×10^{6}	2.708×10^{6}	2.283×10^{7}	1.655×10^{7}	1.757×10^{6}	Negative
634	6β-Prostaglandin I1	$C_{20}H_{34}O_5$	400.24472	6.398	399.23743	4.120×10^{6}	4.023×10^{6}	1.283×10^{7}	5.431×10^{6}	2.020×10^{7}	1.162×10^{6}	2.480×10^{6}	Negative
635	*α*-D-Glucose-1,6-bisphosphate	$C_6H_{14}O_{12}P_2$	169.99748	6.43	338.98835	1.033×10^{7}	1.566×10^{7}	5.032×10^{6}	4.645×10^{6}	5.496×10^{6}	4.159×10^{6}	4.622×10^{6}	Negative
636	LPA 18:3	$C_{21}H_{37}O_7P$	432.22815	8.959	431.22088	1.542×10^{7}	1.711×10^{7}	7.804×10^{6}	4.166×10^{6}	2.870×10^{6}	1.186×10^{6}	1.006×10^{6}	Negative
637	(3R)-4,4-Dimethyl-2-oxotetrahydro-3-furanyl beta-D-glucopyranoside	$C_{12}H_{20}O_8$	292.11582	1.456	293.12306	1.138×10^{6}	1.216×10^{6}	9.264×10^{6}	3.015×10^{6}	1.069×10^{7}	1.098×10^{7}	1.301×10^{7}	Positive
638	N1-[4-(trifluoromethyl)phenyl]-2-phenylbutanamide	$C_{17}H_{16}F_3NO$	285.13647	6.569	308.12574	8.495×10^{5}	8.663×10^{5}	2.536×10^{7}	1.553×10^{6}	1.498×10^{7}	9.743×10^{5}	4.062×10^{6}	Positive
639	*L*-Cysteine-glutathione gisulfide	$C_{13}H_{22}N_4O_8S_2$	426.08808	6.417	425.08081	6.457×10^{6}	7.553×10^{6}	7.877×10^{6}	5.445×10^{6}	6.535×10^{6}	6.225×10^{6}	8.351×10^{6}	Negative
640	*DL*-Panthenol	$C_9H_{19}NO_4$	205.13159	5.919	206.13884	8.025×10^{6}	7.027×10^{6}	7.724×10^{6}	6.885×10^{6}	1.176×10^{7}	3.195×10^{6}	3.526×10^{6}	Positive
641	Oxoadipic Acid	$C_6H_8O_5$	160.03763	0.515	161.04477	5.889×10^{6}	6.350×10^{6}	6.210×10^{6}	7.037×10^{6}	7.151×10^{6}	7.018×10^{6}	8.364×10^{6}	Positive
642	LPE 18:3	$C_{23}H_{42}NO_7P$	475.2702	8.569	474.26293	2.070×10^{7}	2.058×10^{7}	1.899×10^{6}	2.998×10^{6}	6.119×10^{5}	4.921×10^{5}	5.399×10^{5}	Negative
643	15-OxoEDE	$C_{20}H_{34}O_3$	368.2715	9.957	367.26422	6.809×10^{6}	7.345×10^{6}	7.323×10^{6}	6.702×10^{6}	7.138×10^{6}	5.787×10^{6}	6.523×10^{6}	Negative
644	6*α*-Prostaglandin I1	$C_{20}H_{34}O_5$	336.22904	7.56	381.22675	7.061×10^{6}	6.228×10^{6}	4.257×10^{6}	4.561×10^{6}	1.346×10^{7}	7.458×10^{6}	3.906×10^{6}	Negative
645	3-(2-Naphthyl)-D-Alanine	$C_{13}H_{13}NO_2$	215.09507	5.438	216.10235	7.440×10^{6}	6.956×10^{6}	6.583×10^{6}	6.639×10^{6}	6.876×10^{6}	5.300×10^{6}	7.069×10^{6}	Positive
646	Gatifloxacin	$C_{19}H_{22}FN_3O_4$	397.13543	5.326	398.1427	3.236×10^{6}	4.007×10^{6}	1.171×10^{7}	4.794×10^{6}	5.956×10^{6}	8.331×10^{6}	8.573×10^{6}	Positive
647	13-Hpotre(R)	$C_{18}H_{30}O_4$	310.21469	7.802	309.20741	4.175×10^{6}	2.697×10^{6}	6.428×10^{6}	2.314×10^{6}	2.882×10^{7}	1.143×10^{6}	1.006×10^{6}	Negative
648	N2-ethyl-N4-isopropyl-6-(methylthio)-1,3,5-triazine-2,4-diamine	$C_9H_{17}N_5S$	227.12157	6.072	228.12885	4.821×10^{6}	4.126×10^{6}	7.129×10^{6}	6.773×10^{6}	1.006×10^{7}	7.038×10^{6}	6.527×10^{6}	Positive
649	*L*-Ornithine	$C_5H_{12}N_2O_2$	132.08994	1.264	131.08266	9.944×10^{6}	9.303×10^{6}	6.087×10^{6}	1.031×10^{7}	1.992×10^{6}	5.244×10^{6}	3.472×10^{6}	Negative
650	Glycyl-L-leucine	$C_8H_{16}N_2O_3$	188.11614	5.223	189.12342	1.002×10^{7}	9.236×10^{6}	4.064×10^{6}	1.372×10^{7}	2.149×10^{6}	2.548×10^{6}	4.575×10^{6}	Positive
651	Deoxyribose 5-Phosphate	$C_5H_{11}O_7P$	214.02467	1.224	215.03194	8.461×10^{6}	8.387×10^{6}	6.103×10^{6}	7.882×10^{6}	7.005×10^{6}	3.977×10^{6}	4.303×10^{6}	Positive
652	*Dl*-Indole-3-lactic acid	$C_{11}H_{11}NO_3$	205.07381	5.289	223.10779	7.262×10^{6}	8.117×10^{6}	5.221×10^{6}	1.067×10^{7}	3.525×10^{6}	4.231×10^{6}	5.597×10^{6}	Positive
653	{4-[4-(2-methoxyphenyl)piperidino]-3-nitrophenyl}methanol	$C_{19}H_{22}N_2O_4$	342.15786	6.061	343.16501	7.169×10^{5}	7.041×10^{5}	2.284×10^{7}	7.226×10^{5}	1.218×10^{7}	8.429×10^{5}	6.592×10^{6}	Positive
654	N-Acetyl-D-tryptophan	$C_{13}H_{14}N_2O_3$	246.10072	5.42	247.108	7.173×10^{6}	7.265×10^{6}	6.827×10^{6}	6.005×10^{6}	7.351×10^{6}	5.964×10^{6}	3.890×10^{6}	Positive
655	5-methoxy-8,8-dimethyl-2-phenyl-4H,8H-pyrano[2,3-h]chromen-4-one	$C_{21}H_{18}O_4$	334.11663	5.025	335.12391	3.426×10^{6}	2.658×10^{6}	9.607×10^{6}	6.778×10^{6}	5.036×10^{6}	3.469×10^{6}	1.341×10^{7}	Positive
656	23-Nordeoxycholic acid	$C_{23}H_{38}O_4$	378.27519	11.483	377.26792	7.848×10^{6}	7.250×10^{6}	4.341×10^{6}	4.293×10^{6}	3.849×10^{6}	7.521×10^{6}	9.266×10^{6}	Negative
657	Levalbuterol	$C_{13}H_{21}NO_3$	261.13618	5.734	262.14336	2.171×10^{6}	2.505×10^{6}	1.639×10^{7}	6.454×10^{6}	4.010×10^{6}	3.413×10^{6}	8.832×10^{6}	Positive
658	Ethyl sorbate	$C_8H_{12}O_2$	140.08115	5.903	141.08843	8.107×10^{6}	7.525×10^{6}	5.375×10^{6}	6.443×10^{6}	5.611×10^{6}	5.378×10^{6}	5.287×10^{6}	Positive

659	Thromboxane B2	$C_{20}H_{34}O_6$	370.23588	6.254	351.21805	1.071×10^{7}	1.065×10^{7}	3.551×10^{6}	1.154×10^{7}	4.583×10^{6}	1.072×10^{6}	1.118×10^{6}	Negative
660	N-Methylhydantoin	$C_4H_6N_2O_2$	114.04277	1.286	113.03567	1.253×10^{7}	1.204×10^{7}	1.318×10^{6}	1.295×10^{7}	1.102×10^{6}	1.407×10^{6}	1.172×10^{6}	Negative
661	Isobutyryl carnitine	$C_{11}H_{21}NO_4$	231.14718	5.499	232.15446	4.093×10^{6}	3.629×10^{6}	4.876×10^{6}	5.175×10^{6}	1.769×10^{7}	3.910×10^{6}	3.129×10^{6}	Positive
662	Phlorobenzophenone	$C_{13}H_{10}O_4$	230.05793	5.867	229.05066	5.108×10^{6}	5.803×10^{6}	1.990×10^{7}	9.066×10^{6}	8.925×10^{5}	8.481×10^{5}	8.598×10^{5}	Negative
663	3,3,5-trimethyl-3H,11H-pyrano[3,2-a]carbazole	$C_{18}H_{17}NO$	263.13115	7.094	264.13843	5.060×10^{5}	5.498×10^{5}	2.951×10^{7}	5.442×10^{5}	7.556×10^{6}	4.525×10^{5}	3.137×10^{6}	Positive
664	DG O-22:3_22:2	$C_{47}H_{84}O_4$	729.6623	11.551	730.66958	9.346×10^{6}	8.581×10^{6}	2.386×10^{6}	6.079×10^{6}	7.938×10^{5}	6.040×10^{6}	8.898×10^{6}	Positive
665	N2-Methylguanosine	$C_{11}H_{15}N_5O_5$	297.10746	4.906	298.11471	8.295×10^{6}	8.013×10^{6}	3.308×10^{6}	8.548×10^{6}	4.446×10^{6}	5.460×10^{6}	3.710×10^{6}	Positive
666	4-Guanidinobutanoic acid	$C_5H_{11}N_3O_2$	145.08513	1.514	146.09241	7.838×10^{6}	6.130×10^{6}	4.402×10^{6}	8.450×10^{6}	7.244×10^{6}	3.025×10^{6}	4.655×10^{6}	Positive
667	6-Keto-prostaglandin f1alpha	$C_{20}H_{34}O_6$	370.23526	5.736	415.23343	6.932×10^{6}	1.095×10^{7}	3.364×10^{6}	8.840×10^{6}	5.143×10^{6}	3.858×10^{6}	2.636×10^{6}	Negative
668	16(R)-HETE	$C_{20}H_{32}O_3$	342.21492	8.891	343.2222	5.310×10^{6}	5.122×10^{6}	6.320×10^{6}	4.343×10^{6}	1.292×10^{7}	3.878×10^{6}	3.368×10^{6}	Positive
669	Glu-Glu	$C_{10}H_{16}N_2O_7$	276.09564	1.285	259.09232	7.472×10^{6}	6.893×10^{6}	2.758×10^{6}	1.241×10^{7}	5.888×10^{6}	1.652×10^{6}	4.189×10^{6}	Positive
670	*trans*-Petroselinic Acid	$C_{18}H_{34}O_2$	328.26164	9.052	327.25436	1.450×10^{7}	1.638×10^{7}	2.198×10^{6}	4.219×10^{6}	1.548×10^{6}	1.541×10^{6}	7.510×10^{5}	Negative
671	Estrone	$C_{18}H_{22}O_2$	292.14399	6.389	293.15126	1.808×10^{7}	1.246×10^{7}	6.409×10^{5}	7.025×10^{6}	1.621×10^{6}	6.311×10^{5}	6.646×10^{5}	Positive
672	19(R)-Hydroxy prostaglandin F2α	$C_{20}H_{34}O_6$	352.22546	6.665	397.2237	1.095×10^{7}	1.034×10^{7}	6.284×10^{6}	8.127×10^{6}	3.698×10^{6}	1.000×10^{6}	6.714×10^{5}	Negative
673	6β-Oxycodol N-oxide	$C_{18}H_{23}NO_5$	311.17411	6.187	334.16342	3.889×10^{6}	3.512×10^{6}	8.564×10^{6}	3.669×10^{6}	8.585×10^{6}	5.736×10^{6}	7.016×10^{6}	Positive
674	Phe-Pro	$C_{14}H_{18}N_2O_3$	262.13223	5.647	569.26208	9.097×10^{5}	9.838×10^{5}	1.017×10^{7}	2.236×10^{6}	1.536×10^{7}	3.746×10^{6}	7.353×10^{6}	Negative
675	CLOFAZIMINE	$C_{27}H_{22}Cl_2N_4$	472.12016	4.889	473.12744	4.397×10^{6}	3.138×10^{6}	5.758×10^{6}	3.964×10^{6}	4.168×10^{6}	7.454×10^{6}	1.185×10^{7}	Positive
676	N-METHYL (-)EPHEDRINE	$C_{11}H_{17}NO$	179.13119	5.528	180.13853	2.586×10^{6}	2.665×10^{6}	6.616×10^{6}	5.741×10^{6}	1.170×10^{7}	6.394×10^{6}	4.663×10^{6}	Positive
677	8-(1,2-dihydroxy-3-methylbut-3-en-1-yl)-7-methoxy-2H-chromen-2-one	$C_{15}H_{16}O_5$	254.11618	5.957	277.10538	1.268×10^{6}	1.173×10^{6}	1.724×10^{7}	6.510×10^{6}	8.425×10^{6}	2.622×10^{6}	3.023×10^{6}	Positive
678	N4-Acetylcytidine	$C_{11}H_{15}N_3O_6$	285.10024	5.865	284.09296	1.128×10^{6}	9.002×10^{5}	1.199×10^{7}	2.308×10^{6}	5.417×10^{6}	4.848×10^{6}	1.360×10^{7}	Negative
679	N6-Succinyl Adenosine	$C_{14}H_{17}N_5O_8$	383.10764	5.08	384.11487	8.756×10^{6}	7.987×10^{6}	4.167×10^{6}	7.712×10^{6}	5.162×10^{6}	3.488×10^{6}	2.668×10^{6}	Positive
680	*L*-Leucyl-L-Alanine	$C_9H_{18}N_2O_3$	202.13189	5.104	203.13917	9.189×10^{6}	7.384×10^{6}	2.430×10^{6}	1.368×10^{7}	1.617×10^{6}	1.934×10^{6}	3.615×10^{6}	Positive
681	4-Aminoindole	$C_8H_8N_2$	132.06837	8.238	287.12573	4.446×10^{6}	4.289×10^{6}	6.571×10^{6}	6.277×10^{6}	9.089×10^{6}	5.215×10^{6}	3.898×10^{6}	Positive
682	4-[4-(trifluoromethoxy)anilino]-2H-chromen-2-one	$C_{16}H_{10}F_3NO_3$	321.06333	1.465	322.07028	9.803×10^{6}	8.659×10^{6}	1.635×10^{6}	1.133×10^{7}	1.848×10^{6}	4.592×10^{6}	1.517×10^{6}	Positive
683	PC O-18:2	$C_{26}H_{50}NO_7P$	519.33347	8.868	542.32273	1.300×10^{7}	1.443×10^{7}	3.249×10^{6}	2.695×10^{6}	3.812×10^{6}	7.192×10^{5}	1.281×10^{6}	Positive
684	Stearoyl Ethanolamide	$C_{20}H_{41}NO_2$	309.30324	11.336	310.31052	6.094×10^{6}	6.188×10^{6}	5.378×10^{6}	4.607×10^{6}	5.574×10^{6}	5.036×10^{6}	5.599×10^{6}	Positive
685	Senecionine	$C_{18}H_{25}NO_5$	335.16667	5.926	336.17394	1.477×10^{6}	1.523×10^{6}	2.318×10^{6}	1.728×10^{6}	2.296×10^{7}	3.939×10^{6}	4.394×10^{6}	Positive
686	acetyl phospahte	$C_2H_5O_5P$	139.98786	1.223	178.95101	6.624×10^{6}	6.896×10^{6}	4.680×10^{6}	7.243×10^{6}	6.360×10^{6}	2.793×10^{6}	3.234×10^{6}	Positive
687	2,3-Dinor-8-epi-prostaglandin F2α	$C_{18}H_{30}O_5$	326.20979	6.702	325.20253	6.193×10^{6}	4.263×10^{6}	7.499×10^{6}	3.903×10^{6}	8.223×10^{6}	4.104×10^{6}	3.458×10^{6}	Negative
688	Diosmetin	$C_{16}H_{12}O_6$	300.06356	5.49	301.07084	1.236×10^{7}	1.337×10^{7}	1.501×10^{6}	5.076×10^{6}	1.275×10^{6}	2.941×10^{6}	1.089×10^{6}	Positive
689	3,5,7-trihydroxy-2-phenyl-4H-chromen-4-one	$C_{15}H_{10}O_5$	270.05296	6.125	271.06024	4.428×10^{6}	5.399×10^{6}	8.237×10^{6}	5.265×10^{6}	5.623×10^{6}	5.713×10^{6}	2.884×10^{6}	Positive

690	13,14-Dihydro-15-keto Prostaglandin E1	$C_{20}H_{34}O_5$	354.24062	6.368	353.23377	1.170×10^{7}	8.948×10^{6}	1.559×10^{6}	1.149×10^{7}	2.716×10^{6}	5.745×10^{5}	5.533×10^{5}	Negative
691	Phenylacetylglutamine	$C_{13}H_{16}N_2O_4$	264.11139	5.013	309.10964	6.554×10^{6}	5.766×10^{6}	9.296×10^{6}	4.735×10^{6}	3.827×10^{6}	2.204×10^{6}	4.385×10^{6}	Negative
692	Isorhapontigenin	$C_{15}H_{14}O_4$	258.08946	6.285	257.08218	8.344×10^{6}	9.100×10^{6}	4.291×10^{6}	3.304×10^{6}	6.511×10^{6}	4.028×10^{6}	9.592×10^{5}	Negative
693	Asp-Phe	$C_{13}H_{16}N_2O_5$	280.10588	5.376	263.10254	3.179×10^{6}	3.302×10^{6}	6.306×10^{6}	4.341×10^{6}	6.797×10^{6}	4.861×10^{6}	7.511×10^{6}	Positive
694	5-Methyl-2-[(3S)-1-(2-pyridinylmethyl)-3-pyrrolidinyl]-1,3-benzoxazole	$C_{18}H_{19}N_3O$	293.1527	5.648	294.15998	1.263×10^{6}	1.255×10^{6}	1.374×10^{7}	1.383×10^{6}	1.539×10^{7}	1.587×10^{6}	1.408×10^{6}	Positive
695	Flavanone	$C_{15}H_{12}O_2$	224.08409	6.713	223.07681	4.440×10^{5}	4.822×10^{5}	2.098×10^{7}	6.869×10^{5}	1.201×10^{7}	4.162×10^{5}	9.074×10^{5}	Negative
696	2-Ethylhexanoic acid	$C_8H_{16}O_2$	144.11511	5.692	143.10786	7.709×10^{6}	1.002×10^{7}	3.501×10^{6}	6.169×10^{6}	3.706×10^{6}	1.580×10^{6}	1.558×10^{6}	Negative
697	3-Acetyl-2,5-dimethylfuran	$C_8H_{10}O_2$	138.06555	5.676	139.07281	2.912×10^{6}	2.799×10^{6}	6.396×10^{6}	5.308×10^{6}	5.247×10^{6}	3.437×10^{6}	8.063×10^{6}	Positive
698	*L*-Alanyl-L-Lysine	$C_9H_{19}N_3O_3$	217.14165	1.223	457.27196	6.177×10^{6}	6.167×10^{6}	4.651×10^{6}	5.972×10^{6}	4.946×10^{6}	2.887×10^{6}	3.352×10^{6}	Positive
699	5-Methyl-dl-tryptophan	$C_{12}H_{14}N_2O_2$	218.10556	5.572	263.10394	1.834×10^{6}	1.725×10^{6}	5.081×10^{6}	3.351×10^{6}	7.117×10^{6}	8.088×10^{6}	6.609×10^{6}	Negative
700	Fosfomycin	$C_3H_7O_4P$	138.00882	1.114	277.02509	4.779×10^{6}	4.930×10^{6}	4.598×10^{6}	5.808×10^{6}	4.077×10^{6}	4.858×10^{6}	4.721×10^{6}	Positive
701	IDP	$C_{10}H_{14}N_4O_{11}P_2$	428.01208	5.981	427.00481	3.528×10^{6}	4.677×10^{6}	3.806×10^{6}	8.209×10^{6}	3.386×10^{6}	3.674×10^{6}	6.124×10^{6}	Negative
702	2'-O-Methyladenosine	$C_{11}H_{15}N_5O_4$	281.11269	4.892	282.12011	6.448×10^{6}	6.046×10^{6}	3.913×10^{6}	7.409×10^{6}	4.295×10^{6}	2.957×10^{6}	2.331×10^{6}	Positive
703	LPA 18:2	$C_{21}H_{39}O_7P$	434.24412	9.662	433.23684	7.192×10^{6}	7.913×10^{6}	6.240×10^{6}	3.269×10^{6}	3.698×10^{6}	2.062×10^{6}	1.764×10^{6}	Negative
704	bicyclo[2.2.2]oct-2-en-1-yl 4-methylbenzene-1-sulfonate	$C_{15}H_{18}O_3S$	278.10034	5.593	279.10717	2.540×10^{6}	2.825×10^{6}	6.825×10^{6}	2.804×10^{6}	5.022×10^{6}	4.400×10^{6}	7.416×10^{6}	Positive
705	(1E,4E)-1,5-bis(4-methoxyphenyl)penta-1,4-dien-3-one	$C_{19}H_{18}O_3$	316.10523	5.524	317.1125	2.029×10^{6}	2.241×10^{6}	2.062×10^{6}	2.673×10^{6}	2.554×10^{6}	9.478×10^{6}	1.071×10^{7}	Positive
706	SNH	$C_{13}H_{20}N_6O_6$	356.14541	5.354	357.15268	5.543×10^{6}	7.504×10^{6}	3.509×10^{6}	1.122×10^{7}	1.682×10^{6}	1.146×10^{6}	9.733×10^{5}	Positive
707	Bz-RS-ISer(3-Ph)-Ome	$C_{17}H_{17}NO_4$	299.11608	5.53	300.12332	3.840×10^{6}	3.467×10^{6}	2.892×10^{6}	4.188×10^{6}	6.866×10^{6}	4.434×10^{6}	5.561×10^{6}	Positive
708	Albendazole sulfone	$C_{12}H_{15}N_3O_4S$	297.08295	4.893	298.09022	1.112×10^{7}	8.292×10^{6}	4.161×10^{5}	1.007×10^{7}	4.932×10^{5}	3.967×10^{5}	4.040×10^{5}	Positive
709	NVP-231	$C_{25}H_{25}N_3O_2S$	431.16689	5.112	432.17416	9.568×10^{5}	9.462×10^{5}	1.115×10^{6}	1.103×10^{6}	1.128×10^{6}	1.150×10^{6}	2.423×10^{7}	Positive
710	5-Methylcytosine	$C_5H_7N_3O$	125.05934	1.433	126.0666	1.671×10^{6}	2.936×10^{6}	3.432×10^{6}	5.271×10^{6}	1.495×10^{7}	1.106×10^{6}	1.153×10^{6}	Positive
711	(4-nitrophenyl)(2,3,4,5,6-pentamethylphenyl)methanone	$C_{18}H_{19}NO_3$	297.13626	6.427	298.14348	1.809×10^{6}	1.723×10^{6}	1.165×10^{7}	1.602×10^{6}	1.037×10^{7}	1.484×10^{6}	1.876×10^{6}	Positive
712	LPC 18:3-SN1	$C_{26}H_{48}NO_7P$	517.31766	8.565	518.32493	1.367×10^{7}	1.087×10^{7}	7.112×10^{5}	3.175×10^{6}	6.095×10^{5}	6.451×10^{5}	6.327×10^{5}	Positive
713	Ureidosuccinic acid	$C_5H_8N_2O_5$	176.04345	2.24	175.03617	8.845×10^{5}	8.932×10^{5}	6.191×10^{6}	9.101×10^{5}	9.724×10^{5}	1.144×10^{6}	1.921×10^{7}	Negative
714	2-(Dimethylamino)Guanosine	$C_{12}H_{17}N_5O_5$	311.12284	5.019	312.13031	4.435×10^{6}	4.728×10^{6}	3.430×10^{6}	5.505×10^{6}	8.522×10^{6}	3.064×10^{6}	4.963×10^{5}	Positive
715	LPE 18:2	$C_{23}H_{44}NO_7P$	477.28584	9.084	476.27856	1.150×10^{7}	1.248×10^{7}	2.470×10^{6}	1.342×10^{6}	1.132×10^{6}	5.125×10^{5}	6.002×10^{5}	Negative
716	1,4-Dihydro-1-Methyl-4-Oxo-3-Pyridinecarboxamide	$C_7H_8N_2O_2$	152.05886	1.253	170.09269	4.012×10^{6}	5.502×10^{6}	3.221×10^{6}	3.741×10^{6}	1.931×10^{6}	6.487×10^{6}	5.036×10^{6}	Positive
717	Norbuprenorphine	$C_{25}H_{35}NO_4$	413.262	9.704	396.25851	4.523×10^{6}	5.200×10^{6}	4.275×10^{6}	3.482×10^{6}	4.216×10^{6}	3.669×10^{6}	3.956×10^{6}	Positive
718	Dithranol	$C_{14}H_{10}O_3$	226.06333	1.128	227.07061	3.591×10^{6}	3.760×10^{6}	5.014×10^{6}	4.373×10^{6}	4.404×10^{6}	4.202×10^{6}	3.950×10^{6}	Positive
719	LPC 18:1	$C_{26}H_{52}NO_7P$	567.35506	9.452	566.34779	6.245×10^{6}	6.716×10^{6}	5.416×10^{6}	2.382×10^{6}	3.278×10^{6}	1.237×10^{6}	3.558×10^{6}	Negative
720	*L*-Cystathionine	$C_7H_{14}N_2O_4S$	222.06741	5.551	223.07469	2.769×10^{6}	1.302×10^{6}	1.412×10^{6}	3.467×10^{6}	8.822×10^{6}	7.227×10^{6}	3.757×10^{6}	Positive

721	Gibberellin A4	$C_{19}H_{24}O_5$	332.1609	6.673	377.15908	4.061×10^6	4.351×10^6	4.756×10^6	4.039×10^6	3.949×10^6	3.699×10^6	3.890×10^6	Negative
722	5-Methyluridine	$C_{10}H_{14}N_2O_6$	258.08535	4.826	257.07801	4.722×10^6	5.153×10^6	2.817×10^6	6.380×10^6	2.929×10^6	2.763×10^6	3.584×10^6	Negative
723	*DL*-2-(acetylamino)-3-phenylpropanoic acid	$C_{11}H_{13}NO_3$	224.11637	4.831	225.12364	2.976×10^6	4.126×10^6	3.021×10^6	3.781×10^6	3.363×10^6	3.345×10^6	7.695×10^6	Positive
724	4-oxo-5-phenylpentanoic acid	$C_{11}H_{12}O_3$	214.05652	1.764	215.0638	7.028×10^5	7.031×10^5	5.476×10^6	6.464×10^5	6.235×10^5	3.419×10^6	1.606×10^7	Positive
725	Taxifolin	$C_{15}H_{12}O_7$	304.05605	5.038	305.06332	4.749×10^6	6.574×10^6	6.850×10^6	7.480×10^6	7.081×10^5	4.857×10^5	5.535×10^5	Positive
726	5-ethoxy-2-[(2,3,4,5,6-pentafluorobenzyl)thio]-1H-benzo[d]imidazole	$C_{16}H_{11}F_5N_2OS$	396.03451	7.598	397.04179	4.672×10^6	4.135×10^6	5.063×10^6	4.965×10^6	1.921×10^6	3.717×10^6	2.824×10^6	Positive
727	Methylbenzethonium chloride	$C_{28}H_{43}NO_2$	426.33253	8.296	427.3398	7.526×10^6	5.963×10^6	9.523×10^5	3.005×10^6	5.367×10^5	3.473×10^6	5.791×10^6	Positive
728	(2E)-6-hydroxy-2-methyl-6-(4-methylphenyl)hept-2-enoic acid	$C_{15}H_{20}O_3$	230.12683	5.162	231.13411	1.859×10^6	1.760×10^6	6.103×10^6	2.623×10^6	8.859×10^6	3.437×10^6	2.356×10^6	Positive
729	Ribulose-5-phosphate	$C_5H_{11}O_8P$	230.01966	1.221	231.027	4.636×10^6	5.146×10^6	3.654×10^6	4.579×10^6	4.090×10^6	2.150×10^6	2.526×10^6	Positive
730	Poncirin	$C_{28}H_{34}O_{14}$	594.19385	5.064	595.20113	2.307×10^6	2.433×10^6	1.682×10^7	2.873×10^6	5.325×10^5	5.556×10^5	1.260×10^6	Positive
731	4-butyl-4-(hydroxymethyl)-1,2-diphenylpyrazolidine-3,5-dione	$C_{20}H_{22}N_2O_3$	338.16036	7.056	339.16764	1.716×10^6	1.724×10^6	1.045×10^7	1.689×10^6	6.614×10^6	1.500×10^6	2.891×10^6	Positive
732	*D*-Ala-D-Ala	$C_6H_{12}N_2O_3$	160.08495	1.337	161.09223	1.836×10^6	1.966×10^6	6.835×10^6	6.725×10^6	2.774×10^6	3.673×10^6	2.554×10^6	Positive
733	FAHFA 18:1/20:3	$C_{38}H_{66}O_4$	586.49464	10.682	585.48736	2.180×10^6	2.239×10^6	3.583×10^6	9.528×10^5	1.234×10^7	3.096×10^6	1.904×10^6	Negative
734	1,7-bis(4-hydroxyphenyl)heptan-3-one	$C_{19}H_{22}O_3$	280.14218	5.455	281.14918	2.317×10^6	2.467×10^6	8.218×10^6	2.989×10^6	3.578×10^6	2.473×10^6	4.112×10^6	Positive
735	4-(octyloxy)benzoic acid	$C_{15}H_{22}O_3$	250.15519	6.855	295.15324	3.871×10^6	4.313×10^6	3.455×10^6	4.187×10^6	2.300×10^6	3.774×10^6	4.037×10^6	Negative
736	*L*-Hydroxyproline	$C_5H_9NO_3$	131.05831	1.292	130.05105	5.569×10^6	6.444×10^6	2.344×10^6	5.867×10^6	1.192×10^6	2.047×10^6	2.267×10^6	Negative
737	Ursolic acid	$C_{30}H_{48}O_3$	456.36087	9.79	455.3536	6.825×10^6	8.795×10^6	7.381×10^5	2.170×10^6	7.213×10^5	5.604×10^6	7.003×10^5	Negative
738	Tryptophanol	$C_{11}H_{14}N_2O$	190.11079	5.927	191.11806	1.616×10^6	1.666×10^6	8.678×10^6	1.890×10^6	2.535×10^6	4.309×10^6	4.806×10^6	Positive
739	Alternariol	$C_{14}H_{10}O_5$	258.05019	5.872	259.05746	1.052×10^6	4.198×10^6	5.170×10^6	7.144×10^6	2.360×10^6	2.836×10^6	2.515×10^6	Positive
740	Linolelaidic Acid (C18:2N6T)	$C_{18}H_{32}O_2$	280.24028	10.275	279.233	3.482×10^6	4.044×10^6	3.508×10^6	3.190×10^6	4.774×10^6	3.075×10^6	3.089×10^6	Negative
741	3-morpholino-5,6-diphenylpyridazine-4-carbonitrile	$C_{21}H_{18}N_4O$	342.14464	6.136	343.15192	1.520×10^6	1.564×10^6	4.013×10^6	2.068×10^6	1.317×10^7	1.420×10^6	1.379×10^6	Positive
742	Estriol	$C_{18}H_{24}O_3$	288.1726	6.286	289.17979	9.322×10^5	2.006×10^6	5.095×10^6	2.598×10^6	1.081×10^7	1.099×10^6	2.499×10^6	Positive
743	2-Methoxyestrone	$C_{19}H_{24}O_3$	300.17043	7.282	345.16869	3.288×10^6	3.777×10^6	3.895×10^6	3.124×10^6	3.984×10^6	3.025×10^6	3.777×10^6	Negative
744	Prostaglandin D3	$C_{20}H_{30}O_5$	332.19913	6.576	331.19185	8.193×10^6	6.093×10^6	6.460×10^5	8.101×10^6	6.186×10^5	5.991×10^5	6.014×10^5	Negative
745	Choline Glycerophosphate	$C_8H_{20}NO_6P$	257.10328	5.863	258.11087	2.025×10^6	2.035×10^6	3.255×10^6	2.457×10^6	2.741×10^6	5.743×10^6	6.407×10^6	Positive
746	Pinocembrin	$C_{15}H_{12}O_4$	256.07366	6.528	257.08094	6.677×10^5	6.933×10^5	1.074×10^7	8.733×10^5	1.039×10^7	5.976×10^5	5.864×10^5	Positive
747	Vindoline	$C_{25}H_{32}N_2O_6$	456.23375	6.309	439.23044	6.782×10^6	5.695×10^6	2.527×10^6	4.282×10^6	2.476×10^6	1.639×10^6	9.283×10^5	Positive
748	5-(tert-butyl)-N-(2,3-dihydro-1H-inden-2-yl)-2-methyl-3-furamide	$C_{19}H_{23}NO_2$	297.17338	6.103	296.1661	8.550×10^5	1.011×10^6	6.217×10^6	1.549×10^6	9.201×10^6	1.715×10^6	3.690×10^6	Negative

749	15(R)-Prostaglandin E2	$C_{20}H_{32}O_5$	352.22552	6.942	351.21825	6.701×10^{6}	5.196×10^{6}	1.075×10^{6}	5.807×10^{6}	4.416×10^{6}	4.865×10^{5}	4.614×10^{5}	Negative
750	LPG 18:3	$C_{24}H_{43}O_9P$	506.26474	8.842	505.25746	9.876×10^{6}	1.031×10^{7}	7.992×10^{5}	6.512×10^{5}	6.501×10^{5}	5.814×10^{5}	6.191×10^{5}	Negative
751	Galangin	$C_{15}H_{10}O_5$	116.05032	6.319	117.05763	1.031×10^{6}	1.026×10^{6}	5.397×10^{6}	1.909×10^{6}	9.752×10^{6}	3.038×10^{6}	9.401×10^{5}	Positive
752	Prostaglandin K2	$C_{20}H_{30}O_5$	332.19641	7.796	333.20371	1.949×10^{6}	1.749×10^{6}	4.851×10^{6}	1.753×10^{6}	9.632×10^{6}	1.616×10^{6}	1.515×10^{6}	Positive
753	11-dehydro Thromboxane B2	$C_{20}H_{32}O_6$	368.2202	8.072	367.21292	8.476×10^{6}	6.033×10^{6}	1.039×10^{6}	4.834×10^{6}	1.490×10^{6}	5.092×10^{5}	5.287×10^{5}	Negative
754	Thymidine 5'-diphosphate	$C_{10}H_{16}N_2O_{11}P_2$	402.02452	1.274	401.01724	6.220×10^{6}	5.053×10^{6}	1.792×10^{6}	5.386×10^{6}	1.498×10^{6}	1.388×10^{6}	1.466×10^{6}	Negative
755	Diflorasone	$C_{22}H_{28}F_2O_5$	410.19206	6.087	411.19934	1.024×10^{6}	1.009×10^{6}	3.743×10^{6}	7.660×10^{6}	5.547×10^{6}	2.609×10^{6}	1.024×10^{6}	Positive
756	2-(3,4-dimethoxyphenyl)quinoline	$C_{17}H_{15}NO_2$	265.11032	6.404	266.11772	7.437×10^{5}	6.885×10^{5}	9.061×10^{6}	9.460×10^{5}	7.411×10^{6}	5.101×10^{5}	3.243×10^{6}	Positive
757	Delta-Tridecalactone	$C_{13}H_{24}O_2$	212.17792	11.576	447.34497	4.312×10^{6}	3.288×10^{6}	3.159×10^{6}	2.492×10^{6}	3.026×10^{6}	2.330×10^{6}	3.933×10^{6}	Positive
758	Oleoyl-L-α-lysophosphatidic acid	$C_{21}H_{41}O_7P$	436.25975	10.563	435.25247	8.873×10^{6}	6.955×10^{6}	1.271×10^{6}	1.954×10^{6}	1.179×10^{6}	1.068×10^{6}	1.192×10^{6}	Negative
759	Cytidine 5'-Monophosphate-N-Acetylneuraminic Acid	$C_{20}H_{31}N_4O_{16}P$	614.14567	2.412	308.08011	5.272×10^{6}	7.721×10^{6}	3.803×10^{5}	6.146×10^{6}	1.736×10^{6}	5.158×10^{5}	3.718×10^{5}	Positive
760	7-alpha-carboxy-17-alpha-carboxyethylandrostan lactone phenyl ester	$C_{28}H_{38}O_5$	454.27015	8.872	455.27743	1.736×10^{6}	1.369×10^{6}	3.859×10^{6}	7.710×10^{5}	8.293×10^{6}	7.764×10^{5}	5.317×10^{6}	Positive
761	Cinchophen	$C_{16}H_{11}NO_2$	249.07895	6.731	250.0863	6.076×10^{5}	6.351×10^{5}	9.662×10^{6}	5.928×10^{5}	2.536×10^{6}	9.464×10^{5}	6.897×10^{6}	Positive
762	*cis*-7-Hexadecenoic Acid	$C_{16}H_{30}O_2$	254.22492	5.979	255.23219	6.711×10^{5}	8.464×10^{5}	4.902×10^{6}	1.261×10^{6}	6.680×10^{5}	8.584×10^{6}	4.746×10^{6}	Positive
763	5,7-dihydroxy-2-phenyl-4H-chromen-4-one	$C_{15}H_{10}O_4$	254.05863	5.844	255.0659	5.331×10^{5}	4.828×10^{5}	1.023×10^{7}	5.857×10^{5}	6.429×10^{6}	1.743×10^{6}	1.659×10^{6}	Positive
764	PC 18:3_18:3	$C_{44}H_{76}NO_8P$	823.54006	11.049	822.53279	2.608×10^{6}	7.652×10^{6}	5.693×10^{6}	2.044×10^{6}	9.366×10^{5}	1.765×10^{6}	9.370×10^{5}	Negative
765	4-methoxy-6-(prop-2-en-1-yl)-2H-1,3-benzodioxole	$C_{11}H_{12}O_3$	210.08754	5.303	193.08405	8.275×10^{6}	7.418×10^{6}	9.106×10^{5}	2.361×10^{6}	8.880×10^{5}	9.027×10^{5}	8.360×10^{5}	Positive
766	19(R)-Hydroxy-prostaglandin E2	$C_{20}H_{32}O_6$	350.20749	6.601	349.20021	7.269×10^{5}	2.009×10^{6}	4.462×10^{6}	8.986×10^{5}	1.258×10^{7}	4.064×10^{5}	4.476×10^{5}	Negative
767	Glycerol 1-hexadecanoate	$C_{19}H_{38}O_4$	330.2764	10.412	331.28333	3.429×10^{6}	3.309×10^{6}	3.822×10^{6}	3.138×10^{6}	3.003×10^{6}	2.548×10^{6}	2.238×10^{6}	Positive
768	Orotidine	$C_{10}H_{12}N_2O_8$	288.06069	1.625	289.06796	2.391×10^{6}	2.554×10^{6}	3.480×10^{6}	3.235×10^{6}	3.203×10^{6}	3.358×10^{6}	3.161×10^{6}	Positive
769	Feruloyl Putrescine	$C_{14}H_{20}N_2O_3$	264.14758	5.389	263.14041	3.641×10^{6}	4.344×10^{6}	2.128×10^{6}	4.742×10^{6}	1.090×10^{6}	1.961×10^{6}	3.326×10^{6}	Negative
770	13,14-dihydro-15-keto-tetranor Prostaglandin E2	$C_{16}H_{26}O_5$	298.1782	6.453	297.17092	1.187×10^{6}	1.060×10^{6}	4.850×10^{6}	1.082×10^{6}	1.179×10^{7}	5.847×10^{5}	5.685×10^{5}	Negative
771	2-(4-chlorophenoxy)-N-(2-oxo-3-azepanyl)nicotinamide	$C_{18}H_{18}ClN_3O_3$	359.10163	5.447	358.09435	3.744×10^{6}	5.302×10^{6}	1.793×10^{6}	5.103×10^{6}	1.801×10^{6}	1.650×10^{6}	1.674×10^{6}	Negative
772	NMK	$C_{15}H_{29}N_5O_5S$	373.17529	5.381	374.18288	6.809×10^{5}	4.952×10^{5}	3.766×10^{6}	5.416×10^{5}	3.935×10^{6}	1.225×10^{6}	1.035×10^{7}	Positive
773	N5-(2-chloro-6-phenoxybenzyl)-1H-1,2,4-triazole-3,5-diamine	$C_{15}H_{14}ClN_5O$	337.06908	11.692	338.07636	2.714×10^{6}	2.898×10^{6}	2.958×10^{6}	3.875×10^{6}	2.896×10^{6}	2.409×10^{6}	2.966×10^{6}	Positive
774	1-Methyluric acid	$C_6H_6N_4O_3$	182.0434	6.038	165.04006	3.426×10^{6}	3.535×10^{6}	3.230×10^{6}	3.247×10^{6}	2.074×10^{6}	2.907×10^{6}	2.238×10^{6}	Positive
775	Bilirubin	$C_{33}H_{36}N_4O_6$	584.26625	5.663	583.25897	1.641×10^{6}	1.983×10^{6}	2.986×10^{6}	2.235×10^{6}	5.804×10^{6}	3.373×10^{6}	2.151×10^{6}	Negative
776	4-Fluoro-N-(3-methyl-5-cinnolinyl)benzenesulfonamide	$C_{15}H_{12}FN_3O_2S$	317.06906	5.523	300.06592	4.934×10^{6}	2.240×10^{6}	2.929×10^{6}	3.285×10^{6}	2.432×10^{6}	2.389×10^{6}	1.865×10^{6}	Positive
777	Thymidine	$C_{10}H_{14}N_2O_5$	242.09046	4.914	265.07963	3.851×10^{6}	3.652×10^{6}	1.958×10^{6}	3.337×10^{6}	4.131×10^{6}	1.190×10^{6}	1.509×10^{6}	Positive
778	Phenylpropiolic acid	$C_9H_6O_2$	146.03712	1.208	169.02632	3.248×10^{6}	3.357×10^{6}	2.296×10^{6}	3.251×10^{6}	3.284×10^{6}	2.092×10^{6}	1.933×10^{6}	Positive

779	Trenbolone acetate	$C_{20}H_{24}O_3$	312.17221	6.554	313.17949	2.946×10^{6}	2.785×10^{6}	4.962×10^{6}	9.715×10^{5}	5.482×10^{6}	9.277×10^{5}	1.328×10^{6}	Positive
780	11β-Prostaglandin E2	$C_{20}H_{32}O_5$	334.21241	8.197	333.20513	1.506×10^{6}	1.455×10^{6}	3.459×10^{6}	9.513×10^{5}	1.073×10^{7}	5.761×10^{5}	5.841×10^{5}	Negative
781	Perfluorooctanoic acid (PFOA)	$C_8HF_{15}O_2$	413.97435	6.923	412.96707	2.627×10^{6}	3.176×10^{6}	2.855×10^{6}	2.550×10^{6}	2.867×10^{6}	2.756×10^{6}	2.397×10^{6}	Negative
782	LPI 18:2	$C_{27}H_{49}O_{12}P$	596.2974	9.488	595.29013	6.053×10^{6}	7.410×10^{6}	2.172×10^{6}	8.510×10^{5}	1.142×10^{6}	7.342×10^{5}	8.007×10^{5}	Negative
783	Methyl palmitate	$C_{17}H_{34}O_2$	287.28257	6.899	288.28985	2.785×10^{6}	3.212×10^{6}	2.350×10^{6}	3.092×10^{6}	3.052×10^{6}	2.446×10^{6}	2.191×10^{6}	Positive
784	Ala-trp	$C_{14}H_{17}N_3O_3$	275.1272	5.216	276.13447	1.622×10^{6}	2.096×10^{6}	4.406×10^{6}	3.952×10^{6}	2.140×10^{6}	2.754×10^{6}	2.092×10^{6}	Positive
785	LysoPE 18:0	$C_{23}H_{48}NO_7P$	481.31749	9.446	480.31021	6.513×10^{6}	7.905×10^{6}	6.680×10^{5}	2.140×10^{6}	6.112×10^{5}	5.985×10^{5}	6.039×10^{5}	Negative
786	JWH 018 N-(5-hydroxypentyl) β-D-Glucuronide	$C_{30}H_{31}NO_8$	555.19381	5.368	556.20096	2.270×10^{6}	2.295×10^{6}	1.764×10^{6}	2.861×10^{6}	4.625×10^{6}	3.309×10^{6}	1.638×10^{6}	Positive
787	*Dl*-3,4-Dihydroxymandelic Acid	$C_8H_8O_5$	184.03755	1.386	185.04482	2.160×10^{6}	3.048×10^{6}	2.482×10^{6}	2.540×10^{6}	3.414×10^{6}	2.303×10^{6}	2.739×10^{6}	Positive
788	8,15-Dihete	$C_{20}H_{32}O_4$	336.22992	6.708	335.22314	4.799×10^{6}	5.412×10^{6}	8.298×10^{5}	3.993×10^{6}	2.827×10^{6}	4.607×10^{5}	3.630×10^{5}	Negative
789	13,14-Dihydro-19(R)-hydroxy prostaglandin E1	$C_{20}H_{36}O_6$	372.25172	6.491	371.24445	1.711×10^{6}	1.419×10^{6}	9.043×10^{5}	1.776×10^{6}	5.447×10^{5}	4.003×10^{6}	7.861×10^{6}	Negative
790	4-Hydroxy-2-Oxoglutaric Acid	$C_5H_6O_6$	162.01633	2.025	161.00906	2.537×10^{6}	1.547×10^{6}	3.202×10^{6}	2.737×10^{6}	4.785×10^{6}	1.385×10^{6}	1.858×10^{6}	Negative
791	Trehalose 6-phosphate	$C_{12}H_{23}O_{14}P$	422.08288	1.278	423.09019	2.352×10^{6}	2.560×10^{6}	1.699×10^{6}	2.203×10^{6}	1.972×10^{6}	3.904×10^{6}	3.253×10^{6}	Positive
792	Limonin	$C_{26}H_{30}O_8$	487.21909	6.117	488.22636	1.445×10^{6}	1.424×10^{6}	1.517×10^{6}	1.413×10^{6}	1.588×10^{6}	8.856×10^{6}	1.428×10^{6}	Positive
793	N-Acetylglucosamine 1-phosphate	$C_8H_{16}NO_9P$	301.05567	5.876	300.04839	1.529×10^{6}	7.558×10^{5}	1.090×10^{6}	1.394×10^{6}	8.823×10^{5}	6.450×10^{6}	5.498×10^{6}	Negative
794	dTMP	$C_{10}H_{15}N_2O_8P$	322.05803	1.129	305.05497	1.923×10^{6}	1.753×10^{6}	2.626×10^{6}	3.153×10^{6}	2.637×10^{6}	2.570×10^{6}	2.671×10^{6}	Positive
795	ENK	$C_{15}H_{27}N_5O_7$	389.19503	5.729	390.20231	6.539×10^{5}	8.465×10^{5}	1.504×10^{6}	1.512×10^{6}	7.606×10^{5}	8.396×10^{6}	3.566×10^{6}	Positive
796	Erucic acid	$C_{22}H_{42}O_2$	338.31886	10.818	337.31158	9.304×10^{5}	9.692×10^{5}	1.004×10^{6}	9.259×10^{5}	9.731×10^{6}	2.394×10^{6}	9.206×10^{5}	Negative
797	Kuromanin	$C_{21}H_{20}O_{11}$	426.12033	9.635	449.10965	2.724×10^{6}	2.242×10^{6}	2.745×10^{6}	2.464×10^{6}	1.876×10^{6}	2.363×10^{6}	2.024×10^{6}	Positive
798	N1-[2-(4-chlorophenyl)-2-oxoethyl]-4-chlorobenzamide	$C_{15}H_{11}Cl_2NO_2$	258.13684	5.964	259.14412	8.587×10^{5}	8.570×10^{5}	3.952×10^{6}	1.111×10^{6}	5.492×10^{6}	1.812×10^{6}	2.203×10^{6}	Positive
799	N-[(5-methoxy-1H-indol-3-yl)methyl]-N,N-dimethylamine	$C_{12}H_{16}N_2O$	204.12791	5.44	447.2204	2.627×10^{6}	2.813×10^{6}	8.528×10^{5}	2.031×10^{6}	6.137×10^{6}	8.761×10^{5}	7.984×10^{5}	Positive
800	3-(3-Methoxyphenyl)propionic acid	$C_{10}H_{12}O_3$	180.07789	5.308	179.07076	4.084×10^{5}	4.108×10^{5}	3.869×10^{5}	4.287×10^{6}	9.804×10^{6}	3.730×10^{5}	3.672×10^{5}	Negative
801	3-(2-methylpropyl)-octahydropyrrolo[1,2-a]pyrazine-1,4-dione	$C_{11}H_{18}N_2O_2$	215.09466	5.721	216.10193	1.213×10^{6}	1.207×10^{6}	4.784×10^{6}	2.518×10^{6}	2.147×10^{6}	1.281×10^{6}	2.616×10^{6}	Positive
802	4-Aminobutyric acid	$C_4H_9NO_2$	103.06337	1.309	102.05606	2.110×10^{6}	2.407×10^{6}	2.048×10^{6}	3.857×10^{6}	1.773×10^{6}	1.849×10^{6}	1.663×10^{6}	Negative
803	9-benzyl-2,3,4,9-tetrahydro-1H-carbazol-4-one	$C_{19}H_{17}NO$	275.1312	6.833	276.13847	9.227×10^{5}	1.027×10^{6}	1.024×10^{7}	9.236×10^{5}	8.853×10^{5}	8.383×10^{5}	8.616×10^{5}	Positive
804	Epinephrine bitartrate	$C_{13}H_{19}NO_9$	333.10563	5.016	334.1129	4.232×10^{6}	3.074×10^{6}	1.264×10^{6}	2.484×10^{6}	2.104×10^{6}	1.537×10^{6}	9.312×10^{5}	Positive
805	Hex3Cer 63:12;2O	$C_{81}H_{133}NO_{18}$	2935.95503	2.086	1466.97024	1.568×10^{6}	1.621×10^{6}	1.842×10^{6}	1.484×10^{6}	1.572×10^{6}	1.487×10^{6}	5.988×10^{6}	Negative
806	Ergosta-5,7,9(11),22-Tetraen-3-beta-Ol	$C_{28}H_{42}O$	394.32362	6.38	395.33091	2.503×10^{6}	2.304×10^{6}	6.683×10^{5}	5.542×10^{6}	1.986×10^{6}	1.931×10^{6}	5.975×10^{5}	Positive
807	LysoPC 10:0	$C_{18}H_{38}NO_7P$	411.23686	6.09	412.24414	1.515×10^{6}	1.488×10^{6}	1.767×10^{6}	1.527×10^{6}	1.837×10^{6}	2.349×10^{6}	5.031×10^{6}	Positive
808	Solanine	$C_{45}H_{73}NO_{15}$	867.50084	11.029	868.50811	2.735×10^{6}	5.854×10^{6}	2.655×10^{6}	1.144×10^{6}	1.030×10^{6}	1.047×10^{6}	1.024×10^{6}	Positive
809	Carnosine	$C_9H_{14}N_4O_3$	226.10584	1.113	227.11311	2.633×10^{6}	2.585×10^{6}	1.573×10^{6}	3.066×10^{6}	1.660×10^{6}	2.342×10^{6}	1.567×10^{6}	Positive

810	Kynurenic acid O-hexside	$C_{16}H_{17}NO_8$	351.09593	5.541	350.08865	1.131×10^6	1.579×10^6	2.638×10^6	2.351×10^6	2.755×10^6	2.746×10^6	2.176×10^6	Negative
811	1-Methylguanine	$C_6H_7N_5O$	165.06498	4.915	166.07225	2.630×10^6	2.699×10^6	1.541×10^6	2.382×10^6	2.195×10^6	2.178×10^6	1.693×10^6	Positive
812	3-(4-morpholinophenyl)-2-(4-nitrophenyl)acrylonitrile	$C_{19}H_{17}N_3O_3$	335.13143	7.37	336.1387	6.580×10^5	6.607×10^5	9.081×10^6	6.494×10^5	3.022×10^6	5.532×10^5	6.801×10^5	Positive
813	1-Methyladenosine	$C_{11}H_{15}N_5O_4$	281.11145	1.999	282.11873	2.097×10^6	1.916×10^6	2.531×10^6	2.087×10^6	2.288×10^6	2.044×10^6	2.125×10^6	Positive
814	Dehydrocholic acid	$C_{24}H_{34}O_5$	402.2386	6.614	403.24588	2.684×10^6	1.367×10^6	1.719×10^6	1.580×10^6	5.269×10^6	1.666×10^6	7.221×10^5	Positive
815	6-phenyl-1,2,3,4-tetrahydro-2,5-benzodiazocin-1-one	$C_{16}H_{14}N_2O$	272.08743	4.746	255.08408	1.658×10^6	2.282×10^6	1.487×10^6	3.055×10^6	2.373×10^6	1.986×10^6	2.145×10^6	Positive
816	PC O-18:1	$C_{26}H_{52}NO_7P$	521.34958	9.442	544.33899	2.984×10^6	3.513×10^6	2.134×10^6	1.296×10^6	1.475×10^6	1.284×10^6	1.927×10^6	Positive
817	LPG 16:0	$C_{22}H_{45}O_9P$	484.28079	10.633	483.27351	4.455×10^6	5.216×10^6	1.018×10^6	9.749×10^5	9.570×10^5	8.465×10^5	9.334×10^5	Negative
818	Cholic acid	$C_{24}H_{40}O_5$	408.28569	10.359	409.29297	2.101×10^6	2.571×10^6	2.265×10^6	1.537×10^6	2.358×10^6	2.023×10^6	1.402×10^6	Positive
819	Sedoheptulose 1,7-bisphosphate	$C_7H_{16}O_{13}P_2$	370.00734	5.817	369.00006	1.973×10^6	2.163×10^6	1.982×10^6	1.862×10^6	2.201×10^6	1.765×10^6	1.986×10^6	Negative
820	Leucine-enkephalin	$C_{28}H_{37}N_5O_7$	555.26895	5.651	554.26168	1.967×10^6	1.732×10^6	2.216×10^6	1.945×10^6	2.068×10^6	1.938×10^6	1.991×10^6	Negative
821	EMK	$C_{16}H_{30}N_4O_6S$	384.2047	6.571	407.19383	6.994×10^5	6.817×10^5	4.929×10^6	6.660×10^5	4.729×10^6	5.550×10^5	1.520×10^6	Positive
822	Pyridoxine O-Glucoside	$C_{14}H_{21}NO_8$	331.12752	5.415	330.12	1.046×10^6	1.028×10^6	2.415×10^6	1.292×10^6	1.952×10^6	3.183×10^6	2.065×10^6	Negative
823	*L*-Threonic acid Calcium Salt	$C_8H_{14}CaO_{10}$	310.02176	1.162	309.01437	1.614×10^6	1.623×10^6	2.333×10^6	1.391×10^6	2.390×10^6	1.754×10^6	1.816×10^6	Negative
824	12-oxo Phytodienoic Acid	$C_{18}H_{28}O_3$	292.20296	6.646	310.23663	1.225×10^6	1.307×10^6	1.145×10^6	1.214×10^6	5.824×10^6	1.099×10^6	1.074×10^6	Positive
825	Tetrahydroaldosterone	$C_{21}H_{32}O_5$	364.22316	9.134	363.21588	2.107×10^6	2.011×10^6	1.981×10^6	1.427×10^6	2.116×10^6	1.708×10^6	1.433×10^6	Negative
826	2-(cyclopropylcarbonyl)-3-(4-fluoroanilino)acrylonitrile	$C_{13}H_{11}FN_2O$	252.06235	4.798	253.06963	1.555×10^6	1.970×10^6	1.388×10^6	2.259×10^6	2.405×10^6	1.928×10^6	1.243×10^6	Positive
827	4-amino-2-(dibenzylamino)-5-pyrimidinecarbonitrile	$C_{19}H_{17}N_5$	315.14458	6.344	316.15186	7.427×10^5	7.274×10^5	1.706×10^6	6.525×10^5	6.127×10^5	5.936×10^5	7.646×10^6	Positive
828	5,6-Dihydroxyindole-2-Carboxylic Acid	$C_9H_7NO_4$	193.03776	5.301	238.036	9.638×10^5	9.237×10^5	3.890×10^6	9.182×10^5	9.163×10^5	1.019×10^6	3.845×10^6	Negative
829	1,3-diazaspiro[4.5]decane-2,4-dione	$C_8H_{12}N_2O_2$	168.0873	1.125	169.09396	2.569×10^6	2.946×10^6	1.154×10^6	1.321×10^6	1.109×10^6	2.017×10^6	1.137×10^6	Positive
830	methyl 3,4,5-trihydroxycyclohex-1-ene-1-carboxylate	$C_8H_{12}O_5$	210.05096	7.4	211.05823	1.741×10^6	2.096×10^6	1.408×10^6	1.962×10^6	1.610×10^6	1.999×10^6	1.301×10^6	Positive
831	YPH	$C_{20}H_{25}N_5O_5$	437.16924	4.842	438.17656	4.197×10^5	4.401×10^5	1.716×10^6	1.155×10^6	1.187×10^6	4.567×10^6	2.558×10^6	Positive
832	11(Z)-Eicosenoic acid	$C_{20}H_{38}O_2$	356.29319	9.89	355.28591	3.835×10^6	5.261×10^6	5.230×10^5	1.008×10^6	4.867×10^5	4.348×10^5	4.693×10^5	Negative
833	NNH	$C_{14}H_{21}N_7O_6$	383.15248	6.964	384.15976	6.328×10^5	6.830×10^5	2.319×10^6	6.182×10^5	6.508×10^6	5.418×10^5	5.701×10^5	Positive
834	2,3-dinor Prostaglandin E1	$C_{18}H_{30}O_5$	308.19659	7.791	307.18932	1.408×10^6	1.143×10^6	1.982×10^6	1.062×10^6	4.817×10^6	7.751×10^5	6.637×10^5	Negative
835	18-β-Glycyrrhetinic acid	$C_{30}H_{46}O_4$	470.33989	9.738	469.33261	5.518×10^5	5.733×10^5	1.241×10^6	5.697×10^5	7.861×10^6	4.988×10^5	5.471×10^5	Negative
836	1,3-Dimethyluracil	$C_6H_8N_2O_2$	140.05876	5.291	303.10703	1.919×10^6	7.562×10^5	9.980×10^5	4.651×10^6	1.203×10^6	1.074×10^6	9.321×10^5	Positive
837	Guanethidine Monosulfate	$C_{10}H_{24}N_4O_4S$	296.1522	5.967	297.15948	1.033×10^6	1.031×10^6	1.381×10^6	1.099×10^6	2.044×10^6	2.180×10^6	2.649×10^6	Positive
838	(+/-)-Cannabichromeorcin	$C_{17}H_{22}O_2$	258.16209	7.926	257.15481	8.559×10^5	6.856×10^5	1.586×10^6	4.818×10^5	4.363×10^6	4.358×10^5	2.962×10^6	Negative
839	*L*-Canavanine	$C_5H_{12}N_4O_3$	176.09038	1.152	175.0831	1.283×10^6	1.161×10^6	2.251×10^6	1.358×10^6	2.098×10^6	1.346×10^6	1.627×10^6	Negative
840	di{4-[(2-hydroxyethyl)(methyl)amino]phenyl}methanone	$C_{19}H_{24}N_2O_3$	328.17781	5.714	329.18509	1.288×10^6	1.283×10^6	1.741×10^6	1.648×10^6	1.902×10^6	1.548×10^6	1.453×10^6	Positive
841	Methyltestosterone	$C_{20}H_{30}O_2$	302.22435	6.85	303.23163	1.402×10^6	1.396×10^6	9.723×10^5	2.132×10^6	9.153×10^5	1.396×10^6	2.581×10^6	Positive

842	Tazobactam sodium	$C_{10}H_{12}N_4O_5S$	300.05009	1.993	301.05737	8.409×10^5	8.626×10^5	1.651×10^6	1.373×10^6	1.010×10^6	1.985×10^6	2.994×10^6	Positive
843	3-(3,4,5-trimethoxyphenyl)propanoic acid	$C_{12}H_{16}O_5$	112.06428	6.456	263.09186	1.354×10^6	1.725×10^6	1.513×10^6	1.653×10^6	1.677×10^6	1.380×10^6	1.257×10^6	Positive
844	Bicyclo Prostaglandin E2	$C_{20}H_{30}O_4$	316.20439	8.091	315.19711	1.924×10^6	1.263×10^6	1.251×10^6	1.637×10^6	2.497×10^6	5.759×10^5	1.398×10^6	Negative
845	Tangeritin	$C_{20}H_{20}O_7$	372.12089	6.841	373.12823	7.277×10^5	8.114×10^5	5.987×10^6	8.897×10^5	6.720×10^5	7.501×10^5	6.686×10^5	Positive
846	Ergothioneine	$C_9H_{15}N_3O_2S$	229.08814	1.123	230.09541	1.500×10^6	1.221×10^6	1.743×10^6	1.813×10^6	1.629×10^6	1.188×10^6	1.404×10^6	Positive
847	2,5-bis(4-hydroxy-3-methoxyphenyl)-3,4-dimethyloxolan-3-ol	$C_{20}H_{24}O_6$	382.13746	4.144	383.14474	6.121×10^5	4.623×10^5	3.380×10^6	8.877×10^5	1.491×10^6	4.080×10^5	3.162×10^6	Positive
848	LPG 18:1	$C_{24}H_{47}O_9P$	510.29606	10.674	509.28879	2.849×10^6	2.384×10^6	1.108×10^6	1.063×10^6	1.032×10^6	9.400×10^5	9.942×10^5	Negative
849	Soyasaponin I	$C_{48}H_{78}O_{18}$	942.52509	7.029	941.51781	4.818×10^5	5.088×10^5	4.686×10^6	4.963×10^5	3.209×10^6	4.431×10^5	4.895×10^5	Negative
850	Lysopa 16:0	$C_{19}H_{39}O_7P$	410.24249	7.819	411.24976	1.654×10^6	8.684×10^5	2.179×10^6	1.833×10^6	1.164×10^6	1.002×10^6	1.319×10^6	Positive
851	(2E)-3-phenyl-N-(2-phenylethyl)prop-2-enamide	$C_{17}H_{17}NO$	273.11553	7.212	274.12281	4.589×10^5	4.864×10^5	5.013×10^6	4.687×10^5	2.758×10^6	4.135×10^5	4.154×10^5	Positive
852	[1-(3-nitro-2-pyridyl)-4-piperidyl](phenyl)methanone	$C_{17}H_{17}N_3O_3$	289.14271	5.47	290.15005	8.865×10^5	9.032×10^5	9.377×10^5	9.216×10^5	9.309×10^5	9.332×10^5	4.314×10^6	Positive
853	(+)-Evodiamine	$C_{19}H_{17}N_3O$	303.14256	4.924	304.14984	7.316×10^5	1.607×10^6	1.946×10^6	1.415×10^6	1.369×10^6	1.034×10^6	1.692×10^6	Positive
854	*D*-Threose	$C_4H_8O_4$	120.04271	1.792	121.04999	1.830×10^6	1.543×10^6	6.524×10^5	1.278×10^6	9.603×10^5	1.764×10^6	1.726×10^6	Positive
855	Cystine	$C_6H_{12}N_2O_4S_2$	240.02269	6.597	241.02996	1.716×10^6	1.651×10^6	8.678×10^5	1.556×10^6	9.612×10^5	1.570×10^6	1.416×10^6	Positive
856	Lysopg 18:1	$C_{24}H_{47}O_9P$	510.29726	6.592	511.30453	1.155×10^6	1.059×10^6	1.090×10^6	2.375×10^6	2.331×10^6	9.896×10^5	6.816×10^5	Positive
857	Lenalidomide	$C_{13}H_{13}N_3O_3$	276.11919	6.076	277.12646	8.206×10^5	7.568×10^5	1.617×10^6	1.532×10^6	2.721×10^6	1.302×10^6	7.440×10^5	Positive
858	cholesteryl sulfate	$C_{27}H_{46}O_4S$	466.31158	10.108	505.27531	1.147×10^6	1.542×10^6	2.163×10^6	9.469×10^5	2.366×10^6	5.897×10^5	6.732×10^5	Positive
859	DG O-20:2_20:1	$C_{43}H_{80}O_4$	677.62996	11.384	678.63723	6.297×10^5	9.812×10^5	2.076×10^6	5.981×10^5	3.041×10^6	5.213×10^5	1.398×10^6	Positive
860	N2-tetrahydrofuran-2-ylmethyl-4-(4-fluorophenyl)-1,3-thiazol-2-amine	$C_{14}H_{15}FN_2OS$	256.11026	6.273	257.11749	7.014×10^5	6.945×10^5	2.637×10^6	6.798×10^5	3.232×10^6	6.304×10^5	6.108×10^5	Positive
861	13,14-dihydro-15-keto Prostaglandin D1	$C_{20}H_{34}O_5$	336.2279	8.627	335.22063	5.498×10^5	6.320×10^5	1.583×10^6	5.083×10^5	4.651×10^6	4.477×10^5	4.814×10^5	Negative
862	beta-Estradiol 17-Acetate	$C_{20}H_{26}O_3$	314.18898	6.077	315.19625	6.802×10^5	6.718×10^5	2.293×10^6	1.093×10^6	1.646×10^6	1.135×10^6	1.134×10^6	Positive
863	2-deoxyglucose-6-phosphate	$C_6H_{13}O_8P$	244.03501	1.224	245.04219	1.863×10^6	1.442×10^6	1.218×10^6	1.534×10^6	1.158×10^6	6.103×10^5	6.891×10^5	Positive
864	Betulin	$C_{30}H_{50}O_2$	442.38148	7.67	443.38884	5.695×10^5	6.065×10^5	5.490×10^5	5.792×10^5	5.006×10^6	5.027×10^5	5.509×10^5	Positive
865	N1,N1-diethyl-4-[5-(4-nitrophenyl)-1,3-oxazolan-2-yl]aniline	$C_{19}H_{23}N_3O_3$	341.17808	7.091	342.18532	4.407×10^5	4.819×10^5	5.106×10^6	4.493×10^5	1.026×10^6	3.980×10^5	4.137×10^5	Positive
866	3-(5-chloro-2-thienyl)-2-(methylsulfonyl)acrylonitrile	$C_8H_6ClNO_2S_2$	263.97793	9.707	264.98521	1.170×10^6	1.172×10^6	1.040×10^6	1.385×10^6	1.104×10^6	1.414×10^6	9.989×10^5	Positive
867	2-Deoxyuridine	$C_9H_{12}N_2O_5$	228.07448	4.985	229.08176	8.136×10^5	7.146×10^5	6.574×10^5	3.563×10^6	8.420×10^5	5.600×10^5	1.088×10^6	Positive
868	5,6-dimethyl-3-[5-(trifluoromethyl)pyridin-2-yl]-1,2,4-triazine	$C_{11}H_9F_3N_4$	254.07697	10.402	255.08424	1.129×10^6	1.323×10^6	1.060×10^6	1.211×10^6	1.064×10^6	1.502×10^6	8.565×10^5	Positive
869	N-allyl-2-(2-methyl-1-benzofuran-3-yl)acetamide	$C_{14}H_{15}NO_2$	251.09259	5.278	252.09986	1.004×10^6	9.377×10^5	1.280×10^6	1.579×10^6	2.134×10^6	6.154×10^5	5.339×10^5	Positive
870	Methandrostenolone	$C_{20}H_{28}O_2$	300.20897	8.14	323.19835	7.307×10^5	8.712×10^5	1.073×10^6	4.501×10^5	3.017×10^6	4.174×10^5	1.522×10^6	Positive

871	LPE 18:1	$C_{23}H_{46}NO_7P$	479.30128	9.446	478.29401	1.782×10⁶	2.201×10⁶	1.131×10⁶	7.136×10⁵	9.222×10⁵	5.429×10⁵	6.656×10⁵	Negative
872	*Dl*-Lanthionine	$C_6H_{12}N_2O_4S$	208.05266	5.764	209.05994	6.122×10⁵	1.704×10⁶	1.220×10⁶	6.216×10⁵	7.815×10⁵	7.769×10⁵	2.225×10⁶	Positive
873	6-methoxy-4-(trifluoromethyl)nicotinamide	$C_8H_7F_3N_2O_2$	242.02739	6.682	243.03467	1.599×10⁶	9.981×10⁵	1.491×10⁶	1.063×10⁶	6.856×10⁵	1.287×10⁶	8.136×10⁵	Positive
874	N-Desmethyltramadol	$C_{15}H_{23}NO_2$	232.14617	6.242	233.15353	1.080×10⁶	8.075×10⁵	1.123×10⁶	1.276×10⁶	2.257×10⁶	7.330×10⁵	5.399×10⁵	Positive
875	aminoimidazole carboxamide ribonucleotide	$C_9H_{15}N_4O_8P$	338.06184	1.285	337.0544	7.014×10⁵	7.439×10⁵	9.990×10⁵	7.338×10⁵	7.818×10⁵	1.581×10⁶	2.261×10⁶	Negative
876	Chlorogenic Acid Methyl Ester	$C_{17}H_{20}O_9$	368.10906	5.04	386.14331	1.056×10⁶	6.964×10⁵	2.364×10⁶	7.147×10⁵	1.757×10⁶	4.418×10⁵	5.301×10⁵	Positive
877	Gamma-Glu-Leu	$C_{11}H_{20}N_2O_5$	260.13763	4.044	261.14502	1.190×10⁶	1.018×10⁶	6.024×10⁵	2.759×10⁶	6.132×10⁵	5.845×10⁵	5.916×10⁵	Positive
878	3-Acetyl-11-keto-β-boswellic acid	$C_{32}H_{48}O_5$	512.34855	9.208	513.35582	5.393×10⁵	5.777×10⁵	1.482×10⁶	5.613×10⁵	3.073×10⁶	5.037×10⁵	5.036×10⁵	Positive
879	Triiodothyronine	$C_{15}H_{12}I_3NO_4$	650.78903	1.222	651.79631	1.157×10⁶	1.228×10⁶	9.726×10⁵	1.070×10⁶	1.888×10⁶	2.321×10⁵	3.806×10⁵	Positive
880	Quinolinic acid	$C_7H_5NO_4$	167.02196	8.566	166.01468	1.421×10⁶	1.113×10⁶	1.554×10⁶	9.190×10⁵	6.363×10⁵	6.250×10⁵	6.530×10⁵	Negative
881	Triacanthine	$C_{10}H_{13}N_5$	203.11649	6.184	407.24094	8.359×10⁵	8.886×10⁵	1.154×10⁶	9.970×10⁵	1.131×10⁶	9.472×10⁵	8.295×10⁵	Positive
882	Trehalose	$C_{12}H_{22}O_{11}$	342.11638	2.009	343.12366	1.175×10⁶	1.475×10⁶	6.046×10⁵	6.365×10⁵	6.503×10⁵	1.553×10⁶	5.704×10⁵	Positive
883	Nobiletin	$C_{21}H_{22}O_8$	402.13185	6.532	403.13926	5.947×10⁵	6.175×10⁵	2.790×10⁶	8.281×10⁵	5.223×10⁵	6.614×10⁵	5.223×10⁵	Positive
884	4-(cyclohexylmethyl)-6-(2-thienyl)-2,3-dihydropyridazin-3-one hydrate	$C_{15}H_{18}N_2OS$	296.10076	4.811	297.10804	7.901×10⁵	8.026×10⁵	7.418×10⁵	1.881×10⁶	7.492×10⁵	7.339×10⁵	7.310×10⁵	Positive
885	Fenpropimorph	$C_{20}H_{33}NO$	303.25394	11.987	304.26122	7.217×10⁵	1.846×10⁶	1.376×10⁶	6.856×10⁵	6.429×10⁵	4.911×10⁵	5.443×10⁵	Positive
886	*D*-Fructose 6-phosphate	$C_6H_{13}O_9P$	260.03011	1.857	261.03738	1.205×10⁶	1.065×10⁶	7.792×10⁵	9.792×10⁵	9.376×10⁵	8.477×10⁵	4.781×10⁵	Positive
887	Fmoc-L-Isoleucine	$C_{21}H_{23}NO_4$	353.16158	6.577	354.16885	7.094×10⁵	7.477×10⁵	7.179×10⁵	7.072×10⁵	2.001×10⁶	6.351×10⁵	6.073×10⁵	Positive
888	*L*-Dihydroorotic Acid	$C_5H_6N_2O_4$	158.03293	2.224	157.02565	5.785×10⁵	5.842×10⁵	6.302×10⁵	5.952×10⁵	6.360×10⁵	5.236×10⁵	2.475×10⁶	Negative
889	19-Nortestosterone	$C_{18}H_{26}O_2$	274.19202	7.397	275.1993	1.282×10⁶	8.502×10⁵	7.773×10⁵	1.140×10⁶	4.858×10⁵	9.038×10⁵	5.494×10⁵	Positive
890	Vitamin B2	$C_{17}H_{20}N_4O_6$	376.13802	6.693	377.14529	6.277×10⁵	6.501×10⁵	5.871×10⁵	6.080×10⁵	9.980×10⁵	1.508×10⁶	5.512×10⁵	Positive
891	5'-Adenylic acid	$C_{10}H_{14}N_5O_7P$	347.06326	1.877	348.07054	6.939×10⁵	6.471×10⁵	1.700×10⁶	4.227×10⁵	1.121×10⁶	4.251×10⁵	3.905×10⁵	Positive
892	3-Nitro-L-Tyrosine	$C_9H_{10}N_2O_5$	226.05894	1.97	227.06622	4.993×10⁵	5.173×10⁵	9.182×10⁵	4.778×10⁵	1.312×10⁶	4.307×10⁵	8.369×10⁵	Positive
893	Methyl cinnamate	$C_{10}H_{10}O_2$	162.06565	5.475	163.07256	6.174×10⁵	1.127×10⁶	6.217×10⁵	6.326×10⁵	6.490×10⁵	6.394×10⁵	5.806×10⁵	Positive
894	4-Pyridoxic acid	$C_8H_9NO_4$	183.05299	2.336	182.04571	4.780×10⁵	5.330×10⁵	5.969×10⁵	4.781×10⁵	5.256×10⁵	7.166×10⁵	1.082×10⁶	Negative
895	2-[(butylamino)(imino)methyl]-1-oxohydrazinium-1-olate	$C_5H_{12}N_4O_2$	182.07942	10.485	183.0867	5.218×10⁵	5.700×10⁵	6.087×10⁵	6.429×10⁵	5.638×10⁵	7.314×10⁵	4.802×10⁵	Positive
896	15(S)-HpETE	$C_{20}H_{32}O_4$	318.21934	6.35	319.22662	7.747×10⁵	8.424×10⁵	5.482×10⁵	8.278×10⁵	3.447×10⁵	3.471×10⁵	4.051×10⁵	Positive
897	Nicotinuric Acid	$C_8H_8N_2O_3$	180.05357	2.808	181.06085	7.296×10⁵	7.784×10⁵	2.687×10⁵	1.549×10⁶	2.528×10⁵	2.384×10⁵	2.541×10⁵	Positive
898	*D*-Ribulose 1,5-bisphosphate	$C_5H_{12}O_{11}P_2$	309.98657	6.549	308.97929	5.320×10⁵	5.624×10⁵	5.481×10⁵	5.329×10⁵	8.079×10⁵	4.953×10⁵	5.375×10⁵	Negative
899	Heptadecanoic Acid	$C_{17}H_{34}O_2$	292.23978	8.312	293.24706	8.110×10⁵	5.949×10⁵	6.494×10⁵	4.449×10⁵	4.830×10⁵	6.007×10⁵	3.742×10⁵	Positive
900	Biocytin	$C_{16}H_{28}N_4O_4S$	372.18825	6.459	373.19553	2.167×10⁵	2.202×10⁵	6.104×10⁵	2.049×10⁵	8.448×10⁵	1.455×10⁶	3.556×10⁵	Positive
901	Adipamide	$C_6H_{12}N_2O_2$	144.08984	4.826	145.09711	3.185×10⁵	2.923×10⁵	8.612×10⁵	4.271×10⁵	6.395×10⁵	9.450×10⁵	2.651×10⁵	Positive

902	FAHFA 18:2/20:4	$C_{38}H_{62}O_4$	582.46357	10.114	581.45629	3.347×10^5	3.616×10^5	4.125×10^5	3.269×10^5	1.593×10^6	2.993×10^5	3.139×10^5	Negative
903	3-Hydroxypicolinic acid	$C_6H_5NO_3$	139.02712	4.893	140.0344	4.436×10^5	6.911×10^5	4.504×10^5	4.999×10^5	4.362×10^5	3.938×10^5	5.549×10^5	Positive
904	2-Hydroxybutyric acid	$C_4H_8O_3$	104.04784	9.166	105.05511	4.141×10^5	4.020×10^5	4.227×10^5	8.322×10^5	4.636×10^5	4.100×10^5	3.169×10^5	Positive
905	geranyl pp	$C_{10}H_{20}O_7P_2$	314.0673	8.447	315.07458	3.402×10^5	3.146×10^5	3.777×10^5	6.525×10^5	2.652×10^5	2.724×10^5	2.950×10^5	Positive
906	Benzophenone	$C_{13}H_{10}O$	182.07115	1.22	183.07843	4.364×10^5	5.087×10^5	1.752×10^5	3.979×10^5	5.273×10^5	1.475×10^5	1.597×10^5	Positive
907	2-Furoic acid	$C_5H_4O_3$	112.01424	3.313	113.02152	3.817×10^5	4.241×10^5	2.557×10^5	2.465×10^5	3.111×10^5	1.869×10^5	4.547×10^5	Positive
908	Anserine	$C_{10}H_{16}N_4O_3$	240.12119	0.544	241.12826	5.850×10^5	2.817×10^5	2.527×10^5	2.638×10^5	2.219×10^5	2.334×10^5	2.390×10^5	Positive
909	N,5-Bis(3-nitrophenyl)oxazol-2-amine	$C_{15}H_{10}N_4O_5$	326.06113	4.887	327.06841	2.131×10^5	4.483×10^5	1.697×10^5	1.880×10^5	1.686×10^5	1.619×10^5	1.699×10^5	Positive
910	2-(Phenylsulfonyl)aniline	$C_{12}H_{11}NO_2S$	233.04807	2.458	234.05535	9.735×10^4	7.346×10^4	1.037×10^5	1.106×10^5	1.314×10^5	2.093×10^5	2.702×10^5	Positive
